# Categorial Compositionality II: Universal Constructions and a General Theory of (Quasi-)Systematicity in Human Cognition

**DOI:** 10.1371/journal.pcbi.1002102

**Published:** 2011-08-04

**Authors:** Steven Phillips, William H. Wilson

**Affiliations:** 1 Mathematical Neuroinformatics Group, Human Technology Research Institute, National Institute of Advanced Industrial Science and Technology (AIST), Tsukuba, Ibaraki, Japan; 2 School of Computer Science and Engineering, The University of New South Wales, Sydney, New South Wales, Australia; Indiana University, United States of America

## Abstract

A complete theory of cognitive architecture (i.e., the basic processes and modes of composition that together constitute cognitive behaviour) must explain the systematicity property—why our cognitive capacities are organized into particular groups of capacities, rather than some other, arbitrary collection. The classical account supposes: (1) syntactically compositional representations; and (2) processes that are sensitive to—compatible with—their structure. Classical compositionality, however, does not explain why these two components must be compatible; they are only compatible by the ad hoc assumption (convention) of employing the same mode of (concatenative) compositionality (e.g., prefix/postfix, where a relation symbol is always prepended/appended to the symbols for the related entities). Architectures employing mixed modes do not support systematicity. Recently, we proposed an alternative explanation without ad hoc assumptions, using category theory. Here, we extend our explanation to domains that are quasi-systematic (e.g., aspects of most languages), where the domain includes some but not all possible combinations of constituents. The central category-theoretic construct is an adjunction involving pullbacks, where the primary focus is on the relationship between processes modelled as functors, rather than the representations. A functor is a structure-preserving map (or construction, for our purposes). An adjunction guarantees that the only pairings of functors are the systematic ones. Thus, (quasi-)systematicity is a necessary consequence of a categorial cognitive architecture whose basic processes are functors that participate in adjunctions.

## Author Summary

John, your greengrocer, arranges apples and oranges in order of price. Being on a budget, you choose the fruit to the left, because it is always cheaper. Last week it was apples; this week it's oranges. Your ability to choose the fruit on the left, be it apples or oranges, is an example of systematicity: a property of human cognition whereby having some cognitive abilities means having certain others. Explaining why cognitive ability is organized this way is a basic question for cognitive science. Cognitive scientists generally agree that systematicity depends on some form of structured representations. However, they have been unable to provide a complete answer without relying on some overly strong assumptions about the nature of these representations. We provide an alternative explanation, using a mathematical theory of structure called Category Theory. Our previous work dealt with a special case, where ability extends to all possible combinations of constituents. Some domains (e.g., aspects of language) are only quasi-systematic: capacity extends to some but not all possible combinations. The current work presents our more general theory of (quasi-)systematicity, which includes our previous explanation as a special case.

## Introduction

A complete theory of human cognition must explain why our mental abilities are organized into particular groups of cognitive capacities, rather than some arbitrary, random collection. For example, if one can infer that the left block is blue on seeing a (blue, red) pair of blocks, then necessarily one also has the capacity to infer that the left block is red on seeing a (red, blue) pair. This property of cognitive architecture (i.e., the collection of basic processes and modes of composition that together generate cognitive behaviour) is called *systematicity* [1], and the problem posed for a theory of cognition is to explain why systematicity is a necessary consequence of the assumptions and principles embodied by the architecture that the proposed theory posits [1], [2].

The classical explanation derives from the principle of *classical compositionality*, which says that cognitive representations and processes are constructed from a combinatorial syntax and semantics, whereby semantic relations between constituents of the complex entities represented by a cognitive system are mirrored by syntactic relations between the corresponding constituent representations–that is, syntactically structured representations *and* processes that are sensitive to (i.e., compatible with) those structures [1].

To illustrate this principle and the intended classical explanation for systematicity, a (blue, red) pair of blocks is represented by a (BLUE, RED) pair of symbols, such that the semantic spatial relation *left-of*, relating the blue and red constituent blocks, is mirrored by a syntactic order relation *predecessor-of*, relating the corresponding BLUE and RED constituent symbols representing those blocks; and inferring the left block is realized by a process for identifying the preceding (first) symbol of a pair of symbols. The capacity to infer the left block as blue from (blue, red) implies the capacity to infer the left block as red from (red, blue), *assuming* the two inferences involve one and the same process. Thus, the presence or absence of this process as part of the system's architecture realizes the presence or absence of both inferential capacities; there is no case of having one capacity without having the other. Hence, this systematicity of block pairs is a consequence of this architecture.

The problem for the classical explanation, which echoes the essential problem already identified with the connectionist explanation [1], [3], is that the core principle of the theory (i.e., classical compositionality) is not sufficient to explain systematicity: although classical systems can be configured to realize a particular form of systematicity, classical systems can also be configured so as not to realize that form of systematicity from that same classical principle [2] (see also [4], for an example). Thus, having a combinatorial syntax and semantics is not a sufficient explanation for the systematicity of human cognition. Additional (*ad hoc*) assumptions are employed to remedy this situation, and so classical (and connectionist) compositionality fails to fully explain the systematicity of human cognition [2].

The crux of the problem is that the two parts of the classical compositionality principle, that is: (1) combinatorial syntax and semantics, and (2) structure-sensitive processes, are only made compatible by the *ad hoc* assumption (convention) that they respect the same mode of (concatenative) compositionality (e.g., prefix/infix/postfix, where a relation symbol is always prepended/infixed/appended to the symbols for the related entities); there is no explanation as to *why* these two components *must* be compatible (see [2], ch.4, for detailed discussion, and an alternative illustration). (Previously [4], we simply highlighted a problem for classical theory on the constructive side. Here, we highlight the more general problem to emphasize what our approach is intended to explain, and how it contrasts with the classical theory.) By convention, one may assume an infix mode of classical concatenative compositionality, whereby *John loves Mary* is represented by [John Loves Mary]. Yet, by convention, one may also choose a prefix mode, e.g., [Loves John Mary], or a postfix mode, e.g., [John Mary Loves], as employed in some (programming) languages, or even one where argument order is reversed, e.g., [Mary Loves John], where Mary is the beloved and John is the lover. All these possibilities are characteristically classical in that the representations of constituent entities are *tokened* (instantiated) whenever the representations of their complex hosts are [1], [3]. The problem is that although a representation of, say, constituent *John* is tokened in a representation of complex host *John loves Mary*, it is not necessarily tokened as the lover with respect to a process intended to make that inference. An architecture that employs incompatible combinations will not exhibit systematicity. Classical compositionality does not fully explain systematicity because of the *ad hoc* assumption that only certain combinations are permitted. This assumption is enforced by the cognitive scientist not the cognitive system. For an extended discussion on the problem of *ad hoc* assumptions in science generally, and classical/connectionist explanations of systematicity specifically, see [2]. (At this point, modellers may think to augment their theory with some sort of learning principle, such as is commonly incorporated into connectionist [5] and Bayesian modeling [6]. However, connectionist and Bayesian approaches suffer the same shortcoming as the classical approach: while both are capable of configuring architectures with the desired form of systematicity, they likewise permit architectures without that form of systematicity. See also [4], on this point.)

Recently, we presented an alternative explanation for systematicity without recourse to such (*ad hoc*) assumptions [4] that employed a branch of mathematics called category theory [7], where the theoretical focus is on the relationships between structure-sensitive processes, rather than the representations on which they operate. In particular, the category theory notion of *functor* maps (generalizations of) functions to (generalized) functions, as well as mapping objects to objects. The central explanatory element in [4] is the formal category theory concept of *adjunction*: an adjunction relates two functorial constructions so that of the *possibly* systematic capacity-realizing constructions there is one and only one construction that realizes all systematically related capacities via the adjunction. Hence, no further, *ad hoc*, assumptions are required to distinguish the systematic from unsystematic architectures, thus meeting the explanatory standard for systematicity in human cognition originally explicated in [1], and subsequently clarified in [2]. In our theory, basic building blocks of human cognitive architecture involve adjunctive relationships between functorial constructions.

Outside our use of adjunction to explain systematicity in [4], adjunctions do not appear to have been used in cognitive science (but, see [8] for a conceptual introduction; see also [9] in the context of general systems theory of abstract machines and behaviours). To provide some orientation, one may think of the classical and connectionist approaches as primarily focussed on the processes that transform representations, at the expense of being unable to guarantee a systematic relationship between those processes. In contrast, an adjunction guarantees that the only pairings of functors modeling such processes are the systematic ones. Thus, systematicity follows without further, *ad hoc* assumptions.

Our explanation of systematicity was applied in two domains that involved cognitive capacities pertaining to (1) a common relation, and (2) a common relational schema. With respect to these domains, human cognition exhibits what we may call “full” systematicity, in the sense that capacity is extended to each and every combination of the possible constituents that may partake in the relation or schema. For example, suppose one has the capacity to represent entities *John*, *Mary*, *Sue*, *Tom*, and *loves*, and the relational proposition that *John loves Mary*, then one has the capacity to represent all possible combinations, such as *Sue loves Tom*, *Tom loves John*, *Mary loves Mary*, and so on.

In fact, not all domains are fully (completely) systematic. Additional constraints relevant to the domain of interest preclude particular combinations. In particular, linguistic constructions often incorporate different types of constraints, including syntactic, phonetic, semantic, and pragmatic constraints that may further restrict the group of capacities that are intrinsically connected [10]. For example, English-speakers say *John put his gear down*, but not *John stowed his gear down*, even though they say *John put his gear away*, or *John stowed his gear away* (see [10] for this and other examples). Such cases may be regarded as examples of quasi-systematicity, in the sense that we will detail next. Our purpose in this paper is to show how our category theory explanation of systematicity [4] generalizes to include quasi-systematicity.

### Quasi-systematicity and the distribution of cognitive capacity

Surrounding the debate over the implications of systematicity for theories of cognitive architecture are a number of misconceptions as to what is in need of explaining and what counts as an explanation (see [2] for a detailed review). One commonly held misconception is that human cognition is rarely “systematic” in that many cognitive domains include exceptional cases not covered by the (classical) theory. Therefore, according to this view, systematicity says little, if anything, about cognition. This view asserts that systematicity pertains to relatively simple (structural) relationships between cognitive capacities, and thereby overlooks the possibility that cognitive capacities depend on more complex relationships (see, e.g., [1], p.29). This more complex relationship between cognitive capacities is what we are generally referring to as *quasi-systematicity*. Here, we expand upon this difference to clarify what is in need of explaining and to motivate our general theory of (quasi-)systematicity.

As a property of cognition, systematicity is essentially about a distribution of cognitive capacities–instances of an architecture (i.e., people at various points in development) associated with groups of cognitive capacities. Cognitive architecture may take on a variety of instantiations due to, say, genetic endowment, maturation, or experience (learning). Returning to the blocks example, suppose one cannot infer that the left block is blue given a (blue, yellow) pair of blocks, because the two colours interact to form a single colour, green, so that the constituent colours are lost and no longer retrievable. In this scenario, having the capacity to infer blue and yellow as the left blocks when paired with other coloured blocks does not extend to the (blue, yellow) pair. In this sense, we say cognition is quasi-systematic with respect to a particular domain, where quasi-systematicity is just a further refinement to a more specialized collection of systematic (intrinsically connected) capacities. That systematicity and quasi-systematicity are just differences in degrees of the same basic phenomenon motivates our proposal for a general theory explaining both.

Notice that the converse situation is also possible, albeit unlikely, where the “exceptional” cases are intrinsically linked, but the “unexceptional” cases are not, and we shall also illustrate this. Such cases dispel another misconception: that the argument from systematicity to cognitive architecture is a *fait accompli* for classical theory–that is, that systematicity is defined in a way that only classical theory can hope to explain (again, see [2] for a review). On the contrary, it is possible to construct a cognitive system that has the capacity for inferring, say, John as the lover from *John loves Mary* if and only if it has the capacity to infer *The ground is wet* from *Rain causes wet ground* and *It is raining*, simply by constructing an architecture that triggers both capacities in the presence of either case. Such “facts” would not be explained by the classical theory, since they don't share a common syntactic process. More to the point, there is no logical necessity even for capacities pertaining to *John loves Mary* and *Mary loves John* to be intrinsically connected given the possibility of having an architecture whereby each and every capacity is acquired by rote-learning. (Another meaning of quasi-systematicity, not adopted here, characterizes the degree of generalization exhibited by connectionist networks in language learning tasks [11].)

Logical possibilities aside, not just any group of capacities are intrinsically connected in regard to human cognition. Relevant to this point, and our theoretical motivation, is the observation that the groups of intrinsically connected capacities are related by “common structure” (i.e., informally, the relationships between constituent entities of interest). Syntactically structured representations and syntax-sensitive processes are *one* way of modeling structure. So, it behooves us to work from a *theory of structure*, rather than prescribe theoretical development via a particular model [4]. Category theory is a theory of structure, *par excellence*. With these considerations in mind, we proceed from definitions of the formal category theory concepts employed (Methods) to our general theory of (quasi-)systematicity and its application to specific cognitive domains (Results). In the final section (Discussion), we discuss the implications of our theory and how it can be tested. As a somewhat intuitive preview of the theory to come, the category theory construct central to our theory is the formal concept of an adjunction, being a particular kind of “universal construction”–a construction is universal in that it conveys all essential properties in the domain (category) of interest, and does it in a unique way. Thus, having the universal construction (capacity) is necessary and sufficient for having all other intrinsically connected constructions (capacities). In category theory terms, given a universal morphism, each and every morphism in the category factors through it. Thus, no further *ad hoc* assumptions are required, meeting the same explanatory standard for (quasi-)systematicity [1], [2], [4]. All systematic and quasi-systematic properties of human cognition are just instances of universal constructions of which adjunctions (also used to explain fully systematic properties [4]) are special cases.

## Methods

In this section, we introduce the category theory definitions used for our general theory of systematicity. Formal introductions to category theory are available from a variety of sources [7], [12]–[14]. Our introduction is necessarily brief. A table of notations is provided in Text S1, and more complete details of these formal concepts and their relationships are provided in Text S2 (see also [4], [15]). The concept central to our general theory of systematicity is adjunction, involving products and pullbacks as important cases. Adjunction depends on the concepts of category, functor, and natural transformation. So we proceed by first defining these concepts before defining adjunction. Adjunction, product and pullback are particular kinds of universal constructions. Universal construction and the unifying concept of comma category provide further perspective on our explanation for systematicity, so these concepts are also detailed in Text S2. (Some definitions have *duals*, obtained by reversing the directions of arrows in the original definitions, and their definitions are provided in Text S2.)

### Category

A *category* 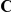 consists of a class of objects 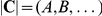; a set 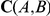 of morphisms (also called arrows, or maps) from 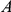 to 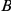 where each morphism 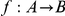 has 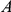 as its *domain* and 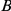 as its *codomain*, including the *identity* morphism 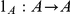 for each object 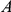; and a composition operation, denoted “
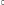
”, of morphisms 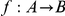 and 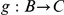, written 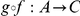 that satisfies the laws of:

• *identity*, where 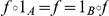, for all 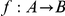; and
• *associativity*, where 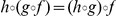, for all 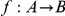, 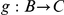 and 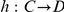.

One may think of a category as modeling a cognitive domain, where objects are sets of cognitive states, and morphisms are cognitive processes mapping possible cognitive state transitions. In this case, the category is 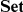, having sets for objects and functions for morphisms, where the identity morphism is the identity function sending elements to themselves and composition is the usual composition of functions. The structure of the domain is the morphisms. Our theory of systematicity is not specifically limited to 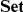, and can employ other categories as appropriate to the cognitive domain of interest. For example, the category 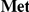 of metric spaces (objects) and continuous functions (morphisms) may be appropriate for cognitive domains concerning continuous instead of discrete entities. Furthermore, the objects themselves may house additional internal structure (relations between elements within the object), in which case the (homo)morphisms may be considered as structure-preserving maps, such as in the category 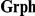 of graphs and graph homomorphisms (see, e.g., Text S3).

Here, we also define *isomorphism* for its use later in the paper. A morphism 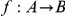 is an *isomorphism* if there exists a morphism 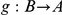, such that 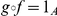 and 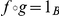. If 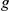 exists, then it is said to be the *inverse* of 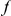, and it is also denoted 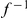. If 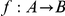 is an isomorphism, then 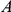 is said to be *isomorphic* to 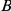, written 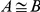.

### Functor

A *functor* 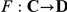 is a structure-preserving map from a domain category 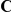 to a codomain category 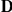 that sends each object 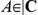 to an object 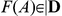; and each morphism 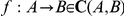 to a morphism 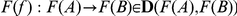, such that 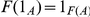 for each object 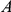; and 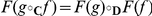 for all morphisms 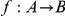 and 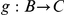 for which compositions 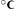 and 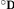 are defined in categories 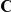 and 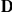, respectively.

Functors preserve structure in that every morphism in the domain category is associated with just one morphism in the codomain category, though this association does not have to be unique. Functors also provide a means for constructing new categories from old. In our context, one may think of functors as a means for constructing new cognitive representations and processes from existing ones. Thus, functors provide the formal starting point for a theory about the systematicity of cognitive capacities.

### Natural transformation

A *natural transformation* 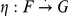 from a functor 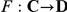 to a functor 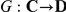 consists of 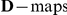 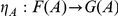 for each object 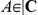, such that for every morphism 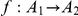 in 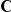 we have 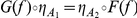, as indicated by the commutative diagram
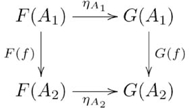
(1)


(A diagram is said to be *commutative* when any two compositions identified by paths with the same start object and the same finish object yield the same morphism, where at least one path has length greater than one.) For the diagram in Figure S1, commutativity means that 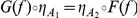.

A natural transformation is a *natural isomorphism*, or *natural equivalence* if and only if each 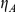 is an isomorphism. That is, for each 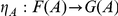 there exists a morphism 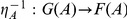 such that 
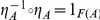
 and 
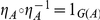
.

Natural transformations relate functors (see Text S2, for an example), which we use to model cognitive constructions. So for our purposes one may think of natural transformations as a way of relating cognitive constructions.

### Adjunction

An *adjunction* consists of a pair of functors 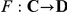, 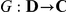 and a natural transformation 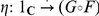, such that for every 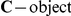 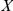, 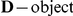 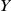, and 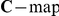 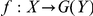, there exists a unique 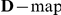 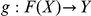, such that 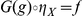, as indicated by the following commutative diagram:
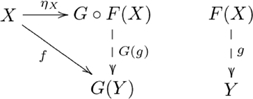
(2)


The two functors are called an *adjoint pair*, denoted 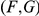, where 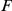 is the *left adjoint* of 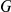 (written, 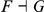), and 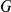 is the *right adjoint* of 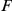, and 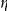 is the *unit* of the adjunction. (An equivalent definition of adjunction from the perspective of the *counit* is given in Text S2.)

A pair of adjoint functors may be thought of as reciprocating actions that are in some sense “conceptual” inverses of each other. By contrast, an isomorphic functor has an actual inverse. The composition of an isomorphic functor with its inverse sends objects and morphisms to themselves. The composition of right and left adjoints relates (co)domain objects and morphisms by a natural transformation, a relationship that is not necessarily an identity. Hence, adjunction is a more general concept than isomorphism: though every isomorphic functor has right and left adjoints (i.e., its inverse–e.g., 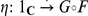 is the identity transformation, since 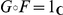), an adjoint functor is not necessarily an isomorphic functor (see Text S2). An example is given in Text S2, where the conceptual inverse of injection (left adjoint) is extraction (right adjoint); the related categories are not isomorphic, because the category resulting from the injection contains more objects and morphisms. The next section details adjunctions involving products and pullbacks used to address specific cases of systematicity. These adjunctions are also conceptual inverses in the sense that one functor takes wholes to produce copies as parts and the other functor composes combinations of parts back into new wholes. The categories in these adjoint situations are not isomorphic, because there are generally more part combinations than wholes.

#### Product

A *product* of two objects 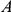 and 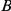 in category 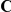 is, up to unique isomorphism, an object 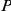 (also denoted 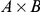) together with two morphisms (sometimes called *projections*) 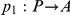 and 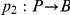, jointly expressed as 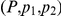, such that for every object 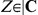 and pair of morphisms 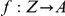 and 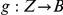 there exists a unique morphism 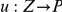, also denoted 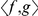, such that the following diagram commutes:
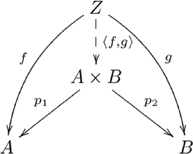
(3)


The Cartesian product of sets is a product in 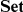 (though, some categories do not have products).

The *pair diagonal functor* 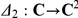 is specific to the category of pairs, 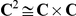, whose objects are pairs, 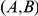, and morphisms are pairs of arrows, 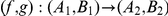, where 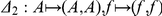.

The *product functor* 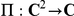 is also specific to the category of pairs, where the object component is 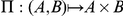, and for morphisms 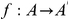, 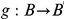, and 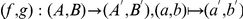, the morphism component is 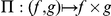.

This (diagonal, product) adjoint pair is indicated in commutative diagram
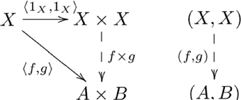
(4)


from the perspective of the unit of the adjunction, 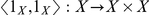, and commutative diagram
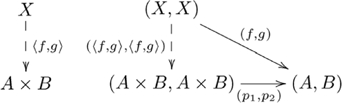
(5)


from the perspective of the counit, 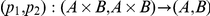.

#### Pullback

A *pullback* of two morphisms 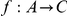 and 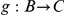 in category 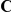 is, up to a unique isomorphism, an object 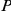 (also denoted 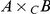) together with two morphisms 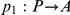 and 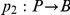, jointly expressed as 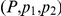, such that for every object 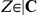 and pair of morphisms 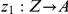 and 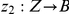 there exists a unique morphism 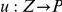, also denoted 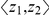, such that the following diagram commutes:
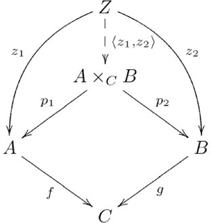
(6)


Objects 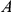, 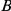, and 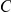, and morphisms 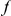 and 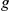 correspond to the shape of these compositions, called a *sink* (see Text S2). A pullback may be thought of as a product of objects 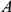 and 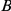 constrained at 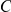. In the category 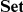, for example, 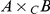 is, up to unique isomorphism, the subset of the Cartesian product 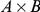 that includes just those pairs of elements 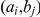 satisfying the constraint that 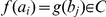. (Not all categories have pullbacks.) With this intuition in mind, we can begin to see how pullbacks pertain to quasi-systematicity of binary relations, which we address next. The associated (diagonal, pullback) adjoint pair is given in Text S2.

## Results

Having provided the basic category theory definitions, we now proceed to show how (quasi-)systematicity follows from adjunctions without recourse to *ad hoc* assumptions. The indivisible nature of systematically related capacities is made explict from the perspective of the more general concept of universal construction (see Text S2, for a definition). Hence, we also re*view* our explanation for systematicity of (binary) relations and relational schemas [4] from the perspective of universal constructions, and show that this perspective extends to quasi-systematicity.

### Systematicity: Natural relations

Systematicity of relational propositions was explained by a (diagonal, product) adjoint functor pair [4]. The example domain was a group of inferences that included instances such as the capacity to infer *John* as the lover from proposition *John loves Mary*, *Mary* as the lover from *Mary loves John*, and so on. The explanation involves the (diagonal, product) adjoint, where the left adjoint is the pair diagonal functor 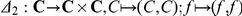, and the right adjoint is the product functor 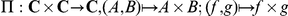, and the adjunction is indicated by the following commutative diagram:
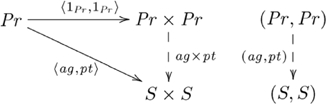
(7)


where object 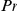 is the set of *loves* propositions, 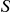 is the set of agents/patients (e.g., *John*, *Mary*), and morphisms 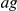 and 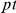 extract the agent and patient (respectively) from a proposition.

Given a cognitive capacity for a relation realized as a particular product, the commutativity property of the adjunction ensures that there is one and only one way to realize the other capacities, obviating the need for an *ad hoc* assumption dictating a specific product. An explanation based solely on products, or product constructing functors, has the same sort of problem as one based solely on classical compositionality, as we are about to show. For an architecture with all products having the same form, in general say, 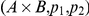, where 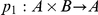 and 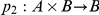 are the projections, the capacity to infer John as the lover in *John loves Mary* by 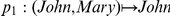 extends to the capacity to infer *Mary* as the lover in *Mary loves John* since 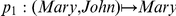. However, 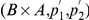 is also a product of 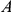 and 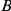, where 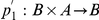 and 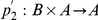. So, an architecture can also be constructed that has the capacity to correctly infer *John* as the lover in *John loves Mary* from 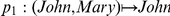, by employing 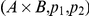, while also having the capacity to incorrectly infer *John* as the lover in *Mary loves John*, by employing 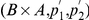, because 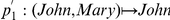. Hence, for an explanation based solely on products, an *ad hoc* assumption is required to exclude this second type of architecture, just as such assumptions are required for the classical explanation (cf. classical architectures based on grammar G1 versus G2 in [4]).

If we base our explanation of systematicity on a product specifically obtained via an adjunction, however, then the commutativity property of the adjunction rules out the second architecture. That is, given a capacity realized by a product of one form, say, 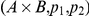, then only the product functor 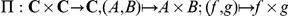 (implicit in Figure S7) makes this diagram commute. Product functor 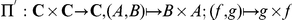 does not, since 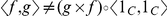 [4]. Commutativity ensures that all other capacities are realized systematically.

The commutativity property of an adjunction enforces a particular (cognitive) construction that is universal (see Text S2) in the category (cognitive domain) of interest. Cognitive capacities are indivisibly linked via a common, *mediating* arrow. This mediating arrow is made explicit from the perspective of universal construction, and explains the indivisible nature of certain groups of cognitive capacities. This adjoint situation is indicated in the following commutative diagram:
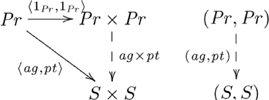
(8)


where 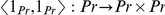 is the unit of the adjunction. The adjunction is also given in diagram
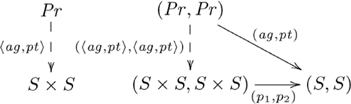
(9)


where the counit 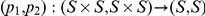 is a mediating arrow. (The counit of the adjunction is also a mediating arrow.)

As a universal construction, the explanation for systematicity is rendered more explicitly in Figure S9. From this diagram we see that the capacity to infer the lover (agent), or one being loved (patient) from a proposition such as *John loves Mary*, i.e., an arrow 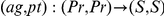, has two components: the arrow 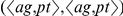, which is guaranteed to exist uniquely, by the commutativity property of the adjunction; and the mediating arrow 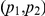, which is common to all such capacities. Thus, it is the presence or absence of the mediating arrow that implies the presence or absence of all capacities pertaining to the *loves* propositions. Hence, this universal construction provides an explanation for systematicity of relations.

An alternative view of our explanation for systematicity is provided by the equivalent *hom-set* definition of adjunction (see Text S2). From this definition, we obtain the following diagram showing how the objects and morphisms in the two categories are related by the adjoint functors:
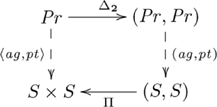
(10)


This view highlights both the constructive nature of functors, and how a particular unique relationship between constructions is enforced by an adjunction. It also highlights the informal notion of an adjunction as a correspondence between processes, as (in general) each dashed arrow indicates a set of possibly several morphisms, and there is a one-to-one correspondence between the two sets. Adjunctions are also unique up to unique isomorphism. The unique existence of alternative construction 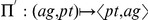 is enforced by the commutativity property of the 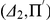 adjoint, where the correspondence is indicated in the following diagram:
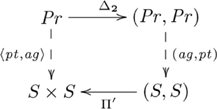
(11)


The explanation for the systematicity of relational schemas [4] is also a special case of our general theory, albeit employing a different category. The details of this explanation are provided in Text S3.

### Quasi-systematicity: Natural relations

Our explanation for systematicity in terms of adjunctions (universal constructions) also extends to quasi-systematicity of relational inference (i.e., for relations that do not extend to all possible combinations of elements) via pullbacks. We provide several examples involving different kinds of relations, and an explanation in terms of an adjunction employing a particular kind of pullback for each case.

Note that our explanation for quasi-systematicity of relations involving pullbacks subsumes our explanation for full systematicity involving products. Recall that a pullback 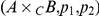 and the morphisms 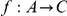 and 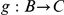 (see Figure S6) can be thought of as a “product” of 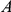 and 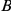 constrained by object 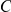 and the morphisms 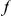 and 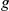, such that 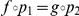. In the case that 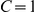 (i.e., a terminal object, see Text S2), then 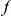 is the unique morphism 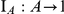 and 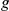 is the unique morphism 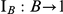, which are guaranteed to exist (by definition of terminal). In effect, 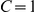 provides no constraint on the product. Thus, 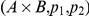 is a special case of 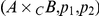, i.e., 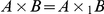, and so our general explanation in terms of pullbacks subsumes our special explanation in terms of products.

We use the relation *parent* (e.g., *mares parent colts*) to illustrate our explanation of quasi-systematicity in terms of pullbacks. If one knows that *mares parent colts* and *stallions parent fillies* then one also knows that *mares parent fillies* and *stallions parent colts*. Likewise, if one knows that *cows parent steers* and *bulls parent heifers*, then one also knows that *cows parent heifers* and *bulls parent steers*. Yet, one would not also think that *mares parent steers*, or *bulls parent fillies*. One also would not think that *colts parent stallions*, or *heifers parent bulls*. An architecture based only on a product is inadequate. Instead, the quasi-systematic capacities associated with this relation derive from a pullback.

The pullback diagram associated with the *parent* relation is an instantiation of the diagram in Figure S6. In particular, the pullback for this relation is given in the following commutative diagram:
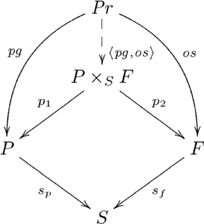
(12)


where 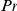 is the set of valid propositions, with 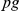 and 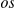 as the progenitor and offspring maps (respectively), 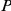 is the set of progenitors, 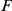 is the set of offspring, 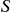 is the set of species, 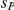 and 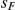 map the progenitors and offspring to their species (respectively), and 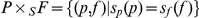. Suppose 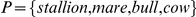, 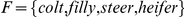, and 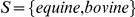, so that 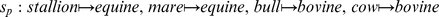, and 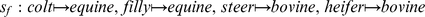. Therefore, 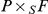 is the set {(*stallion*, *colt*), (*stallion*, *filly*), (*mare*, *colt*), (*mare*, *filly*), (*bull*, *steer*), (*bull*, *heifer*), (*cow*, *steer*), (*cow*, *heifer*)}, which contains just the elements of the relation.

**Figure 1:**
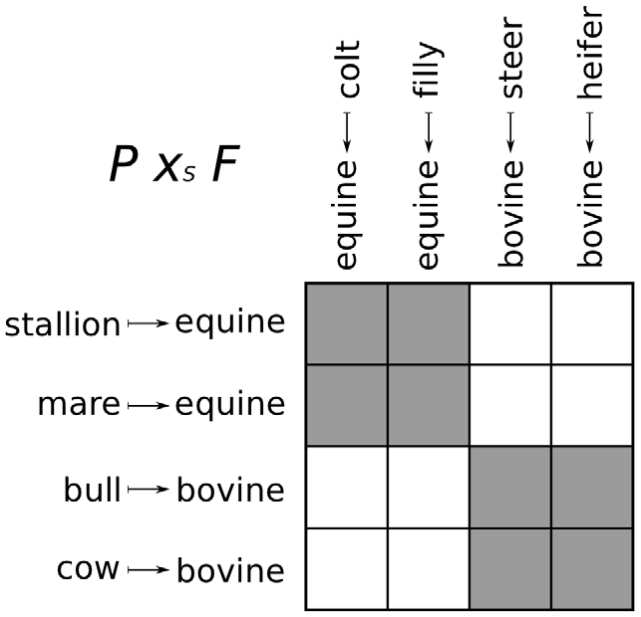
Block matrix representation of *parent* pullback.

In 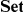, the elements of a pullback object 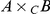 can be visualized as the main diagonal of a block matrix, whose row and column labels are the values of 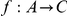 and 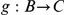 for each 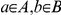, where the blocks contain the cells, 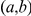, with common row and column labels, corresponding to 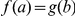. For example, the block matrix representation of the *parent* pullback is shown in Figure 1.

The adjoint for this example is 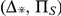, where 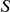 is the constraining object, and 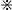 refers to constructs that are specific to pullbacks (see Text S2). The adjunction is indicated by the following diagram:
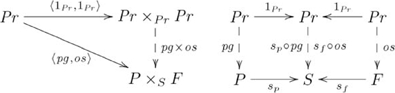
(13)


where 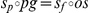 and 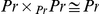, i.e., in general, the pullback 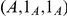 of 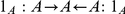 has the universal property of pullbacks, which is easy to show. The diagram in Figure S13 simplifies to:
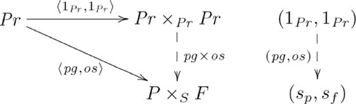
(14)


where a composite such as 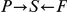 in Figure S13 is identified by the morphisms (i.e., 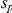 and 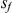, mapping parents and offspring to species), and a map between such composites by the corresponding morphisms between the *outer* objects. For example, 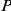 and 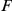 are the outer objects in 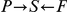, and 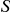 is the *inner* object. Reference to the morphism between inner objects is omitted, because it is determined by the other morphisms.

The explanation for quasi-systematicity comprises two parts: one part pertains to the constraints on allowable elements; and the other part pertains to universal construction, and is essentially the same explanation as that for full systematicity, except that the universal construction is associated with pullbacks.

Regarding the constraints part of the explanation, there are two sources of constraints in the form of the sets containing the possibly related elements (i.e. 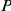 and 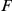 in this example), and the requirement that the diagam in Figure S12 commutes. That 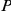 contains only progenitors and 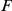 only offspring precludes pairs corresponding to *colts parent mares*, for example. The fact that the diagram in Figure S12 must commute (to be a pullback) precludes instances corresponding to *stallions parent steers*, for example, because stallion and steer belong to different species.

The universal construction part of the explanation parallels the explanation for full systematicity, starting with the following commutative diagrams:
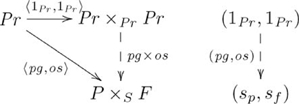
(15)


and
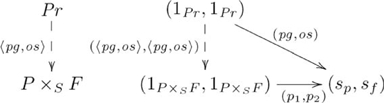
(16)


where mediating arrow 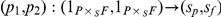 is the counit, and 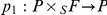 and 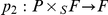 (see also Text S2). To paraphrase, given a cognitive capacity for a relation realized as a particular pullback, then the commutativity property of the adjunction ensures that there is one and only one way to realize the other capacities, obviating the need for an *ad hoc* assumption stipulating which pullback. In particular, 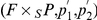, where 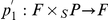 and 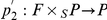, is also a pullback. Thus, from pullbacks alone an architecture can be constructed whereby mare is correctly inferred as the progenitor in *mares parent colts* by 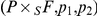 and 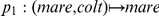, but *steer* is incorrectly inferred as the progenitor in *bulls parent steers* since 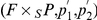 and 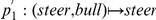. The commutativity property of the adjunction rules out an architecture that mixes different possible pullbacks. As with full systematicity, quasi-systematic capacities are indivisibly linked by a universal arrow, i.e., 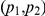.

This form of pullback is sufficient when the capacity subgroups (one subgroup per species, in this example) are themselves locally, fully systematic. In some situations, this condition may not hold. For example, suppose we introduce *whale* and *calf* as additional progenitor and offspring elements, respectively. By associating *whale* and *calf* with *mammal*, the pullback above would yield (*whale*, *calf*), but also (*whale*, *steer*), and (*whale*, *heifer*) where these elements where also associated with *mammal*. Clearly, the term *calf* is being used in two senses that need to be distinguished. One sense pertains just to cattle, and the broader sense includes large mammals, such as elephants and seals as the parents of calves. These subgroups are distinguished by using another pullback that incorporates this additional structural information.

The pullback in this new situation is indicated in the following diagram:
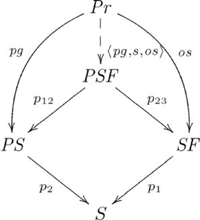
(17)


where 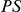 contains the parent-species pairs, 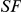 contains the species-offspring pairs, and the constraining object 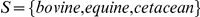 contains the new species information distinguishing the senses of *calf*. Morphisms 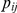 project out the 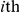 and 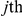 elements of each triple (respectively), and 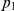 and 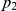 are the usual projections, picking out the first and second elements of each pair (respectively). The pullback of these two morphisms is 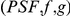, where 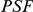 is the set of triples containing instances such as (*whale*, *cetacean*, *calf*), corresponding to *whales parent calves*, but no instance that includes both *whale* and *steer*, etc. The two senses of *calf* are captured by pairing *bovine* with *calf* for one sense and *cetacean* with *calf* for the other in 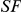, and *bull* and *cow* with *bovine*, and *whale* with *cetacean* in 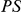. Since *bull*, *cow*, *steer* and *heifer*, etc. are not paired with *cetacean*, instances corresponding to *whales parent steers* are not contained within the collection of quasi-systematic capacities.

### Quasi-systematicity: Formal relations

Quasi-systematicity also occurs with formal (mathematical) relations, such as the function *square*, i.e., 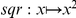, treated as a relation between whole numbers, i.e., the set 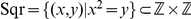, where 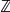 is the set of integers. There are two (quasi-)systematic aspects to this relation: The first one is illustrated as follows: if one has the capacity to infer that 9 (3) is the square (root) from “3 square 9”, then one also has the capacity to infer that 16 (4) is the square (root) from “4 square 16”, but one would not infer that 4 (16) is the square (root) of 16 (4). The second aspect is also illustrated: if one has the capacity to know that “3 square 9” and “-3 square 9”, then one has the capacity to know that “4 square 16” and “-4 square 16”. We address each aspect in turn.

The quasi-systematic nature of *square*–the fact that it does not include all possible pairs in 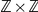
–is indicated by the pullback in the following diagram:
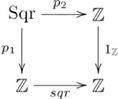
(18)


where the pullback object 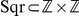 is at the top-left corner. Here, and further on, we omit the other object and morphisms, corresponding to 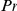, 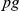, 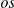 and 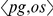 in Figure S17. As a universal construction, 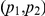 is a mediating arrow, and the adjunction is the (diagonal, limit) adjoint specific to pullbacks. Having provided a pullback and hence an adjoint, the explanation for quasi-systematicity proceeds as before. The same situation applies to the remaining examples, so we only provide the universal construction (most often a pullback) in those cases too.

The second systematic aspect results from a *coequalizer* (see Text S2), such that 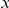 and 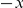 are equivalent, under the relation *absolute*. With respect to this relation, 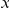 and 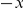 are the “same” and constitute an *equivalence class* (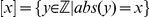, where 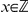 and 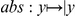, and the set of such equivalence classes is a *quotient set* (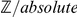). A formal definition of equivalence in terms of category theory constructions is given in Text S4. A coequalizer is a *colimit*, which is also a universal construction arising from an adjunction (see Text S2), hence the explanation for this second aspect of systematicity parallels the other explanations. A coequalizer is equivalent to a particular kind of *pushout* (see Text S4; dually, an *equalizer* is equivalent to a particular kind of pullback [7]). The *absolute* equivalence relation, denoted 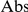, is given in the following diagram expressing a pushout:
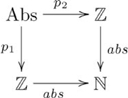
(19)


where 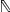 is the set of natural numbers including 0. The pushout is 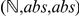, and 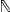 is isomorphic to the quotient set 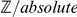 associated with this equivalence relation.

Since equivalence classes are familiar to cognitive scientists, one may wonder why more elaborate category theory concepts are necessary. The answer is that the category theory approach is necessary to establish that these constructs (with products, pullbacks, and coequalizers) are unique, not *ad hoc*. We also point out (in Text S4) that this example of equivalence in terms of a coequalizer also provides a formal category theory *definition* of systematicity.

Another example of quasi-systematicity with respect to a formal relation is *less-than*, i.e. the set 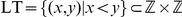. We detail this example because it introduces a general kind of pullback that addresses quasi-systematicity for an arbitrary relation. As with the *square* relation, if one has the capacity to infer that 2 is the lesser number from “2 is less than 3”, then one also has the capacity to infer that 4 is the lesser number from “4 is less than 5”, but one would not infer that 5 is the lesser number. The structural difference between *less-than* and *square* relations is that *less-than* is not a function, and so the previous pullback cannot be used. Instead, quasi-systematicity for *less-than* is explained using the pullback indicated in the following diagram:
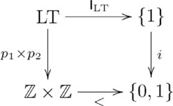
(20)


where 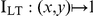, for all 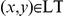, 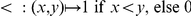, and 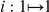 is an inclusion. Again, the relation is captured in terms of a pullback and the explanation for quasi-systematicity proceeds as before.

In general, this kind of pullback can be employed for an arbitrary relation 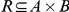. Category theorists will recognize the diagram in Figure S20 as similar to a *subobject classifier*, a critical part of a definition of a *topos*–*topos theory* is a branch of category theory that, among other things, provides a category-theory basis for logic (see, for example, [16] for an introduction). Hence, topoi are likely to be involved where an explanation for systematicity is required for logic-related cognitive behaviours, though we do not pursue this topic further here.

### Quasi-systematicity in language

Language is another domain where quasi-systematicity is evident, as mentioned in the Introduction. Here, we present two examples. Other situations involving further category theory concepts are discussed in the final section (Discussion).

Our first example is subject-verb agreement: for English speakers, agreement between the subject and verb means that the capacity for *the dogs chase the cats* and *the dog chases the cats* implies the capacity for *the cats chase the dogs*, but not *the cats chases the dogs*, nor *the cat chase the dogs*, etc. The present example is confined to third-person agreement, though the explanation extends to first- and second-person. Subject-verb agreement is enforced by a pullback indicated in the following diagram:
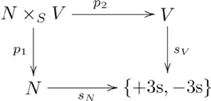
(21)


where 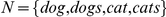 is a set of nouns, 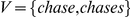 is a set of verbs, 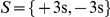 is the set of attributes, and 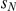 and 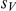 are the morphisms mapping nouns and verbs to their singularity attribute (respectively), indicated as 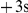 (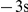) meaning is (not) third-person singular. The pullback object 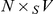 contains the quasi-systematic relationships, e.g., (*dogs*, *chase*) and (*cat*, *chases*), but not (*dogs*, *chases*). Hence, quasi-systematicity with respect to this domain is explained by an adjunction involving this pullback.

Some nouns, such as *sheep*, are both singular and plural. In this case, we need a pullback similar to the one used in the extended *parent* relation (see Figure S17) that captures this additional structural information. The corresponding pullback is indicated as follows:
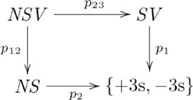
(22)


where 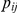 and 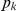 are the usual projections. In the case of *sheep*, 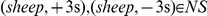, so that the pullback object 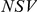 contains 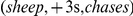 and 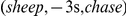, e.g., *the sheep chases the farmer* and *the sheep chase the farmer*, respectively.

Our second linguistic example involves the difference between verbs *drench* and *throw*: English speakers say *I drenched the flowers with water*, but not *I drenched water onto the flowers*, whereas they say *I threw water onto the flowers*, but not *I threw the flowers with water* [10]. Whether or not the verb requires a preposition such as *onto*, or *over* is considered to depend on whether or not the meaning of the verb specifies how the water got onto the flowers [10]. Verbs that require *onto* include: *dripped*, *threw*, *poured*, and *tossed*. Verbs that require no preposition include: *dampened*, *drenched*, and *wet*. The pullback for this situation is similar to the previous one, and indicated in the following diagram:
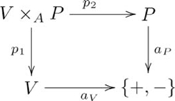
(23)


where 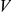 is the set of verbs, 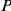 the set of prepositions {onto, over, 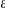}, where 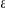 indicates no preposition, 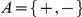 is the set of attributes, and 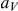 and 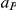 are the morphisms mapping verbs and prepositions to their preposition attribute (respectively), indicated as 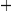 (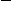) meaning does (not) require a preposition.

## Discussion

A fundamental question for cognitive science concerns the systematic nature of human cognition–why does having certain cognitive capacities imply having certain others? An answer to this question speaks to the nature of human cognitive architecture–the basic processes and modes of composition that together bring about cognitive behaviour. In so far as cognition is systematic, our category-theoretic answer says that systematicity is a necessary consequence of a (categorial) cognitive architecture whose basic processes are functors that participate in adjunctions. Thus, on this basis, adjoint functors constitute building blocks of human cognition.

There is common ground between our category theory explanation and the classical compositionality one. Both theories assume complex representations and processes that are built out of simpler ones, and some category theory constructions generalize classical ones, as mentioned elsewhere [4]. So, a classical theory of systematicity may be compatible with our categorial one.

Nonetheless, the quintessential difference between the two theories is the adjunction, which accounts for systematicity without having to stipulate a specific correspondence between processes for constructing representations and processes for accessing components of those constructions. Alan Turing is credited with providing a key advance concerning the foundations of cognitive science, overcoming the problems with associativism by suggesting that cognitive processes are instead (syntactic) computations [17]. Turing's (classical) solution works well for computational systems, because the correspondence between the processes for constructing compositional representations of complex entities and the processes for accessing the representational components corresponding to their constituents is systematically maintained by the designer of the system. However, a theory of cognitive systems demands an explanation for such correspondences just in terms of the system and its interaction with the world, not some third party. Our explanation meets this criterion, where the correspondence is enforced by the commutativity property of the adjunction.

This conception of adjunction as a building block of cognition is unique to our theory, and goes significantly beyond the widespread use of isomorphism (cf. analogy models) in cognitive science generally. A contrast of adjunction versus isomorphism highlights our shift in perspective: a reconception of cognitive architecture in terms of the relationships between structure-sensitive processes, instead of the representations that those processes transform (see also [4]). Other approaches to cognition, including classical ones typically treat representation in terms of an isomorphism between the representations and the entities those representations are intended to denote. From the category theory perspective, isomorphic domains modelled as categories are the same apart from a change of labels. An adjunction is more general, and potentially more useful, because two domains (involving quite different sorts of processes) that are not isomorphic, may still be systematically related by an adjunction, thereby affording an explanation that is not limited to cases whose domains are only superficially dissimilar.

The choice of adjunction will depend on the structure of the cognitive domain of interest. Most of our examples involved two specializations: one involving products, and the other pullbacks. These adjunctions differ in the *shape* of their composition: products involve pairs of objects, whereas pullbacks involve a third object and associated morphisms pertaining to the interaction between the other two. Thus, product-based adjunctions provide a natural explanation for full systematicity where there is no restriction on the group of intrinsically connected capacities beyond the type of constituents, whereas pullback-based adjunctions provide a natural explanation for quasi-systematicity, where the interaction at the third object accounts for the more refined group of intrinsically connected capacities.

Of course, our theory is not limited to just these kinds of adjunctions. For the most part, we have confined ourselves to the category 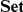. Category theory provides many other kinds of categories for a wide variety of applications for mathematics and other fields, such as computer science [18], linguistics [19], and physics [20]. In particular, the category of *pregroups* with certain morphisms interpreted as grammar rules has been used for a hybrid distributive-symbolic model of grammar [21]. An adjunction involving this or related categories may provide explanations for other quasi-systematic aspects of language (without *ad hoc* assumptions), that we have not addressed. The reader may have noticed that categories themselves are objects of other categories, e.g., 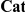 is the category with categories for objects and functors for morphisms. 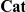 also has pullbacks (see [7], p74), which may be applicable to explanations of quasi-systematicity in other more complex cognitive domains.

Up to this point, we have referred to universal constructions and adjunctions synonymously with respect to our explanation of systematicity. This is despite the fact that the two constructs are technically different: in an adjoint situation every object in the respective category is a (co)free object, where a universal construction is associated with just one (co)free object (see Text S2). That is, an adjoint situation involves a collection of universal constructions. The difference in regard to our explanation of systematicity only concerns scope. For example, when our interest concerns a single (*loves*) relation, systematicity is explained in terms of a (co)free object, i.e., a product on the pair of objects constituting the relation. When our interests concern all relations pertaining to a particular cognitive domain (modelled implicitly as different pairs, or explicitly with a relation symbol, see [4], Diagram 17), systematicity is explained in terms of an adjunction, which includes all corresponding (co)free objects.

If adjunction is one of the basic components of human cognition, then what is its corresponding neural realization? An adjunction involves a reciprocal relationship between two functors, though the functors may not be inverses of each other. One possible approach to investigating neural correspondences, then, is with the reciprocal relationships between brain regions (see [22] for a category-theoretic integration of neural and cognitive levels, [23] for a category theory approach to modeling hippocampal place cells using colimits, and [24] for a category theory approach to designing neural networks).

Our theory can be tested with tasks that involve adjoint relationships between domains. Tasks involving isomorphic relationships between their instances are candidates. The trigram prediction task is such a task (see Text S3, and [4]). From our category theory perspective we see how this sort of paradigm can be extended to involve transfer between task instances that are more generally homomorphic, not just isomorphic. (Recall that an isomorphism is a (homo)morphism that is invertible, and so not many-to-one.) For example, letter sequences conforming to a particular grammar are modeled as a *free category* on the directed graph defining the grammar [25]. The transfer to a new set of sequences involving a new grammar may be homomorphic, where there is a many-to-one mapping from the elements in the previous grammar to elements in the new one. Yet another alternative, for testing the generality of the theory, includes tasks best modeled in categories other than 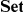, where the stimuli are continuous rather than discrete.

We close with some discussion on the relationship between systematicity and development/learning, both as a possible point of contact with another central concern of cognitive science (i.e., development and learning), and as a portent for future development. The systematicity problem is concerned with why does having certain cognitive capacities imply having certain others, whereas the broader development/learning problem is concerned with why those capacities are available in the first place. In our case, the broader question pertains to the origins of adjunctions and the constraints that determine object types and, in the case of pullbacks, morphisms pertaining to the constraint object. We note that a universal construction, and an adjunction (particularly) are a kind of optimal solution to a problem. A universal construction is one from which all other constructions are composed. In an adjoint situation, the left adjoint may be considered as the most efficient solution to the problem posed by the right adjoint. Or, conversely, the right adjoint is the most difficult problem that the left adjoint solves. In our context, that is the problem of systematically representing and making inferences about the world. Thus, learning and development may be treated, ideally, as a process of acquiring universal cognitive constructions, and in particular, adjoint cognitive processes. Such theoretical and empirical possibilities await further development of our category theory approach to cognition.

## Supporting Information

Figure S1 Natural transformation.
(TIF)

Figure S2 Adjunction.
(TIF)

Figure S3 Product.
(TIF)

Figure S4 Diagonal-product adjoint (unit).
(TIF)

Figure S5 Diagonal-product adjoint (counit).
(TIF)

Figure S6 Pullback.
(TIF)

Figure S7 Diagonal-product adjoint for *loves* relation.
(TIF)

Figure S8 Diagonal-product adjoint for *loves* relation (unit).
(TIF)

Figure S9 Diagonal-product adjoint for *loves* relation (counit).
(TIF)

Figure S10 Diagonal-product adjoint (hom-set view).
(TIF)

Figure S11 Diagonal-(alternative) product adjoint (hom-set view).
(TIF)

Figure S12 Pullback for *parent* relation.
(TIF)

Figure S13 Diagonal-pullback adjoint for *parent* relation.
(TIF)

Figure S14 Diagonal-pullback adjoint for *parent* relation (simplified).
(TIF)

Figure S15 Diagonal-pullback adjoint for *parent* relation (unit).
(TIF)

Figure S16 Diagonal-pullback adjoint for *parent* relation (counit).
(TIF)

Figure S17 Diagonal-pullback adjoint for extended *parent* relation.
(TIF)

Figure S18 Pullback for *square* relation.
(TIF)

Figure S19 Pullback for *absolute* relation.
(TIF)

Figure S20 Pullback for *less-than* relation.
(TIF)

Figure S21 Pullback for subject-verb agreement.
(TIF)

Figure S22 Pullback for extended subject-verb agreement.
(TIF)

Figure S23 Pullback for prepositions.
(TIF)

Text S1 Definitions of category theory notations used in the main text.
(PDF)

Text S2 Further explanation of the category theory concepts used to explain systematicity, and how these concepts are related to each other, their duals, more general forms, and to the unifying concept of comma category.
(PDF)

Text S3 An explanation for systematicity in “relational schema induction” using adjunction.
(PDF)

Text S4 A category theory definition of systematicity.
(PDF)
