# Supplementary figures and images for: Categorial Compositionality II: Universal Constructions and a General Theory of (Quasi-)Systematicity in Human Cognition
Source: PLoS Comput Biol. 2011 Aug 4;7(8):e1002102. doi: 10.1371/journal.pcbi.1002102 (PMC3154512; doi:10.1371/journal.pcbi.1002102)

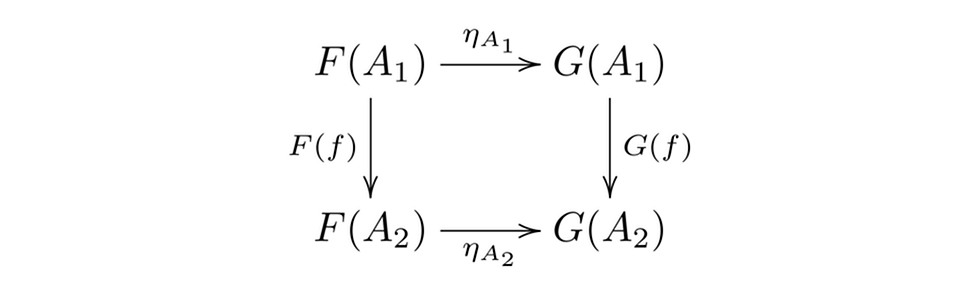

Supplement: Figure S1 — Natural transformation. (TIF) [file pcbi.1002102.s001.tif]

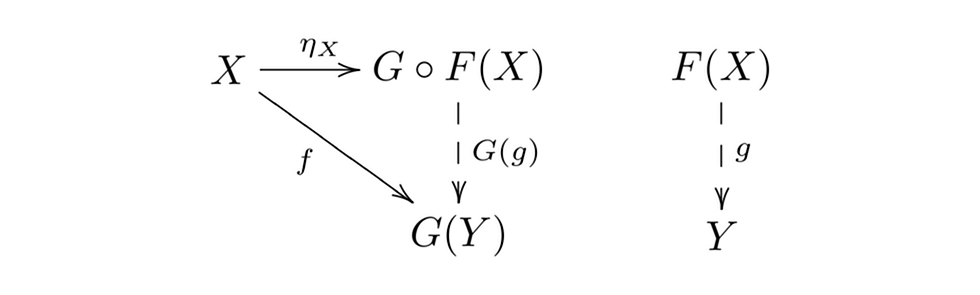

Supplement: Figure S2 — Adjunction. (TIF) [file pcbi.1002102.s002.tif]

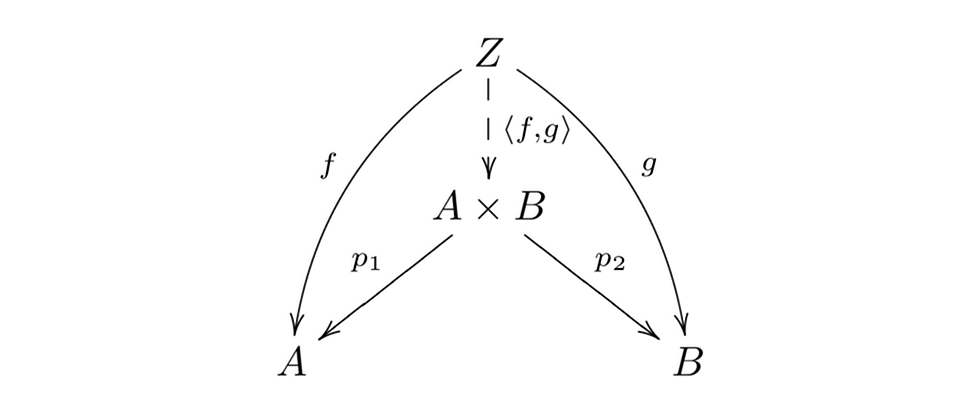

Supplement: Figure S3 — Product. (TIF) [file pcbi.1002102.s003.tif]

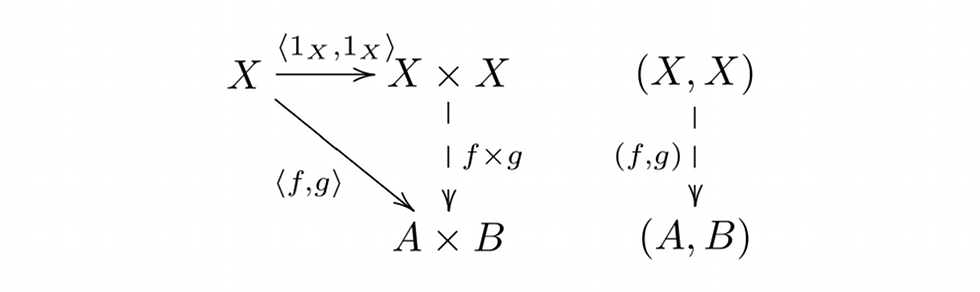

Supplement: Figure S4 — Diagonal-product adjoint (unit). (TIF) [file pcbi.1002102.s004.tif]

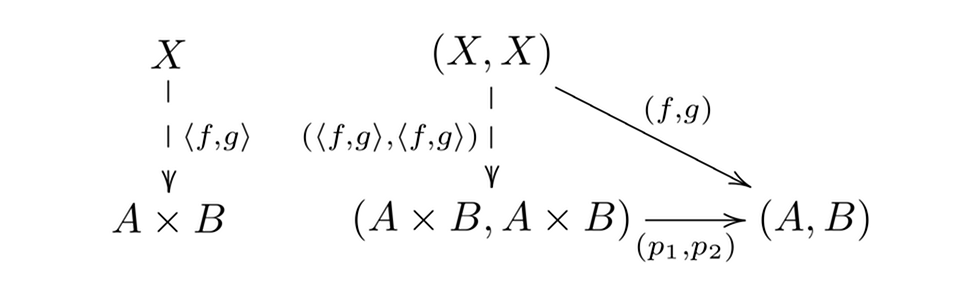

Supplement: Figure S5 — Diagonal-product adjoint (counit). (TIF) [file pcbi.1002102.s005.tif]

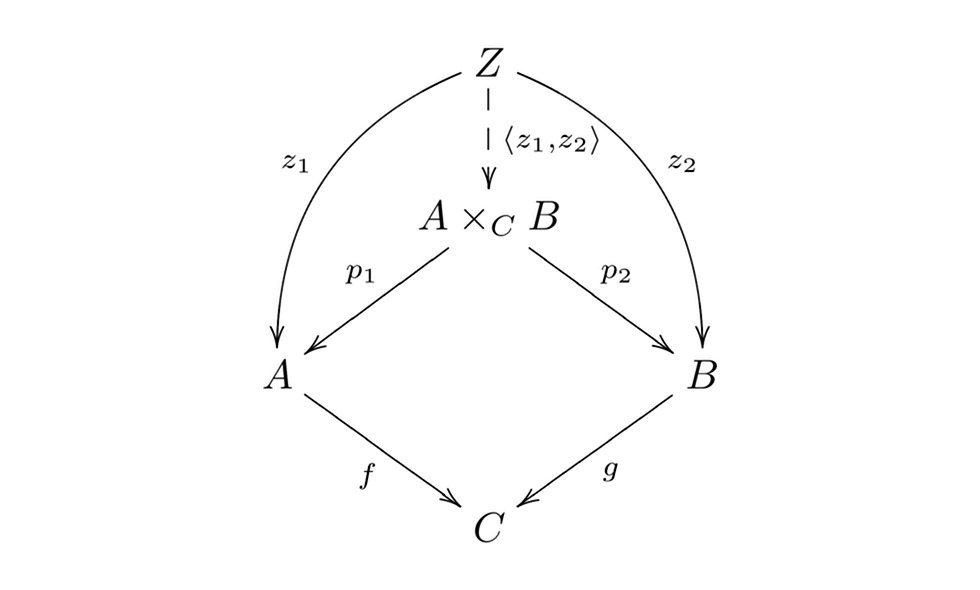

Supplement: Figure S6 — Pullback. (TIF) [file pcbi.1002102.s006.tif]

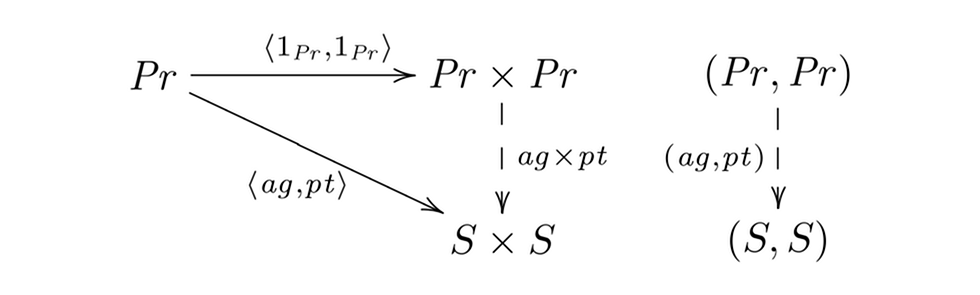

Supplement: Figure S7 — Diagonal-product adjoint for loves relation. (TIF) [file pcbi.1002102.s007.tif]

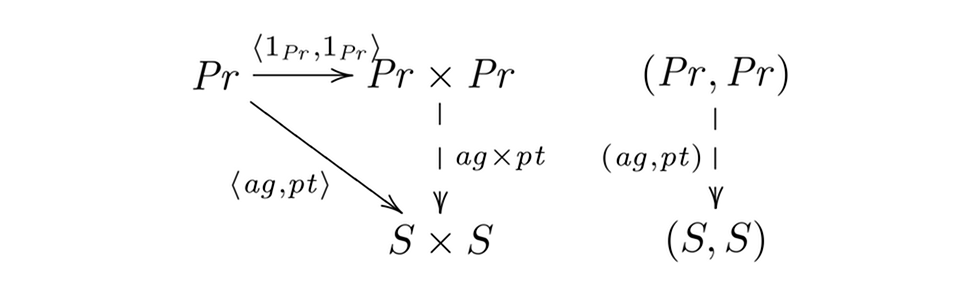

Supplement: Figure S8 — Diagonal-product adjoint for loves relation (unit). (TIF) [file pcbi.1002102.s008.tif]

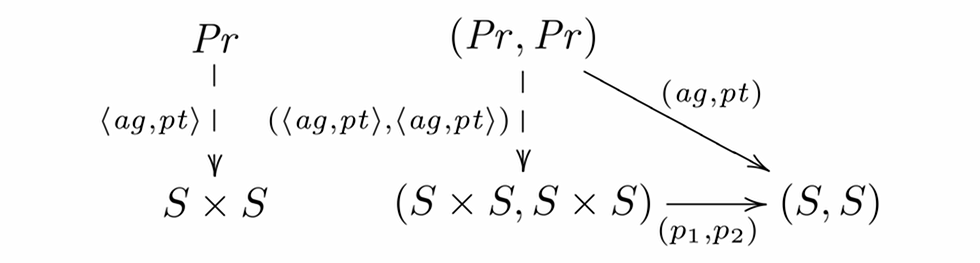

Supplement: Figure S9 — Diagonal-product adjoint for loves relation (counit). (TIF) [file pcbi.1002102.s009.tif]

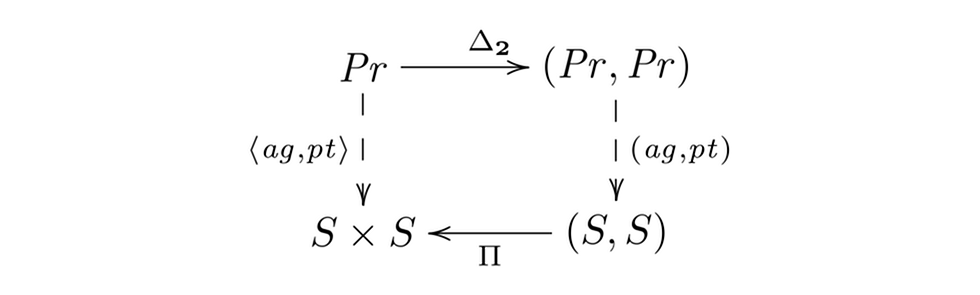

Supplement: Figure S10 — Diagonal-product adjoint (hom-set view). (TIF) [file pcbi.1002102.s010.tif]

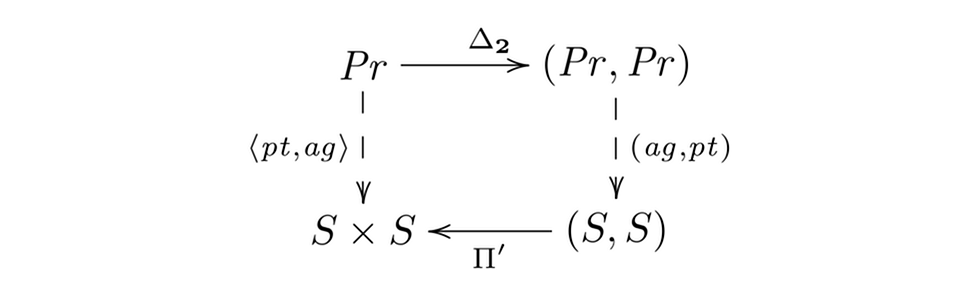

Supplement: Figure S11 — Diagonal-(alternative) product adjoint (hom-set view). (TIF) [file pcbi.1002102.s011.tif]

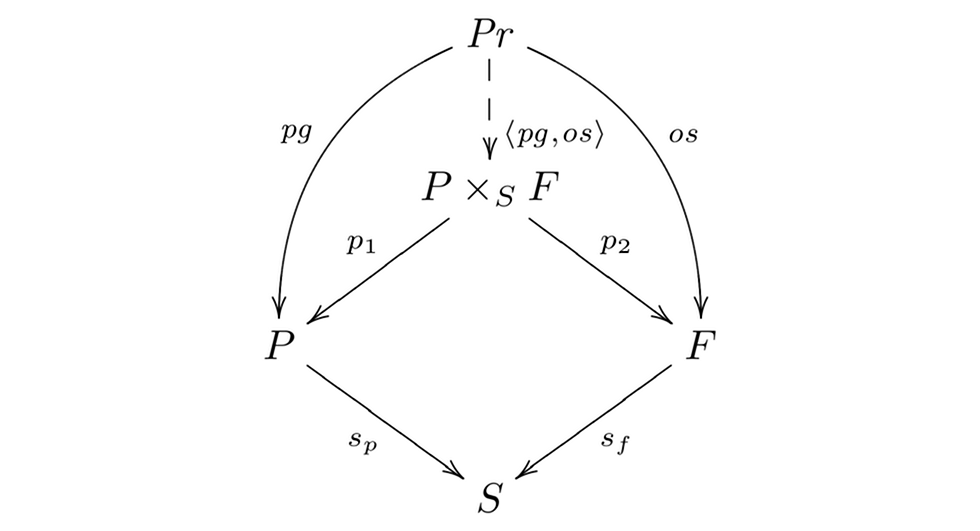

Supplement: Figure S12 — Pullback for parent relation. (TIF) [file pcbi.1002102.s012.tif]

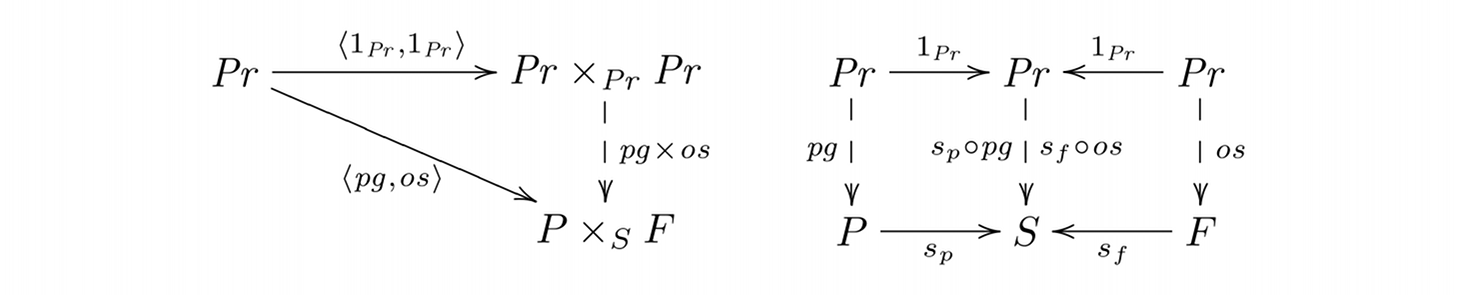

Supplement: Figure S13 — Diagonal-pullback adjoint for parent relation. (TIF) [file pcbi.1002102.s013.tif]

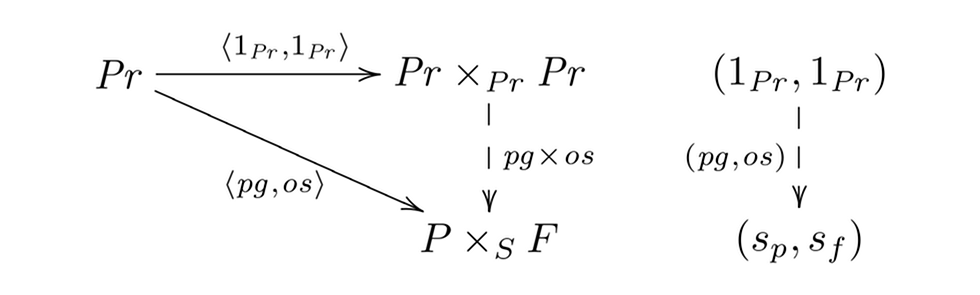

Supplement: Figure S14 — Diagonal-pullback adjoint for parent relation (simplified). (TIF) [file pcbi.1002102.s014.tif]

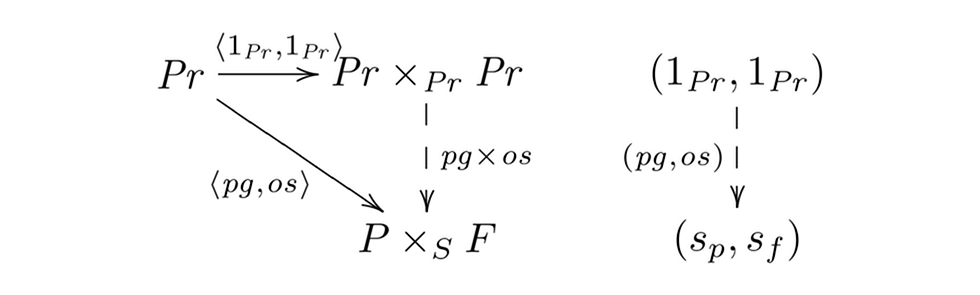

Supplement: Figure S15 — Diagonal-pullback adjoint for parent relation (unit). (TIF) [file pcbi.1002102.s015.tif]

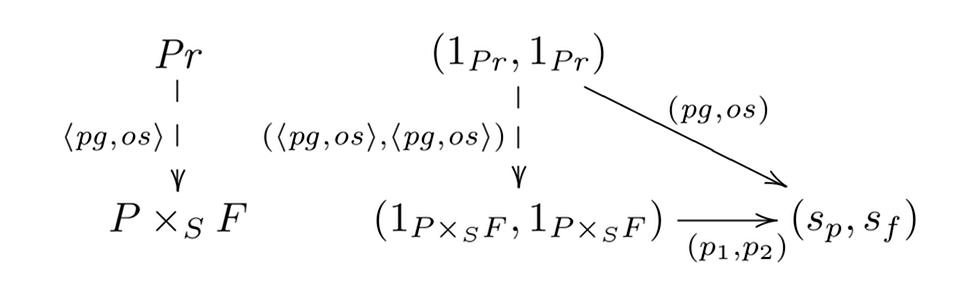

Supplement: Figure S16 — Diagonal-pullback adjoint for parent relation (counit). (TIF) [file pcbi.1002102.s016.tif]

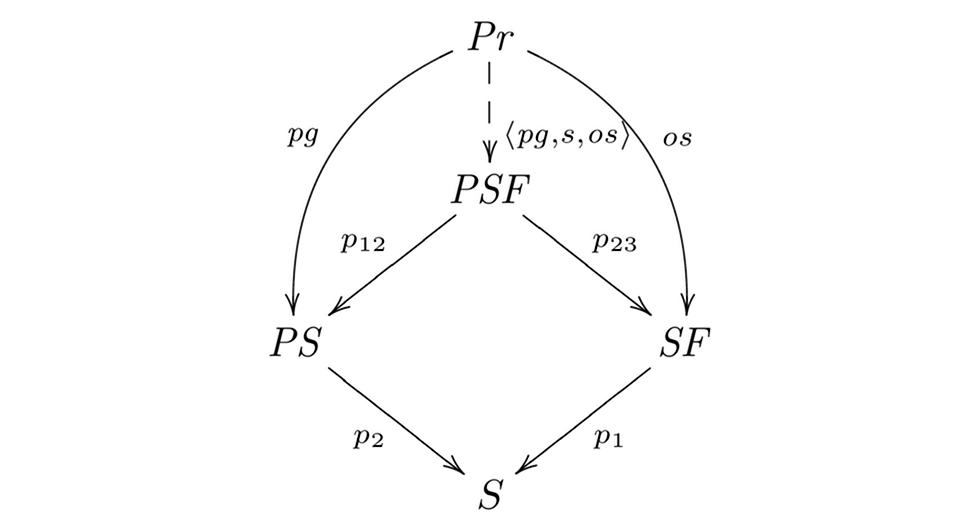

Supplement: Figure S17 — Diagonal-pullback adjoint for extended parent relation. (TIF) [file pcbi.1002102.s017.tif]

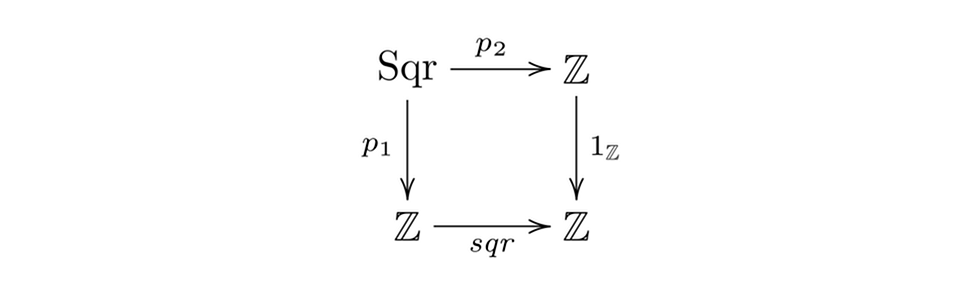

Supplement: Figure S18 — Pullback for square relation. (TIF) [file pcbi.1002102.s018.tif]

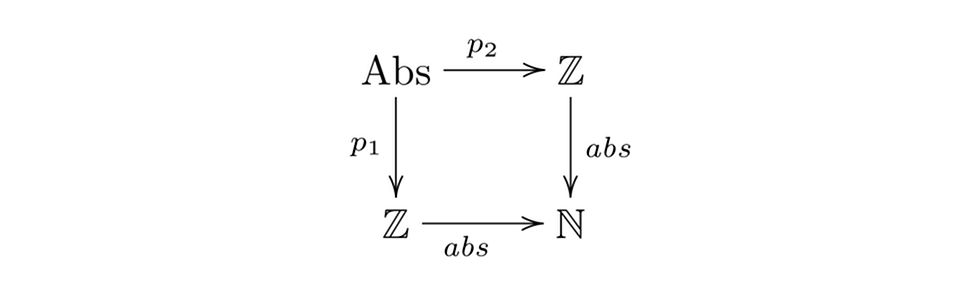

Supplement: Figure S19 — Pullback for absolute relation. (TIF) [file pcbi.1002102.s019.tif]

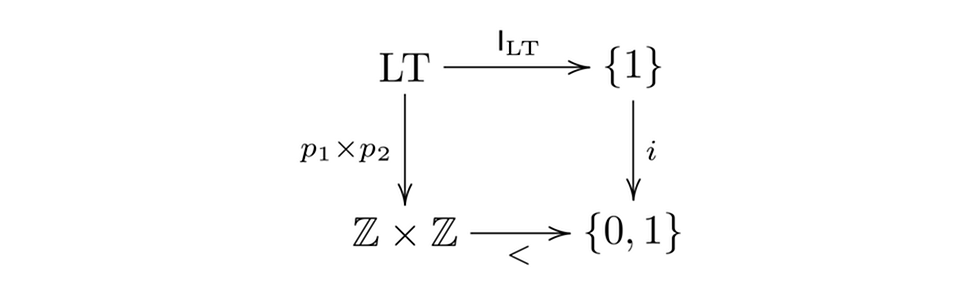

Supplement: Figure S20 — Pullback for less-than relation. (TIF) [file pcbi.1002102.s020.tif]

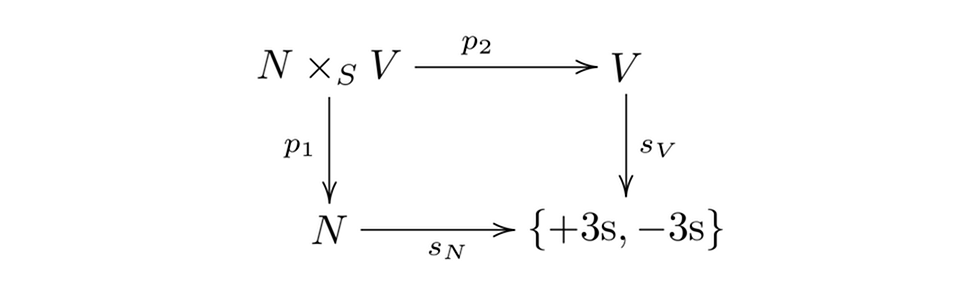

Supplement: Figure S21 — Pullback for subject-verb agreement. (TIF) [file pcbi.1002102.s021.tif]

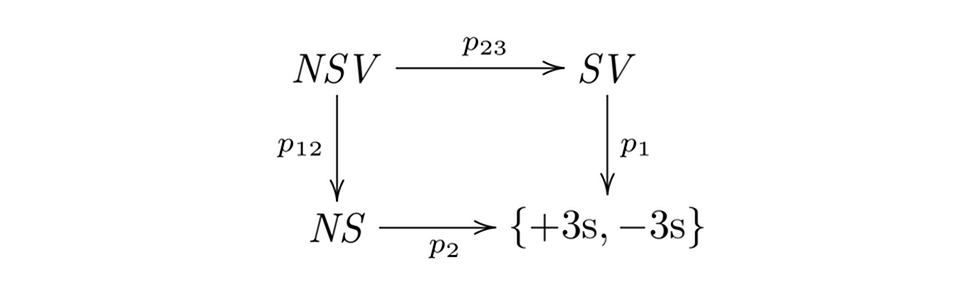

Supplement: Figure S22 — Pullback for extended subject-verb agreement. (TIF) [file pcbi.1002102.s022.tif]

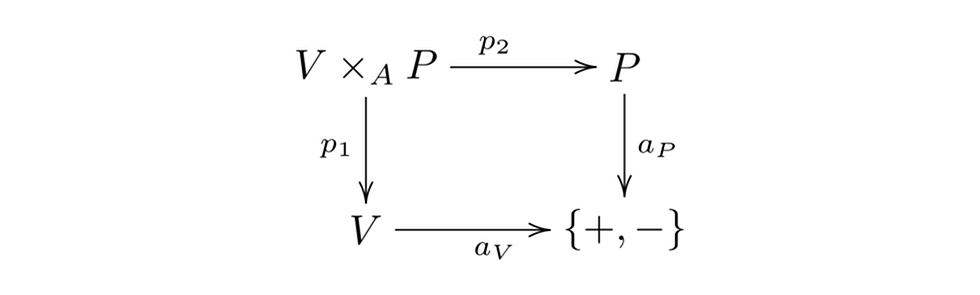

Supplement: Figure S23 — Pullback for prepositions. (TIF) [file pcbi.1002102.s023.tif]
