# Supplementary material for: Categorial Compositionality II: Universal Constructions and a General Theory of (Quasi-)Systematicity in Human Cognition
Source: PLoS Comput Biol. 2011 Aug 4;7(8):e1002102. doi: 10.1371/journal.pcbi.1002102 (PMC3154512; doi:10.1371/journal.pcbi.1002102)
Supplement: Text S1 — Definitions of category theory notations used in the main text. (PDF) [file pcbi.1002102.s024.pdf]

Text S1

Notation

Symbol	Referent
$\varnothing$	empty set
$\in$	is a member of
$\subseteq$	is a subset of
$\cong$	is isomorphic to
$\ncong$	is not isomorphic to
$\dashv$	is left adjoint to
$\mapsto$	maps to
$\rightarrow$	arrow, morphism, map
$\dashrightarrow$	uniquely existing arrow
$\dot{\rightarrow}$	natural transformation
$A, B, C, \ldots$	objects
$f, g, h, \ldots$	morphisms
$F, G, H, \ldots$	functors
$\eta, \epsilon, \ldots$	natural transformations
$\mathbf{C}, \mathbf{D}, \ldots$	categories
$\lvert\mathbf{C}\rvert$	the class of objects in category $\mathbf{C}$
$f : A \rightarrow B$	morphism f from domain object A to codomain object B
$F : \mathbf{C} \rightarrow \mathbf{D}$	functor F from domain category $\mathbf{C}$ to codomain category $\mathbf{D}$
$\eta : F \dot{\rightarrow} G$	natural transformation η from domain functor F to codomain functor G
**Set**	category with sets for objects and functions for morphisms
$\mathbf{0}, \mathbf{1}, \mathbf{2}$	category with zero, one, two objects and no non-identity morphisms
$(T \downarrow S)$	comma category from functors T and S
$\mathbf{D}^{\mathbf{C}}$, Funct$(\mathbf{C}, \mathbf{D})$	category of functors from $\mathbf{C}$ to $\mathbf{D}$ and natural transformations
**J**	shape category

$\mathbf{0}$,	empty shape category
$\mathbf{1}$, $\cdot$	(one-object discrete) singleton shape category
$\mathbf{2}$, $\cdot\quad\cdot$	(two-object discrete) pair shape category
$\downdownarrows$, $\cdot\rightrightarrows\cdot$	parallel shape category
$\divideontimes$, $\cdot\longrightarrow\cdot\longleftarrow\cdot$	sink shape category
$\leftrightarrow$, $\cdot\longleftarrow\cdot\longrightarrow\cdot$	cosink shape category
$\mathbf{C}^{\mathbf{0}}$	category of empty shaped diagrams, $\mathbf{C}^{\mathbf{0}} \cong \mathbf{1}$
$\mathbf{C}^{\mathbf{2}}$	category of pair shaped diagrams, $\mathbf{C}^{\mathbf{2}} \cong \mathbf{C} \times \mathbf{C}$
$0, 1, \mathsf{0}$	initial, terminal, zero object
$*$	object in a one-object category, such as $\mathbf{1}$
$A \times B$	product of objects A and B
$A + B$	coproduct of objects A and B
$A \times_C B$	pullback (fiber product) of objects A and B constrained at C
$A +_C B$	pushout (fiber coproduct) of objects A and B constrained at C
(A, B)	pair of objects A and B
$f \circ g$	composition of morphism f with morphism g
$\langle f, g \rangle$	diverging pair of arrows, having a common domain
$[f, g]$	converging pair of arrows, having a common codomain
(f, g)	parallel pair of arrows, having distinct (co)domains
$f \times g$	product of arrows f and g
$\mathsf{0}_A : 0 \to A$	morphism from an initial object 0
$\mathsf{l}_A : A \to 1$	morphism to a terminal object 1
$1_A : A \to A$	identity morphism on object A
$1_{\mathbf{C}} : \mathbf{C} \to \mathbf{C}$	identity functor on category $\mathbf{C}$, $1_{\mathbf{C}} : X \mapsto X, f \mapsto f$
$1_F : F \dot{\to} F$	identity natural transformation on functor F, $1_{F_X} : F(X) \to F(X)$
$F_A : \mathbf{C} \to \mathbf{D}$	constant functor, $F_A : X \mapsto A, f \mapsto 1_A$, for all $X \in \lvert\mathbf{C}\rvert$
$D : \mathbf{J} \to \mathbf{C}$	diagram (functor) D of (from) shape $\mathbf{J}$ in (to) category $\mathbf{C}$

$D_A : \mathbf{J} \to \mathbf{C}$	constant diagram, $D_A : X \mapsto A, f \mapsto 1_A$, for all X, f in $\mathbf{J}$
$D_\varnothing : \mathbf{0} \to \mathbf{C}$	empty diagram
$\Delta_\mathbf{0} : \mathbf{C} \to \mathbf{C}^\mathbf{0}$	constant diagonal functor, $\Delta_\mathbf{0} : A \mapsto *, f \mapsto 1_*$, for all A, f in $\mathbf{C}$
$\Delta_\mathbf{2} : \mathbf{C} \to \mathbf{C}^\mathbf{2}$	pair diagonal functor, $\Delta_\mathbf{2} : A \mapsto (A, A), f \mapsto (f, f)$
$\Delta_{\downdownarrows} : \mathbf{C} \to \mathbf{C}^{\downdownarrows}$	parallel diagonal functor, $\Delta_{\downdownarrows} : A \mapsto (1_A, 1_A), f \mapsto (f, f)$
$\Delta_{\divideontimes} : \mathbf{C} \to \mathbf{C}^{\divideontimes}$	sink diagonal functor, $\Delta_{\divideontimes} : A \mapsto (1_A, 1_A), f \mapsto (f, f)$
$\Delta_{\leftrightarrow} : \mathbf{C} \to \mathbf{C}^{\leftrightarrow}$	cosink diagonal functor, $\Delta_{\leftrightarrow} : A \mapsto (1_A, 1_A), f \mapsto (f, f)$
$\Delta : \mathbf{C} \to \mathbf{C}^\mathbf{J}$	general diagonal functor, $\Delta : A \mapsto D_A, (f : X \to Y) \mapsto (\eta : D_A \dot{\to} D_B)$
V, W	vertex object V of a cone, vertex object W of a cocone
$\underleftarrow{L}$, $\underrightarrow{L}$	vertex $\underleftarrow{L}$ of a (limit/terminal) cone, vertex $\underrightarrow{L}$ of a (colimit/initial) cocone
(V, ϕ)	cone with vertex V and leg morphisms ϕ
(W, ψ)	cocone with vertex W and leg morphisms ψ
$\phi : D_V \dot{\to} D$	cone (natural transformation) ϕ with vertex V, $\phi_I : V \to D(I)$
$\psi : D \dot{\to} D_W$	cocone (natural transformation) ψ with vertex W, $\psi_I : D(I) \to W$
$\kappa : D_{\underleftarrow{L}} \dot{\to} D$	cone (natural transformation) κ with vertex $\underleftarrow{L}$, $\kappa_I : \underleftarrow{L} \to D(I)$
$\chi : D \dot{\to} D_{\underrightarrow{L}}$	cocone (natural transformation) χ with vertex $\underrightarrow{L}$, $\chi_I : D(I) \to \underrightarrow{L}$
$\underleftarrow{lim}_\mathbf{0} : D_\varnothing \dot{\to} D_\varnothing$	terminal limit (cone), $(1, 1_1)$
$\underrightarrow{lim}_\mathbf{0} : D_\varnothing \dot{\to} D_\varnothing$	initial limit (cocone), $(0, 0_1)$
$\underleftarrow{lim}_{\downdownarrows} : D_E \dot{\to} D$	equalizer limit (cone), (E, e)
$\underrightarrow{lim}_{\downdownarrows} : D \dot{\to} D_Q$	coequalizer limit (cocone), (Q, q)
$\underleftarrow{lim}_\mathbf{2} : D_{A \times B} \dot{\to} D$	product limit (cone), $(A \times B, p_i)$, for $i \in \{1, 2\}$
$\underrightarrow{lim}_\mathbf{2} : D \dot{\to} D_{A+B}$	coproduct limit (cocone), $(A + B, q_i)$, for $i \in \{1, 2\}$
$\underleftarrow{lim}_{\divideontimes} : D_{A \times_C B} \dot{\to} D$	pullback limit (cone), $(A \times_C B, p_i)$, for $i \in \{1, 2\}$
$\underrightarrow{lim}_{\leftrightarrow} : D \dot{\to} D_{A +_C B}$	pushout limit (cocone), $(A +_C B, q_i)$, for $i \in \{1, 2\}$
$\underleftarrow{lim} : D_{\underleftarrow{L}} \dot{\to} D$	general limit (cone), $\underleftarrow{lim}_X : \underleftarrow{L} \to D(X)$
$\underrightarrow{lim} : D \dot{\to} D_{\underrightarrow{L}}$	general colimit (cocone), $\underrightarrow{lim}_X : D(X) \to \underrightarrow{L}$
$\underleftarrow{Lim}_\mathbf{0} : \mathbf{C}^\mathbf{0} \to \mathbf{C}$	terminal (limit) functor, $\underleftarrow{Lim}_\mathbf{0} : * \mapsto 1, 1_* \mapsto 1_1$

$\varinjlim_{\mathbf{0}} : \mathbf{C}^{\mathbf{0}} \to \mathbf{C}$	initial (colimit) functor, $\varinjlim_{\mathbf{0}} : * \mapsto 0, 1_* \mapsto 1_0$
$\varprojlim_{\downdownarrows} : \mathbf{C}^{\downdownarrows} \to \mathbf{C}$	equalizer (limit) functor, $\varprojlim_{\downdownarrows} : (f, g) \mapsto (E, e), (h, f \circ h) \mapsto h$
$\varinjlim_{\downdownarrows} : \mathbf{C}^{\downdownarrows} \to \mathbf{C}$	coequalizer (colimit) functor, $\varinjlim_{\downdownarrows} : (f, g) \mapsto (Q, q), (h \circ f, f) \mapsto h$
$\varprojlim_{\mathbf{2}}, \Pi : \mathbf{C}^{\mathbf{2}} \to \mathbf{C}$	product (limit) functor, $\Pi : (A, B) \mapsto A \times B, (f, g) \mapsto f \times g$
$\varinjlim_{\mathbf{2}}, \amalg : \mathbf{C}^{\mathbf{2}} \to \mathbf{C}$	coproduct (colimit) functor, $\amalg : (A, B) \mapsto A + B, (f, g) \mapsto f + g$
$\varprojlim_{\divideontimes}, \Pi_C : \mathbf{C}^{\divideontimes} \to \mathbf{C}$	pullback (limit) functor, $\Pi_C : (A, B) \mapsto A \times_C B, (f, g) \mapsto f \times g$
$\varinjlim_{\leftrightarrow}, \amalg_C : \mathbf{C}^{\leftrightarrow} \to \mathbf{C}$	pushout (colimit) functor, $\amalg_C : (A, B) \mapsto A +_C B, (f, g) \mapsto f + g$
$\varprojlim : \mathbf{C}^{\mathbf{J}} \to \mathbf{C}$	general limit functor, $\varprojlim : D \mapsto \underleftarrow{L}, (\eta : D \to D') \mapsto (\underleftarrow{L} \to \underleftarrow{L}')$
$\varinjlim : \mathbf{C}^{\mathbf{J}} \to \mathbf{C}$	general colimit functor, $\varinjlim : D \mapsto \underrightarrow{L}, (\eta : D \dot{\to} D') \mapsto (\underrightarrow{L} \to \underrightarrow{L}')$
