# Supplementary material for: Categorial Compositionality II: Universal Constructions and a General Theory of (Quasi-)Systematicity in Human Cognition
Source: PLoS Comput Biol. 2011 Aug 4;7(8):e1002102. doi: 10.1371/journal.pcbi.1002102 (PMC3154512; doi:10.1371/journal.pcbi.1002102)
Supplement: Text S2 — Further explanation of the category theory concepts used to explain systematicity, and how these concepts are related to each other, their duals, more general forms, and to the unifying concept of comma category. (PDF) [file pcbi.1002102.s025.pdf]

Text S2

Comma categories are intimately linked to all the category theory concepts we employ in our explanation of systematicity. Hence, they provide a formal unifying framework from which we derive the other concepts. In particular, the section "Four specific limits from comma categories" instantiates the limit process for the four limits in similar ways. This material draws on established category theory results found in many introductions to the field. The intention, here, is to make our approach to the systematicity problem more accessible to the reader unfamiliar with category theory. Hence, our style is expository, and diagrams are augmented with details normally left implicit in category theory texts. Though the content is not new, as far as we know, these concepts and their inter-relationships have not been brought together in this way before. A summary of the major category theory concepts and their relationships is provided in Figure 1 and 2 at the end of this text.

Preliminary examples: Functor, natural transformation

Functors and natural transforamtions are components of most of the concepts derived from comma categories. Some examples are provided to aid understanding of these and subsequence concepts.

Functor

A simple example constructs multiplication from addition via exponentiation: $e^{x+y} = e^x \times e^y$. A *monoid* is a one-object category; the object labeled $*$ if no specific label is needed. Then $(\mathbb{R}, +, 0)$ and $(\mathbb{R}, \times, 1)$ are monoids, where each $x \in \mathbb{R}$ (the set of real numbers) is associated with a morphism $x : * \to *$, with 0 and 1 as the identities and addition $(+)$ and multiplication $(\times)$ as compositions (respectively). $F : (\mathbb{R}, +, 0) \to (\mathbb{R}, \times, 1), * \mapsto *; x \mapsto e^x$ is a functor, where base e is a positive real number, i.e., $F(x + y) = F(x) \times F(y)$. The mapping $G : (\mathbb{R}, +, 0) \to (\mathbb{R}, +, 0), x \mapsto e^x$ is not a functor, since $e^{x+y} \neq e^x + e^y$, for all $x, y \in \mathbb{R}$.

Natural transformation

The following example of a natural transformation involves modular arithmetic. Consider the category **Clock{24}** having a single object (a 24-hour clock), C24 $= \{0, 1, \ldots, 23\}$, and 24 morphisms, $a^{24}_i : h \mapsto (h + i) \bmod 24$, $i = 0 \ldots 23$, for adjusting time by i hour increments, where a^{24}_0 is the identity,

and $a_h^{24} \circ a_g^{24} = a_{(h+g) \bmod 24}^{24}$. Extend this idea to produce the category $\mathbf{Clock\{3,4,12,24\}}$, with a 3-hour (C3), a 4-hour (C4), a 12-hour (C12), and a 24-hour (C24) clock (object), *endomorphisms* $a_i^H : h \mapsto (h+i) \bmod H$, for $H \in \{3,4,12,24\}$ and $i \in \{0,1,\ldots,H\}$, and any further morphisms required by the axioms. An endomorphism is a morphism whose domain is the same as its codomain. Functor $F_3 : \mathbf{Clock\{24\}} \rightarrow \mathbf{Clock\{3,4,12,24\}}, \mathrm{C24} \mapsto \mathrm{C3}; a_i^{24} \mapsto a_{i \bmod 3}^3$ constructs a 3-hour clock from a 24-hour clock. It is routine to show that F_3 is a functor, likewise F_4, F_{12}, and F_{24}, which are defined similarly. F_{24} is injective, sending C24 and its associated morphisms in $\mathbf{Clock\{24\}}$ to themselves in $\mathbf{Clock\{3,4,12,24\}}$. Natural transformation $\alpha : F_{24} \dot{\rightarrow} F_{12}$, where $\alpha_{\mathrm{C24}} : h \mapsto h \bmod 12$, views a 24-hour clock as a 12-hour clock in the obvious way via associated commuting squares (i.e., $\alpha_{\mathrm{C24}} \circ a_i^{24} = a_{i \bmod 12}^{12} \circ \alpha_{\mathrm{C24}}$, for all $i \in \{0,\ldots,23\}$, which is also routine to show, noting that $F_{12}(a_i^{24}) = a_{i \bmod 12}^{12}$). In contrast, there is no map $\beta : F_4 \rightarrow F_3$ that is a natural transformation: essentially, the clocks become desynchronized by a one-cycle adjustment (i.e., $F_4(a_4^4) = a_0^4 : h \mapsto h$, but $F_3(a_4^3) = a_1^3 : h \mapsto h+1$).

Comma category

A comma category is constructed from two functors with the same codomain. Specifically, suppose functors $T : \mathbf{A} \rightarrow \mathbf{C}$ and $S : \mathbf{B} \rightarrow \mathbf{C}$, indicated in the following diagram:

$$\mathbf{A} \xrightarrow{T} \mathbf{C} \xleftarrow{S} \mathbf{B} \tag{1}$$

The *comma category* $(T \downarrow S)$ formed from these two functors consists of:

- *objects* that are triples (A, B, f), where A is an object in $\mathbf{A}$, B is an object in $\mathbf{B}$, and $f : T(A) \rightarrow S(B)$ is a morphism in $\mathbf{C}$; and
- *morphisms* that are pairs $(g, h) : (A_1, B_1, f_1) \rightarrow (A_2, B_2, f_2)$, where $g : A_1 \rightarrow A_2$ and $h : B_1 \rightarrow B_2$ are morphisms in $\mathbf{A}$ and $\mathbf{B}$, respectively, such that the following diagram commutes:

$$\begin{array}{ccc} T(A_1) & \xrightarrow{f_1} & S(B_1) \\ {\scriptstyle T(g)}\downarrow & & \downarrow{\scriptstyle S(h)} \\ T(A_2) & \xrightarrow[f_2]{} & S(B_2) \end{array} \tag{2}$$

The identity morphism on (A, B, f) is $(1_A, 1_B)$, and the composition of morphisms $(g', h') \circ (g, h)$ is $(g' \circ g, h' \circ h)$ whenever the latter expression is defined.

The constructive relationship between functors T and S and the category $(T \downarrow S)$ is illustrated in the following diagram:

$$
\begin{array}{ccccc}
\mathbf{A} \xrightarrow{T} \mathbf{C} & & \mathbf{C} \xleftarrow{S} \mathbf{B} & & (T \downarrow S) \\
\\
A_1 & T(A_1) \xrightarrow{f_1} S(B_1) & & B_1 & (A_1, B_1, f_1) \\
\downarrow g & T(g) \downarrow \qquad\qquad \downarrow S(h) & & h \downarrow & (g,h) \downarrow \\
A_2 & T(A_2) \xrightarrow[f_2]{} S(B_2) & & B_2 & (A_2, B_2, f_2)
\end{array}
\tag{3}
$$

where the far right column indicates the constructed comma category, and its objects and morphisms. A natural transformation relates to a special case of a comma category where the two functors have the same domain (i.e., $\mathbf{A} = \mathbf{B}$ and $T, S : \mathbf{A} \rightarrow \mathbf{C}$ in the above example). The difference is that the natural transformation identifies just *one* particular collection of morphisms (i.e., one horizontal arrow for each object) making the square commute, whereas the comma category is a construction containing *all* pairs of morphisms (i.e., vertical arrows) making the squares commute.

Universality

Comma categories are also used to provide a formal basis for universality, in the form of universal constructions. A universal construction relates to an object or morphism that has some property that is shared by all objects or morphisms in its category. For example, in a category $\mathbf{C}$ whose objects are members of a finite set of numbers and morphisms are less-than-or-equal relationships (i.e., $A \leq B$, so in such a category there is either one or zero morphisms between any pair of distinct objects), the minimum number has the universal property of being less than or equal to every number in the category. Conversely, the maximum number has the universal property that every number in the category is less than or equal to it. The minimum and maximum numbers in this category are examples of *initial* and *terminal* objects (respectively), which we will define shortly. From a comma category perspective, the concept of a universal construction is generalized in terms of a particular kind of comma category whose objects are particular kinds of morphisms, where a universal construction is a (co)universal morphism, i.e., (initial) terminal object in that comma category. (The prefix “co” is often used when naming a

dual concept, e.g., *coproduct* as the dual of *product*.) So, the definitions of universal constructions and particular cases, adjunctions and limits, depend on the definitions of (co)universal morphism and (initial) terminal object, which we provide first.

Initial and terminal objects

Initial and terminal objects (if they exist) have a property shared by all objects in the category.

An *initial object* in a category $\mathbf{C}$ is an object, denoted 0, such that for every object $A \in |\mathbf{C}|$ there exists a unique morphism $u : 0 \to A$ in $\mathbf{C}$.

A *terminal object* in a category $\mathbf{C}$ is an object, denoted 1, such that for every object $A \in |\mathbf{C}|$ there exists a unique morphism $u : A \to 1$ in $\mathbf{C}$.

(A *zero object* in $\mathbf{C}$, denoted $\mathsf{0}$, is an object that is both an initial and terminal object in $\mathbf{C}$.)

Not all categories have initial, terminal, or zero objects. In each case, when more than one such object exists, they are isomorphic.

Universal morphism

Given a functor $F : \mathbf{A} \to \mathbf{C}$ and an object $Y \in |\mathbf{C}|$, a *universal morphism* from F to Y is a pair (A, ϕ) where A is an object of $\mathbf{A}$, and ϕ is a morphism in $\mathbf{C}$, such that for every object $X \in |\mathbf{A}|$ and every morphism $f : F(X) \to Y$, there exists a unique morphism $h : X \to A$, such that $\phi \circ F(h) = f$, as indicated by commutative diagram

$$\begin{array}{ccccc} X & \quad & F(X) & & \\ \Big\downarrow h & & F(h)\Big\downarrow & \searrow f & \\ A & & F(A) & \xrightarrow[\phi]{} & Y \end{array} \tag{4}$$

From a comma category perspective, a universal morphism is a terminal object in the comma category $(F \downarrow S_Y)$ of morphisms from functor F to object Y, also denoted $(F \downarrow Y)$, indicated by diagram

$$\mathbf{A} \xrightarrow{F} \mathbf{C} \xleftarrow{S_Y} \mathbf{B} \tag{5}$$

where S_Y is a constant functor selecting Y and 1_Y in $\mathbf{C}$. The corresponding objects and morphisms of

this category are indicated in the following diagram:

$$\mathbf{A} \xrightarrow{F} \mathbf{C} \qquad \mathbf{C} \xleftarrow{S_Y} \mathbf{B} \qquad (F \downarrow Y) \tag{6}$$

$$\begin{array}{ccccccc}
A_1 & & F(A_1) & \xrightarrow{f_1} & Y & B_1 & (A_1, B_1, f_1) \\
\downarrow{\scriptstyle h} & & {\scriptstyle F(h)}\downarrow & & \downarrow{\scriptstyle 1_Y} & {\scriptstyle k}\downarrow & {\scriptstyle (h,k)}\downarrow \\
A_2 & & F(A_2) & \xrightarrow[f_2]{} & Y & B_2 & (A_2, B_2, f_2)
\end{array}$$

Diagram 6 can be simplified by noting that the objects and morphisms of category **B** are ignored by functor S_Y, and the component arrow 1_Y is constant across all morphisms in this comma category. Replacing (A_2, f_2) with (A, ϕ), relabeling (A_1, f_1) as (X, f), and observing that h must be unique by the definition of universal morphism yields the following diagram:

$$\mathbf{A} \xrightarrow{F} \mathbf{C} \qquad \mathbf{C} \xleftarrow{S_Y} \mathbf{B} \qquad (F \downarrow Y) \tag{7}$$

$$\begin{array}{cccccc}
X & & F(X) & & & (X, f) \\
\vdots{\scriptstyle h} & & {\scriptstyle F(h)}\vdots & \searrow{\scriptstyle f} & & {\scriptstyle h}\vdots \\
A & & F(A) & \xrightarrow[\phi]{} & Y & (A, \phi)
\end{array}$$

which recovers Diagram 4, and universal morphism (A, ϕ) from comma category $(F \downarrow S_Y)$.

The reader may puzzle over the notion of a morphism from a functor F to an object X, since F and X are different kinds of constructs. A functor may be said to "live" in its codomain category. So, object A in universal morphism (A, ϕ) provides a reference to F, as the object $F(A)$, which lives in the same category as X. The same situation also applies to couniversal morphism, presented next.

The concept of a universal morphism is analogous to the concept of a universal (terminal) object. A terminal object is connected to every object in its category. A universal morphism is a common factor of every morphism in its category. That is, every morphism is composed from a universal morphism, if one exists. If we interpret morphisms as processes, then a universal morphism is a (sub)process common to all processes. For example, the process of recognizing an arbitrary character may be composed of a normalization step (e.g., centering the character) followed by a template matching step, which is a universal morphism.

Couniversal morphism

Given an object $X \in |\mathbf{C}|$ and a functor $F : \mathbf{B} \to \mathbf{C}$, a *couniversal morphism* from X to F is a pair (B, ψ) where B is an object of $\mathbf{B}$, and ψ is a morphism in $\mathbf{C}$, such that for every object $Y \in |\mathbf{B}|$ and every morphism $f : X \to F(Y)$, there exists a unique morphism $k : B \to Y$, such that $F(k) \circ \psi = f$, as indicated by commutative diagram

$$\begin{array}{ccccc} X & \xrightarrow{\psi} & F(B) & \qquad & B \\ & \searrow^{f} & \Big\downarrow{\scriptstyle F(k)} & & {\scriptstyle k}\Big\downarrow \\ & & F(Y) & & Y \end{array} \tag{8}$$

From a comma category perspective, a couniversal morphism is an initial object in the comma category $(T_X \downarrow F)$ of morphisms from object X to functor F, also denoted $(X \downarrow F)$, indicated by diagram

$$\mathbf{A} \xrightarrow{T_X} \mathbf{C} \xleftarrow{F} \mathbf{B} \tag{9}$$

where T_X is a constant functor selecting X and 1_X in $\mathbf{C}$. The corresponding objects and morphisms of this category are indicated in the following diagram:

$$\begin{array}{ccccc} \mathbf{A} \xrightarrow{T_X} \mathbf{C} & \qquad & \mathbf{C} \xleftarrow{\quad F \quad} \mathbf{B} & \qquad & (X \downarrow F) \\ & & & & \\ \begin{array}{c} A_1 \\ \Big\downarrow{\scriptstyle h} \\ A_2 \end{array} & & \begin{array}{ccccc} X & \xrightarrow{f_1} & F(B_1) & & B_1 \\ {\scriptstyle 1_X}\Big\downarrow & & \Big\downarrow{\scriptstyle F(k)} & & {\scriptstyle k}\Big\downarrow \\ X & \xrightarrow[f_2]{} & F(B_2) & & B_2 \end{array} & & \begin{array}{c} (A_1, B_1, f_1) \\ {\scriptstyle (h,k)}\Big\downarrow \\ (A_2, B_2, f_2) \end{array} \end{array} \tag{10}$$

Diagram 10 can be simplified by noting that the objects and morphisms of category $\mathbf{A}$ are ignored by functor T_X, and the component arrow 1_X is constant across all morphisms in this comma category. Replacing (B_1, f_1) with (B, ψ), relabeling (B_2, f_2) as (Y, f), and observing that k must be unique by the

definition of couniversal morphism yields the following diagram:

$$\mathbf{A} \xrightarrow{T_X} \mathbf{C} \qquad \mathbf{C} \xleftarrow{F} \mathbf{B} \qquad (X \downarrow F) \tag{11}$$

$$\begin{array}{ccccc} X & \xrightarrow{\psi} & F(B) & \quad B & \quad (B,\psi) \\ & \searrow^{f} & \downarrow F(k) & \quad k \downarrow & \quad k \downarrow \\ & & F(Y) & \quad Y & \quad (Y,f) \end{array}$$

which recovers Diagram 8, and couniversal morphism (B, ψ) from comma category $(T_X \downarrow F)$.

Universal construction

A *universal construction* is either a universal morphism, or (its dual) a couniversal morphism.

Adjunction

An adjoint situation between two categories is a special case of a universal construction in that every object in each category is part of a (co)universal morphism. As such, adjoints are also derived from special cases of comma categories.

An *adjunction* consists of a pair of functors $F : \mathbf{C} \to \mathbf{D}$, $G : \mathbf{D} \to \mathbf{C}$ and a natural transformation $\eta : 1_{\mathbf{C}} \dot{\to} (G \circ F)$, such that for every $\mathbf{C}$−object X, $\mathbf{D}$−object Y, and $\mathbf{C}$−map $f : X \to G(Y)$, there exists a unique $\mathbf{D}$−map $g : F(X) \to Y$, such that $G(g) \circ \eta_X = f$, as indicated by the following commutative diagram:

$$\begin{array}{cccc} X & \xrightarrow{\eta_X} & G \circ F(X) & \quad F(X) \\ & \searrow^{f} & \downarrow G(g) & \quad \downarrow g \\ & & G(Y) & \quad Y \end{array} \tag{12}$$

The two functors are called an *adjoint pair*, denoted (F, G), where F is the *left adjoint* of G (written, $F \dashv G$), and G is the *right adjoint* of F. Equivalently, given functors F and G above, an adjunction also consists of a natural transformation $\epsilon : (F \circ G) \dot{\to} 1_{\mathbf{D}}$, such that for every $\mathbf{D}$−object Y, $\mathbf{C}$−object X, and $\mathbf{D}$−map $g : F(X) \to Y$, there exists a unique $\mathbf{C}$−map $f : X \to G(Y)$, such that $\epsilon_Y \circ F(f) = g$, as

indicated by the following commutative diagram:

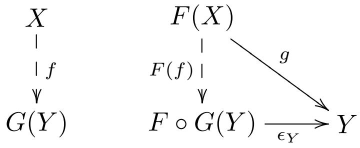

(13)

Proof that this definition of adjunction is equivalent to the one given above can be found in [1], p.83.

Under certain conditions (made explicit below), an adjunction expresses a relationship between two comma categories. We show this relationship using an equivalent definition of an adjunction that uses the definition of (co)free object. Given a functor $G : \mathbf{D} \rightarrow \mathbf{C}$ and an object $X \in |\mathbf{C}|$, $F(X) \in |\mathbf{D}|$ is the *free object* on X if there is a morphism $\eta_X : X \rightarrow G \circ F(X)$ in $\mathbf{C}$, such that for every object $Y \in |\mathbf{D}|$ and morphism $f : X \rightarrow G(Y)$ in $\mathbf{C}$, there exists a unique morphism $g : F(X) \rightarrow Y$ in $\mathbf{D}$, such that $G(g) \circ \eta_X = f$. (An object is called free in the context of G being an *underlying* or *forgetful* functor, i.e., a functor that maps objects to their underlying structure, such as vector spaces to their underlying sets. Though not all adjoint situations involve underlying functors in any obvious sense, we maintain this label for lack of a suitable alternative.)

An example of a free object follows (adapted from [1], p.56): Let **Vect** denote the category of all vector spaces over the real numbers, with arrows linear transformations, while $U : \mathbf{Vect} \rightarrow \mathbf{Set}$ is the underlying functor. For any set X there is a familiar vector space V_X with X as a set of basis vectors; it consists of all formal linear combinations of the elements of X. The function which sends each $x \in X$ into the same x regarded as a vector of V_X is a morphism $j : X \rightarrow U(V_X)$. For any other vector space W, it is a fact that each function $f : X \rightarrow U(W)$ can be extended to a unique linear transformation $f' : V_X \rightarrow W$ with $U(f') \circ j = f$. This fact, well-known to mathematicians, states exactly that j is a couniversal morphism from X to U, and V_X is a free object on X.

An adjoint situation arises from a pair of functors $F : \mathbf{C} \rightarrow \mathbf{D}$ and $G : \mathbf{D} \rightarrow \mathbf{C}$, and a natural transformation $\eta : 1_{\mathbf{C}} \dot{\rightarrow} G \circ F$, such that $F(X)$ is a free object on X for all $X \in |\mathbf{C}|$, where $F \dashv G$. This situation derives from Diagram 11 by replacing categories $\mathbf{A}$ and $\mathbf{B}$ with $\mathbf{C}$ and $\mathbf{D}$ (respectively), functors T_X and F with $1_{\mathbf{C}}$ and given functor G (respectively), and morphism ψ with η_X, such that for each $X \in |\mathbf{C}|$, $((F(X), \eta_X)$ is a couniversal morphism, i.e., an initial object in comma category $(1_{\mathbf{C}} \downarrow G)$,

where the natural transformation is $\eta : 1_{\mathbf{C}} \dot{\rightarrow} G \circ F$, as indicated in the following diagram:

$$
\begin{array}{ccc}
\mathbf{C} \xrightarrow{1_{\mathbf{C}}} \mathbf{C} & \mathbf{C} \underset{F}{\overset{G}{\rightleftarrows}} \mathbf{D} & (1_{\mathbf{C}} \downarrow G) \\
\begin{array}{ccc} X & \xrightarrow{\eta_X} & G \circ F(X) \\ & \searrow^{f} & \downarrow G(g) \\ & & G(Y) \end{array} & \begin{array}{c} F(X) \\ \downarrow g \\ Y \end{array} & \begin{array}{c} (F(X), \eta_X) \\ \downarrow g \\ (Y, f) \end{array}
\end{array}
\tag{14}
$$

(where in this diagram $F \dashv G$), which recovers Diagram 12. Equivalently, the definition of adjunction can also be built up from the dual concept of a *cofree* object. This adjoint situation derives from Diagram 7 by replacing category $\mathbf{A}$ with $\mathbf{C}$, categories $\mathbf{B}$ and $\mathbf{C}$ with $\mathbf{D}$, functor S_Y with $1_{\mathbf{D}}$, and morphism ϕ with ϵ_Y, such that for each $Y \in |\mathbf{D}|$, $((G(Y), \epsilon_Y)$ is a universal morphism, i.e., a terminal object in comma category $(F \downarrow 1_{\mathbf{D}})$, where the natural transformation is $\epsilon : F \circ G \dot{\rightarrow} 1_{\mathbf{D}}$, as indicated in the following diagram:

$$
\begin{array}{ccc}
\mathbf{C} \underset{G}{\overset{F}{\rightleftarrows}} \mathbf{D} & \mathbf{D} \xleftarrow{1_{\mathbf{D}}} \mathbf{D} & (F \downarrow 1_{\mathbf{D}}) \\
\begin{array}{c} X \\ \downarrow f \\ G(Y) \end{array} \quad \begin{array}{ccc} F(X) & & \\ F(f) \downarrow & \searrow^{g} & \\ F \circ G(Y) & \xrightarrow[\epsilon_Y]{} & Y \end{array} & & \begin{array}{c} (X, g) \\ \downarrow f \\ (G(Y), \epsilon_Y) \end{array}
\end{array}
\tag{15}
$$

Hence, the adjoint situation $F \dashv G$ expresses a relationship between the comma categories $(1_{\mathbf{C}} \downarrow G)$ and $(F \downarrow 1_{\mathbf{D}})$, such that the associated maps η and ϵ are natural transformations, and each η_X associated with $(1_{\mathbf{C}} \downarrow G)$ is a couniversal morphism from X to $G \circ F$ (respectively, each ϵ_Y associated with $(F \downarrow 1_{\mathbf{D}})$ is a universal morphism from $F \circ G$ to Y). Normally, η and ϵ are called the *unit* and *counit* of the adjunction, respectively, despite the fact that from the perspective of comma categories and universal constructions, η could be more consistently labeled as the counit, since it is associated with couniversal morphisms, and ϵ as the unit, since it is associated with universal morphisms.

A functor $F : \mathbf{C} \rightarrow \mathbf{D}$ may be the focus as either a left or a right adjoint. When the focus is as a right adjoint, $E \dashv F$, comma categories derive the corresponding adjunction from Diagram 14, or equivalently from Diagram 15, by setting the left and right adjoint functors to E and F (respectively), and corresponding categories and natural transformations appropriately. Hence, the adjoint situation $E \dashv F$

expresses a relationship between comma categories $(1_{\mathbf{D}} \downarrow F)$ and $(E \downarrow 1_{\mathbf{C}})$, such that the associated maps $\eta : 1_{\mathbf{D}} \dot{\rightarrow} F \circ E$ and $\epsilon : E \circ F \dot{\rightarrow} 1_{\mathbf{C}}$ are natural transformations, and each η_X associated with $(1_{\mathbf{D}} \downarrow F)$ is a couniversal morphism from X to $F \circ E$ (respectively, each ϵ_Y associated with $(E \downarrow 1_{\mathbf{C}})$ is a universal morphism from $E \circ F$ to Y).

The relationship between comma categories $(1_{\mathbf{C}} \downarrow G)$ and $(F \downarrow 1_{\mathbf{D}})$ is rendered more explicitly via another equivalent definition of adjunction in terms of hom-sets.

A *hom-set* $\hom_{\mathbf{C}}(A, B)$ is the set of morphisms in $\mathbf{C}$ having domain A and codomain B.

Equivalently, then, an *adjunction* consists of a pair of functors $F : \mathbf{C} \rightarrow \mathbf{D}$ and $G : \mathbf{D} \rightarrow \mathbf{C}$, and a family of bijections $\hom_{\mathbf{C}}(X, G(Y)) \cong \hom_{\mathbf{D}}(F(X), Y)$ that is *natural* in both X and Y. There is a one-to-one correspondence between the $\mathbf{C}-$arrows from X to $G(Y)$ and the $\mathbf{D}-$arrows from $F(X)$ to Y. The following diagram:

$$\begin{array}{ccc} X & \xrightarrow{F} & F(X) \\ \downarrow & & \downarrow \\ G(Y) & \xleftarrow[G]{} & Y \end{array} \tag{16}$$

informally highlights this correspondence.

From the hom-set definition of adjunction, we see that arrow $g : (F(X), \eta_X) \rightarrow (Y, f)$ in comma category $(1_{\mathbf{C}} \downarrow G)$, see Diagram 14, corresponds to arrow $f : (X, g) \rightarrow (G(Y), \epsilon_Y)$ in comma category $(F \downarrow 1_{\mathbf{D}})$, see Diagram 15. We denote this relationship by inserting "/" between the names of the related comma categories, thus: $(1_{\mathbf{C}} \downarrow G)/(F \downarrow 1_{\mathbf{D}})$, to reflect the sense that $(1_{\mathbf{C}} \downarrow G)$ and $(F \downarrow 1_{\mathbf{D}})$ are two sides of the same category theory construct.

Adjunction versus isomorphism

To contrast the concept of adjunction against isomorphism, we present a definition of isomorphic functor, state its relationship to adjoint functor, and provide an example that illustrates their difference

The composition of functors $F : \mathbf{C} \rightarrow \mathbf{D}$ and $G : \mathbf{D} \rightarrow \mathbf{E}$ is the functor $G \circ F : \mathbf{C} \rightarrow \mathbf{E}$, sending all objects A in $\mathbf{C}$ to objects $G \circ F(A)$ in $\mathbf{E}$; and morphisms $f : A \rightarrow B$ in $\mathbf{C}$ to morphisms $G \circ F(f) : G \circ F(A) \rightarrow G \circ F(B)$, such that identity and composition are respected. That is, $G \circ F(1_A) = 1_{G \circ F(A)}$; and $G \circ F(g \circ_{\mathbf{C}} f) = (G \circ F(g)) \circ_{\mathbf{E}} (G \circ F(f))$. A functor $F : \mathbf{C} \rightarrow \mathbf{D}$ is an *isomorphic functor*, if and only if there exists a functor $G : \mathbf{D} \rightarrow \mathbf{C}$ such that $G \circ F = 1_{\mathbf{C}}$ and $F \circ G = 1_{\mathbf{D}}$, where $1_{\mathbf{C}}$ and $1_{\mathbf{D}}$ are the identity functors sending objects and morphisms to themselves in the respective categories. In

this situation, category $\mathbf{C}$ is said to be *isomorphic* to category $\mathbf{D}$, written $\mathbf{C} \cong \mathbf{D}$, and functor G is the inverse of functor F, also written F^{-1}.

Every isomorphic functor $F : \mathbf{C} \to \mathbf{D}$ has a right and a left adjoint, which is its inverse $F^{-1} : \mathbf{D} \to \mathbf{C}$, i.e., $F \dashv F^{-1}$ and $F^{-1} \dashv F$. Conversely, an adjoint functor is not necessarily an isomorphic functor. Hence, two categories that are related by an adjoint situation are not necessarily isomorphic.

The fact that adjunctions are not necessarily isomorphisms is illustrated by a simple example. Suppose categories $\mathbf{C}$ and $\mathbf{D}$, and functors $F : \mathbf{C} \to \mathbf{D}$ and $G : \mathbf{D} \to \mathbf{C}$, such that $F \dashv G$, but $\mathbf{C} \ncong \mathbf{D}$, as indicated in the following diagram:

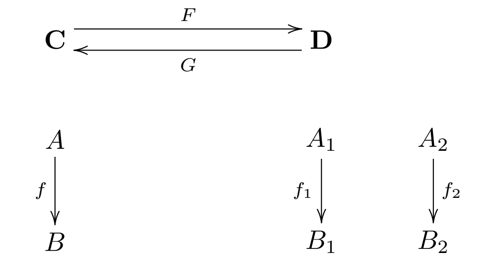

(17)

where $F : X \mapsto X_1, f \mapsto f_1$ and $G : X_i \mapsto X, f_i \mapsto f$, for $X \in \{A, B\}$ and $i \in \{1, 2\}$. Basically, F injects a copy of the objects and morphisms from $\mathbf{C}$ into $\mathbf{D}$, and G extracts those objects and morphisms. The adjoint situation is given in the following diagram:

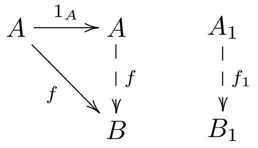

(18)

where 1_A is the unit of the adjunction. $\mathbf{D}$ is finite and contains more objects and morphisms than $\mathbf{C}$, so $\mathbf{C} \ncong \mathbf{D}$.

Limits

In category theory, the concept of a *limit* generalizes related concepts in other branches of mathematics (e.g., limit of a series) to involve entities other than just numbers. An informal example may help. A speed limit is the smallest value greater than or equal to all speeds at which one is legally permitted to drive on a given section of road. In a category of permitted speeds and less-than-or-equal-to morphisms (e.g., $90 \leq 100$), a speed limit is a terminal object (maximum permitted speed) in this category. (Not all categories have limits; not all roads have a limit on permitted speed.) Systematicity, though, may pertain

to domains whose objects also have (internal) structure. Binary relations, for example, are composed from a pair of constituent objects, where "pair" is the *shape* of the composition. A limit in this context is a composition of two objects together with morphisms for retrieving their constituents for each and every instance. This kind of limit, called a *product*, was employed in our explanation for the systematicity of binary relations [2]. Another limit, called a *pullback*, is used in our explanation of quasi-systematicity. These and other kinds of limits are constructed by a *general limit functor*, where the specific instances differ in the shape of the compositions. A general limit functor is right adjoint to the *general diagonal functor*, whose specific instances are likewise tied to shape. The various (diagonal, limit) functor pairs constitute adjoint situations, and hence further kinds of universal constructions that are derived from particular kinds of comma categories. (Dual constructs, called *colimits* and *colimit functors*, provide yet more kinds of universal constructions.) Before presenting (co)limits, we first need the concepts of a diagram and a (co)cone to a diagram.

Diagram

A *diagram* of *shape* $\mathbf{J}$ in category $\mathbf{C}$ is a functor $D : \mathbf{J} \to \mathbf{C}$.

The shape of a diagram is often a category with a small number of objects and morphisms. When the names of the objects in a shape category are not important, each such object is typically denoted in diagrams of shape categories by a single dot, $\cdot$. Several examples of shape categories are provides as they are used in the definitions of important examples of limits. They are:

empty The *empty shape* category, denoted $\mathbf{0}$, having no objects and morphisms, has an empty diagram

singleton The *singleton shape* category, denoted $\mathbf{1}$, has one object and no non-identity morphisms, as indicated by diagram

$$\cdot \tag{19}$$

pair The *pair shape* category, denoted $\mathbf{2}$, has two objects and no non-identity morphisms, as indicated by diagram

$$\cdot \qquad \cdot \tag{20}$$

parallel The *parallel shape* category, denoted $\downdownarrows$, has two objects and two non-identity morphisms sharing

the same domain and the same codomain, as indicated by diagram

$$\cdot \rightrightarrows \cdot \tag{21}$$

sink The *sink shape* category, denoted $\divideontimes$, has three objects and two non-identity morphisms sharing the same codomain, as indicated by diagram

$$\cdot \longrightarrow \cdot \longleftarrow \cdot \tag{22}$$

cosink The *cosink shape* category, denoted $\leftrightarrow$, has three objects and two non-identity morphisms sharing the same domain, as indicated by diagram

$$\cdot \longleftarrow \cdot \longrightarrow \cdot \tag{23}$$

The role of a diagram is analogous to the role of a function associated with an indexed set. An *indexed set* is a set S together with an *indexing function* $n : N \to S$ from *index* N. In our applications, N will be a subset of $\mathbb{N}$, the set of natural numbers. An index provides (ordered) reference points to the elements of S (e.g., {(0, red), (1, green), (2, blue)}), which are otherwise unordered and referenced only by themselves. Analogously, a diagram allows referencing a (part of a) category by its shape, and so $\mathbf{J}$ is analogous to an index.

(Co)Cone

A *cone* (V, ϕ) to a diagram $D : \mathbf{J} \to \mathbf{C}$ is a *vertex* object $V \in |\mathbf{C}|$ together with a family of morphisms $\phi = \{\phi_I | I \in \mathbf{J}\}$, containing one morphism $\phi_I : V \to D(I)$ in $\mathbf{C}$ for each object $I \in |\mathbf{J}|$, such that for every morphism $f : I \to J$ in $\mathbf{J}$ the following diagram commutes:

$$\begin{array}{ccc} & V & \\ {}^{\phi_I}\swarrow & & \searrow^{\phi_J} \\ D(I) & \xrightarrow[D(f)]{} & D(J) \end{array} \tag{24}$$

A cone (V, ϕ) to $D : \mathbf{J} \to \mathbf{C}$ is associated with the comma category $(D_V \downarrow D)$, where $D_V : \mathbf{J} \to \mathbf{C}$

is a constant diagram (functor), $D_V : I \mapsto V; f \mapsto 1_V$ for all I and f in $\mathbf{J}$, selecting vertex V and its identity morphism 1_V, and $D : \mathbf{J} \to \mathbf{C}$ is a diagram of shape $\mathbf{J}$ in $\mathbf{C}$. The following diagram shows the construction:

$$\begin{array}{ccccc} \mathbf{J} \xrightarrow{D_V} \mathbf{C} & & \mathbf{C} \xleftarrow{D} \mathbf{J} & & (D_V \downarrow D) \\ \\ \begin{array}{c} I \\ \downarrow f \\ J \end{array} & \begin{array}{ccc} V & \xrightarrow{\phi_I} & D(I) \\ {\scriptstyle 1_V}\downarrow & & \downarrow{\scriptstyle D(f)} \\ V & \xrightarrow[\phi_J]{} & D(J) \end{array} & \begin{array}{c} I \\ f\downarrow \\ J \end{array} & & \begin{array}{c} (I, \phi_I) \\ f\downarrow \\ (J, \phi_J) \end{array} \end{array} \tag{25}$$

The square in Diagram 25 simplifies to Diagram 24. Thus, a cone (V, ϕ) is the natural transformation $\phi : D_V \dot{\to} D$ associated with the comma category $(D_V \downarrow D)$, where the components of the natural transformation at V are ϕ_I and ϕ_J, as indicated in the following diagram:

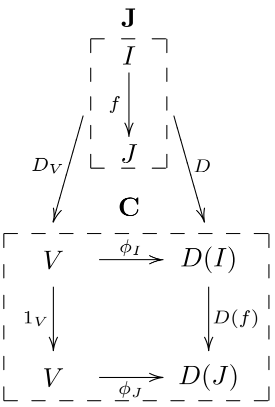

(26)

A *cocone* (W, ψ), the dual of a cone, from a diagram $D : \mathbf{J} \to \mathbf{C}$ is a vertex object $W \in |\mathbf{C}|$ together with a family of morphisms $\psi = \{\psi_I | I \in \mathbf{J}\}$, containing one morphism $\psi_I : D(I) \to W$ in $\mathbf{C}$ for each object $I \in |\mathbf{J}|$, such that for every morphism $f : I \to J$ in $\mathbf{J}$ the following diagram commutes:

$$\begin{array}{ccc} D(I) & \xrightarrow{D(f)} & D(J) \\ & {\scriptstyle \psi_I}\searrow \quad \swarrow{\scriptstyle \psi_J} & \\ & W & \end{array} \tag{27}$$

A cocone (W, ψ) from $D : \mathbf{J} \to \mathbf{C}$ is associated with the comma category $(D \downarrow D_W)$, where $D_W : \mathbf{J} \to \mathbf{C}$ is a constant diagram, selecting vertex W and its identity morphism 1_W. A cocone is derived by reversing the direction of the arrows of a cone. Thus, a cocone (W, ψ) is the natural transformation

$\psi : D \dot{\rightarrow} D_W$ associated with the comma category $(D \downarrow D_W)$, where the components of the natural transformation are $\psi_I : D(I) \rightarrow W$ and $\psi_J : D(J) \rightarrow W$

(Co)Limit

A *limit* of a diagram $D : \mathbf{J} \rightarrow \mathbf{C}$ is a cone $(\underleftarrow{L}, \kappa)$ to D such that for any cone (V, ϕ) to D there exists a unique morphism $u : V \rightarrow \underleftarrow{L}$ such that for all $I \in |\mathbf{J}|$ the following diagram commutes:

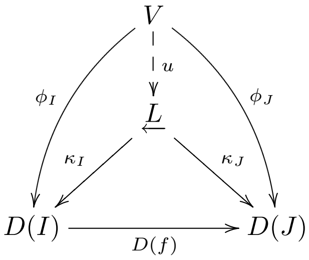

(28)

Since a limit is a cone, a limit $(\underleftarrow{L}, \kappa)$ is an object $\underleftarrow{L} \in \mathbf{C}$ and a natural transformation $\kappa : D_{\underleftarrow{L}} \dot{\rightarrow} D$ associated with the comma category $(D_{\underleftarrow{L}} \downarrow D)$, where the components of the natural transformation at $\underleftarrow{L}$ are $\kappa_I : \underleftarrow{L} \rightarrow D(I)$ and $\kappa_J : \underleftarrow{L} \rightarrow D(J)$. The limit natural transformation κ is also denoted $\varprojlim$.

A *colimit* of a diagram $D : \mathbf{J} \rightarrow \mathbf{C}$ is a cocone $(\underrightarrow{L}, \chi)$ from D such that for any cocone (W, ψ) from D there exists a unique morphism $u : \underrightarrow{L} \rightarrow W$ such that for all $I \in |\mathbf{J}|$ the following diagram commutes:

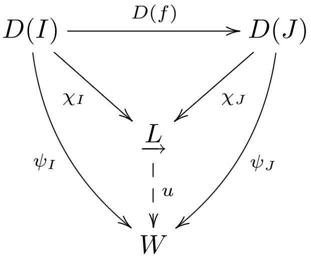

(29)

Since a colimit is a cocone, a colimit $(\underrightarrow{L}, \chi)$ is an object $\underrightarrow{L} \in \mathbf{C}$ and a natural transformation $\chi : D \dot{\rightarrow} D_{\underrightarrow{L}}$ associated with the comma category $(D \downarrow D_{\underrightarrow{L}})$, where the components of the natural transformation are $\chi_I : D(I) \rightarrow \underrightarrow{L}$ and $\chi_J : D(J) \rightarrow \underrightarrow{L}$. The colimit natural transformation χ is also denoted $\varinjlim$.

Limits as universal constructions from comma categories

A limit is a universal cone in a category of cones. As a universal construction, a limit is a universal morphism. Recall that a universal morphism is a terminal object in the comma category $(F \downarrow S_Y)$. Hence, a limit is a terminal object in a particular comma category. Likewise, a colimit is a couniversal cocone in a category of cocones, and an initial object in another particular comma category. To see the relationship between (co)limits and comma categories in terms of universal constructions, we need general functors for constructing diagrams and (co)cones, and for that we need the concept of a functor category.

Functor category

A *functor category* $(\mathbf{D}^{\mathbf{C}})$, or $\mathrm{Funct}(\mathbf{C}, \mathbf{D})$ has functors from category $\mathbf{C}$ to category $\mathbf{D}$ as objects and natural transformations between functors as morphisms. Diagrams are functors, and cones to diagrams are natural transformations. So, functor category $\mathbf{C}^{\mathbf{J}}$ has diagrams $D : \mathbf{J} \rightarrow \mathbf{C}$ of shape $\mathbf{J}$ in category $\mathbf{C}$ as objects and cones (V, ϕ) as special instances of morphisms $D_V \rightarrow D$, where D_V is a constant functor.

General diagonal functor

The general diagonal functor provides a means for constructing diagrams and (co)cones of arbitrary shape. First, we present the general case for cones, then their duals, cocones, before presenting specialized cases for (co)limits to diagrams of specific shapes.

The *general diagonal functor* is the functor $\Delta : \mathbf{C} \rightarrow \mathbf{C}^{\mathbf{J}}$ from category $\mathbf{C}$ to category of diagrams $\mathbf{C}^{\mathbf{J}}$. The object component of this functor is $\Delta : A \mapsto D_A$, where A is an object in $\mathbf{C}$ and $D_A : \mathbf{J} \rightarrow \mathbf{C}$ is a constant diagram in $\mathbf{C}^{\mathbf{J}}$, such that $D_A : I \mapsto A$ and $D_A : f \mapsto 1_A$, for all I and f in $\mathbf{C}$ (i.e., a constant functor that selects object A and its identity morphism 1_A). The morphism component of this functor is $\Delta : (g : A \rightarrow B) \mapsto (\eta : D_A \dot{\rightarrow} D_B)$, where $1_B \circ \eta = \eta \circ 1_A$ and η is set to g (which obviously makes the

square commute), as indicated in the following diagram:

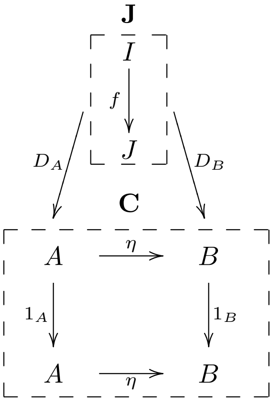

(30)

We saw that a limit $(\underleftarrow{L}, \kappa)$ is a universal morphism, i.e., the object (A, ϕ) in the comma category $(F \downarrow Y)$, given in Diagram 7. Since κ is a natural transformation between diagrams, the (co)domain objects of ϕ in Diagram 7 must be diagrams, and therefore category $\mathbf{C}$ in Diagram 7 must be the category of diagrams $\mathbf{C}^{\mathbf{J}}$ in the current context, and F is the general diagonal functor Δ. Hence, a limit $(\underleftarrow{L}, \kappa)$ is a terminal object in the comma category $(\Delta \downarrow Y)$, as indicated by the following diagram:

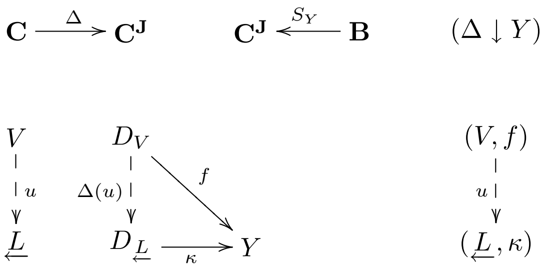

(31)

where S_Y is a constant functor selecting diagram Y.

We also saw that a colimit $(\underrightarrow{L}, \chi)$ is a couniversal morphism, i.e., the object (B, ψ) in the comma category $(X \downarrow F)$, given in Diagram 11. Hence, a colimit $(\underrightarrow{L}, \chi)$ is an initial object in the comma category

$(X \downarrow \Delta)$, as indicated by the following diagram:

$$\mathbf{A} \xrightarrow{T_X} \mathbf{C}^{\mathbf{J}} \qquad \mathbf{C}^{\mathbf{J}} \xleftarrow{\Delta} \mathbf{C} \qquad (X \downarrow \Delta) \tag{32}$$

$$\begin{array}{ccccc} X & \xrightarrow{\chi} & D_{\underrightarrow{L}} & \underrightarrow{L} & (\underrightarrow{L}, \chi) \\ & \searrow^{f} & \downarrow \Delta(u) & u \downarrow & u \downarrow \\ & & D_W & W & (W, f) \end{array}$$

where T_X is a constant functor selecting diagram X.

The general diagonal functor also has left (right) adjoints when colimits (limits) exist for a particular category $\mathbf{C}$ and shape $\mathbf{J}$, and so pertains to two kinds of adjunctions. Recall that an adjunction between two categories is a special case of a universal construction where every object in each category is part of (co)universal morphism. Hence, the general diagonal functor together with its left (right) adjoint form another special case of a universal construction. The right adjoint to the general diagonal functor is the general limit functor, and the left adjoint is the general colimit functor. We provide their definitions and then show how together they form two more kinds of universal constructions from comma categories.

General (co)limit functor

Provided that $\mathbf{C}$ has limits for $\mathbf{J}$−diagrams, there is a *general limit functor* $\underleftarrow{Lim} : \mathbf{C}^{\mathbf{J}} \to \mathbf{C}$ from category of diagrams $\mathbf{C}^{\mathbf{J}}$ to category $\mathbf{C}$. The object component of this functor $\underleftarrow{Lim} : D \mapsto \underleftarrow{L}$ maps each diagram $D : \mathbf{J} \to \mathbf{C}$ to its limit $\underleftarrow{L}$. The morphism component of this functor $\underleftarrow{Lim} : (\eta : D \dot{\to} D') \to (u : \underleftarrow{L} \to \underleftarrow{L}')$ maps each natural transformation η between diagrams to the unique morphism u that commutes with the corresponding universal cones.

The adjoint situation $\Delta \dashv \underleftarrow{Lim}$ arises from the comma category correspondence $(1_{\mathbf{C}} \downarrow \underleftarrow{Lim})/(\Delta \downarrow 1_{\mathbf{C}^{\mathbf{J}}})$

by substituting $\mathbf{C^J}$ for $\mathbf{D}$, Δ for F and $\underleftarrow{Lim}$ for G in Diagram 14, as indicated by the following diagram:

$$\mathbf{C} \xrightarrow{1_{\mathbf{C}}} \mathbf{C} \qquad \mathbf{C} \underset{\Delta}{\overset{\underleftarrow{Lim}}{\leftrightarrows}} \mathbf{C^J} \qquad (1_{\mathbf{C}} \downarrow \underleftarrow{Lim}) \tag{33}$$

$$\begin{array}{ccc} X \xrightarrow{\eta_X} \underleftarrow{Lim} \circ D_X & D_X & (D_X, \eta_X) \\ {\scriptstyle f} \searrow \quad \downarrow {\scriptstyle \underleftarrow{Lim}(g)} & {\scriptstyle g} \downarrow & {\scriptstyle g} \downarrow \\ D_Y & Y & (Y, f) \end{array}$$

where $\Delta(X) = D_X$, and in Diagram 15, as indicated by

$$\mathbf{C} \underset{\underleftarrow{Lim}}{\overset{\Delta}{\rightleftarrows}} \mathbf{C^J} \qquad \mathbf{C^J} \xleftarrow{1_{\mathbf{C^J}}} \mathbf{C^J} \qquad (\Delta \downarrow 1_{\mathbf{C^J}}) \tag{34}$$

$$\begin{array}{ccc} X & D_X & (X, g) \\ \downarrow {\scriptstyle f} & {\scriptstyle \Delta(f)} \downarrow \quad \searrow {\scriptstyle g} & {\scriptstyle f} \downarrow \\ \underleftarrow{Lim}(Y) & D_{\underleftarrow{Lim}(Y)} \xrightarrow[\epsilon_Y]{} Y & (\underleftarrow{Lim}(Y), \epsilon_Y) \end{array}$$

Provided that $\mathbf{C}$ has colimits for $\mathbf{J}$−diagrams, there is a *general colimit functor* $\underrightarrow{Lim} : \mathbf{C^J} \to \mathbf{C}$ from category of diagrams $\mathbf{C^J}$ to category $\mathbf{C}$. The object component of this functor $\underrightarrow{Lim} : D \mapsto \underrightarrow{L}$ maps each diagram $D : \mathbf{J} \to \mathbf{C}$ to colimit $\underrightarrow{L}$. The morphism component of this functor $\underrightarrow{Lim} : (\eta : D \dot{\to} D') \mapsto (u : \underrightarrow{L} \to \underrightarrow{L'})$ maps each natural transformation between diagrams η to the unique morphism u, which commutes with the corresponding couniversal cocones.

Similarly, the adjoint situation $\Delta \dashv \underrightarrow{Lim}$ arises from the comma category correspondence $(1_{\mathbf{C^J}} \downarrow \Delta)/(\underrightarrow{Lim} \downarrow 1_{\mathbf{C}})$ by substituting $\mathbf{C^J}$ for $\mathbf{D}$, $\underrightarrow{Lim}$ for F and Δ for G in Diagram 14 and Diagram 15.

Four specific limits from comma categories

From the general diagonal and limit functors, we are going to realize four specific (co)limits from comma categories by specifying diagrams of particular shapes. Five example shape categories mentioned earlier: empty ($\mathbf{0}$), pair ($\mathbf{2}$), parallel ($\downdownarrows$), and (co)sink ($[\leftrightarrow]\ast$) are associated with four types of (co)limits: (initial) terminal, (coproduct) product, (coequalizer) equalizer, and (pushout) pullback, respectively. The other, singleton ($\mathbf{1}$), shape category is associated with the identity (co)limit functor, which is always guaranteed

to exist, so this case is not detailed here. We provide the definition of the specific (co)limit from the definition of a general (co)limit by setting $\mathbf{J}$ to the associated shape, and show how the (co)limit is obtained via the correspondingly specific diagonal functor as part of a correspondingly specific comma category in Diagram 31 (limit) and Diagram 32 (colimit), and the correspondingly specific limit functor, which leads to recovery of specific (diagonal, limit) and (colimit, diagonal) adjoints from Diagram 33 and Diagram 34.

Terminal (initial)

The definition of a terminal limit is obtained by substituting $\mathbf{J} = \mathbf{0}$ into the definition of a limit. Hence, a terminal limit is a limit of an empty shaped diagram $D : \mathbf{0} \to \mathbf{C}$. Since the empty shape category $\mathbf{J} = \mathbf{0}$ contains no objects, or morphisms, the terminal limit as a universal cone $(\underleftarrow{L}, \kappa)$ has no (*leg*) morphisms $\kappa(j)$ other than the identity morphism $1_{\underleftarrow{L}}$. Hence, natural transformation κ is the morphism family containing just the (identity) morphism $1_{\underleftarrow{L}}$. Likewise for any cone (V, ϕ) to D, ϕ contains just the identity morphism 1_V. Thus, from the definition of a general limit, we obtain the following definition of a specific, terminal limit.

A *terminal limit* of an empty shaped diagram $D : \mathbf{0} \to \mathbf{C}$ is a universal cone $(\underleftarrow{L}, \kappa)$ to D containing just the identity morphism $1_{\underleftarrow{L}}$, such that for any cone (V, ϕ) to D, containing just 1_V, there exists a unique morphism $u : V \to \underleftarrow{L}$, as indicated by the following diagram:

$$\begin{array}{c} V \\ \Big\downarrow{\scriptstyle u} \\ \underleftarrow{L} \end{array} \tag{35}$$

Diagram 35 recovers the typical definition of a terminal object by relabeling limit object $\underleftarrow{L}$ as 1 and V as A in the main text: A terminal object in a category $\mathbf{C}$ is an object, denoted 1, such that for every object $Z \in |\mathbf{C}|$ there exists a unique morphism $u : Z \to 1$. In **Set**, any one-element (singleton) set is a terminal object.

From Diagram 31 we see that a terminal limit is a universal morphism derived from the comma category $(\Delta_{\mathbf{0}} \downarrow S_*)$, where $\Delta_{\mathbf{0}} : \mathbf{C} \to \mathbf{C}^{\mathbf{0}}$ is the diagonal functor specific to the category of empty diagrams, $\mathbf{C}^{\mathbf{0}} \cong \mathbf{1}$, containing just one object (denoted $*$), and its identity morphism (denoted 1_*), and S_* is the constant functor selecting the empty diagram and its identity morphism. Hence, $\Delta_{\mathbf{0}}$ is the

constant functor $\Delta_{\mathbf{0}} : A \mapsto *, f \mapsto 1_*$, for all A, f in $\mathbf{C}$. The construction of a terminal limit $(1, 1_*)$ as a universal morphism from comma category $(\Delta_{\mathbf{0}} \downarrow S_Y)$, and as a terminal object in that category is indicated in the following diagram:

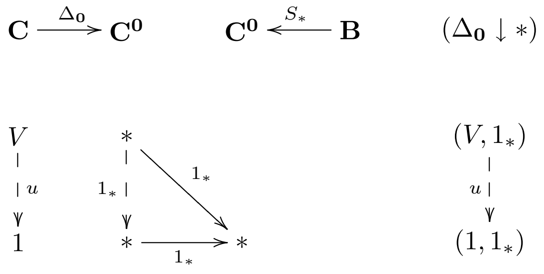

(36)

The right adjoint to $\Delta_{\mathbf{0}}$ is the specific limit functor $\underleftarrow{Lim}_{\mathbf{0}} : \mathbf{C}^{\mathbf{0}} \to \mathbf{C}$ from the category containing the empty diagram, selecting terminal object 1 and its identity morphism 1_1 in $\mathbf{C}$. That is, $\underleftarrow{Lim}_{\mathbf{0}} : * \mapsto 1, 1_* \mapsto 1_1$, and hence for a given object $X \in |\mathbf{C}|$, the unit of the adjunction is $f : X \to 1$, the unique arrow from X to the terminal object. This adjoint situation arises from the comma category correspondence $(1_{\mathbf{C}} \downarrow \underleftarrow{Lim}_{\mathbf{0}})/(\Delta_{\mathbf{0}} \downarrow 1_{\mathbf{C}^{\mathbf{0}}})$, as indicated in the following diagram:

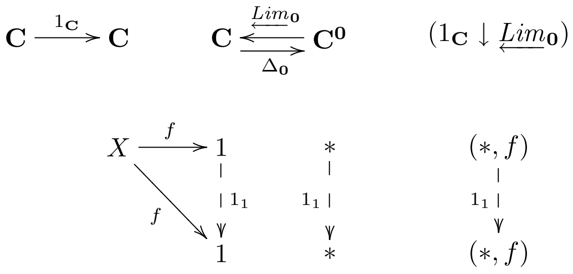

(37)

which commutes trivially. That is, from the definition of an adjunction (see Diagram 12), there is only one $Y = *$ and one $g = 1_*$ for category $\mathbf{D} = \mathbf{C}^{\mathbf{0}}$, hence g exists uniquely. Since $G(Y) = 1$ there can only be one $f : X \to 1$, since 1 is a terminal object, which is just the unit of the adjunction n_X, and since $G(g) = 1_1$, $1_1 \circ f = f$, and the diagram commutes.

The adjunction derived from the perspective of the counit is given in the following diagram:

$$\mathbf{C} \underset{\underleftarrow{Lim}_{\mathbf{0}}}{\overset{\Delta_{\mathbf{0}}}{\rightleftarrows}} \mathbf{C}^{\mathbf{0}} \qquad \mathbf{C}^{\mathbf{0}} \xleftarrow{1_{\mathbf{C}^{\mathbf{0}}}} \mathbf{C}^{\mathbf{0}} \qquad (\Delta_{\mathbf{0}} \downarrow 1_{\mathbf{C}^{\mathbf{0}}}) \tag{38}$$

$$\begin{array}{ccc} X & * & (X, 1_*) \\ \downarrow f & 1_* \downarrow \searrow 1_* & f \downarrow \\ 1 & * \xrightarrow[1_*]{} * & (1, 1_*) \end{array}$$

where $1_* : * \rightarrow *$ is the counit.

The definition of an initial colimit is similarly obtained by substituting $\mathbf{J} = \mathbf{0}$ into the definition of a colimit. Again, since $\mathbf{0}$ has no objects or morphisms, the initial colimit as a couniversal cocone $(\underrightarrow{L}, \chi)$ has no leg morphisms. Thus, from the definition of a general colimit, we obtain the following definition of a specific, initial colimit.

An *initial colimit* of an empty shaped diagram $D : \mathbf{0} \rightarrow \mathbf{C}$ is a couniversal cocone $(\underrightarrow{L}, \chi)$ from D containing just the identity morphism $1_{\underrightarrow{L}}$, such that for any cocone (W, ψ) from D, containing just 1_W, there exists a unique morphism $u : \underrightarrow{L} \rightarrow W$, as indicated by the following diagram:

$$\begin{array}{c} \underrightarrow{L} \\ \downarrow u \\ W \end{array} \tag{39}$$

which commutes trivially.

Diagram 39 recovers the definition typically given for an initial object by relabeling colimit object $\underrightarrow{L}$ as 0 and W as A in the main text: An *initial object* in category $\mathbf{C}$ is an object, denoted 0, such that for every object $Z \in |\mathbf{C}|$ there exists a unique morphism $u : 0 \rightarrow Z$. In **Set**, the empty set $\varnothing$ is the only initial object.

From Diagram 32, substituting $\Delta_{\mathbf{0}}$ for Δ, we see that an initial colimit is a couniversal morphism derived from the comma category $(X \downarrow \Delta_{\mathbf{0}})$. The construction of an initial colimit $(0, 1_*)$ as a couniversal morphism from comma category $(T_* \downarrow \Delta_{\mathbf{0}})$, and as an initial object in that category is indicated in the

following diagram:

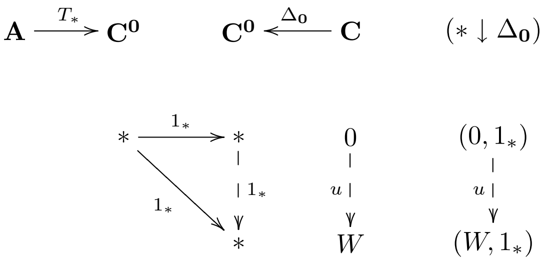

(40)

where T_* is a constant functor selecting diagram $*$.

The left adjoint to $\Delta_{\mathbf{0}}$ is the specific colimit functor $\underrightarrow{Lim}_{\mathbf{0}} : \mathbf{C}^{\mathbf{0}} \to \mathbf{C}$ from the category containing the empty diagram, selecting initial object 0 and its identity morphism 1_0 in $\mathbf{C}$. That is, $\underrightarrow{Lim}_{\mathbf{0}} : * \mapsto 0, 1_* \mapsto 1_0$, and hence for the only object $* \in |\mathbf{C}^{\mathbf{0}}|$, the unit of the adjunction is $1_* : * \to *$. This adjoint situation arises from the comma category correspondence $(1_{\mathbf{C}^{\mathbf{0}}} \downarrow \Delta_{\mathbf{0}})/(\underrightarrow{Lim}_{\mathbf{0}} \downarrow 1_{\mathbf{C}})$, as indicated in the following diagram:

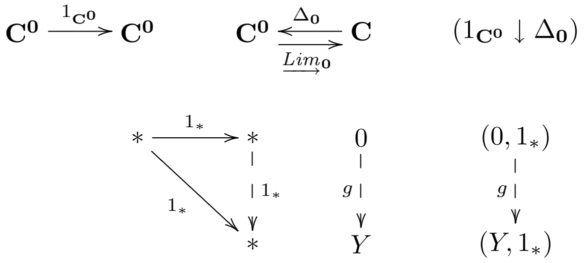

(41)

which commutes trivially. From the definition of an adjunction, $F(X) = \underrightarrow{Lim}_{\mathbf{0}}(*) = 0$, which is an initial object, hence for any $Y \in |\mathbf{C}|$, there must exist a unique g, and since $\Delta_{\mathbf{0}}(g) = 1_*$, $1_* \circ 1_* = 1_*$, so the diagram commutes.

From the perspective of the counit, the adjunction is indicated by the following diagram:

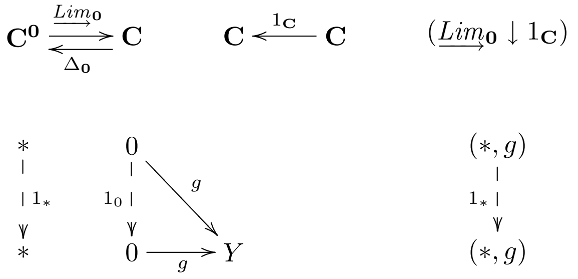

(42)

where $g : 0 \to Y$ is the counit.

Product (coproduct)

The definition of a product limit is obtained by substituting $\mathbf{J} = \mathbf{2}$ into the definition of a limit (see Diagram 28). Hence, a product limit is a limit of a diagram $D : \mathbf{2} \to \mathbf{C}$, whose shape is a pair. Thus, from the definition of a general limit, we obtain the following definition of a specific, product limit.

A *product limit* of pair shaped diagram $D : \mathbf{2} \to \mathbf{C}$ is a universal cone $(\underleftarrow{L}, \kappa)$ to D, which is a morphism family containing just two leg morphisms $\kappa_j : \underleftarrow{L} \to D(j)$, where $j \in \{I, J\}$, such that for any cone (V, ϕ) to D, containing just the two leg morphisms $\phi_j : V \to D(j)$, there exists a unique morphism $u : V \to \underleftarrow{L}$, such that the following diagram commutes:

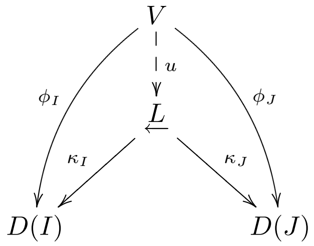

(43)

(There is no morphism between $D(I)$ and $D(J)$ because there are no non-identity morphisms in $\mathbf{2}$.)

Relabeling $D(I)$ and $D(J)$ as A and B (respectively), limit object $\underleftarrow{L}$ as product object P (or, $A \times B$), and V as Z in Diagram 43 recovers the typical definition given for a product: A product of two objects A and B in category $\mathbf{C}$ is an object P (also denoted $A \times B$) together with two morphisms $p_1 : P \to A$ and $p_2 : P \to B$, denoted (P, p_1, p_2), such that for every object $Z \in |\mathbf{C}|$ and pair of morphisms $f : Z \to A$ and $g : Z \to B$ there exists a unique morphism $u : Z \to P$, also denoted $\langle f, g \rangle$, such that the following diagram commutes:

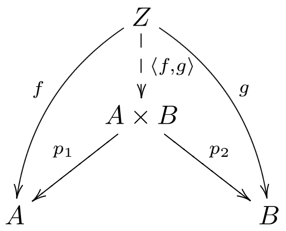

(44)

A product limit is a universal morphism derived from the comma category $(\Delta_{\mathbf{2}} \downarrow (A, B))$, where $\Delta_{\mathbf{2}} : \mathbf{C} \to \mathbf{C}^{\mathbf{2}}$ is the diagonal functor specific to the category of pair diagrams, $\mathbf{C}^{\mathbf{2}} \cong \mathbf{C} \times \mathbf{C}$, whose objects are pairs, (A, B), and morphisms are pairs of arrows, $(f, g) : (A_1, B_1) \to (A_2, B_2)$, where $\Delta_{\mathbf{2}}$:

$A \mapsto (A,A), f \mapsto (f,f)$, and $S_{(A,B)}$ is a constant functor selecting pair (A,B). The construction of a product limit (P,p_1,p_2) as a universal morphism from comma category $(\mathbf{\Delta_2} \downarrow (A,B))$, and as a terminal object in that category is indicated in the following diagram:

$$\begin{array}{ccc} \mathbf{C} \xrightarrow{\Delta_2} \mathbf{C^2} & \mathbf{C^2} \xleftarrow{S_{(A,B)}} \mathbf{B} & (\mathbf{\Delta_2} \downarrow (A,B)) \\ \\ \begin{array}{c} V \\ \downarrow \langle f,g\rangle \\ A\times B \end{array} & \begin{array}{ccc} (V,V) & & \\ \downarrow (\langle f,g\rangle,\langle f,g\rangle) & \searrow^{(f,g)} & \\ (A\times B, A\times B) & \xrightarrow[(p_1,p_2)]{} & (A,B) \end{array} & \begin{array}{c} (V,(f,g)) \\ \downarrow \langle f,g\rangle \\ (A\times B,(p_1,p_2)) \end{array} \end{array} \tag{45}$$

The right adjoint to $\mathbf{\Delta_2}$ is the specific limit functor $\underleftarrow{Lim}_\mathbf{2} : \mathbf{C^2} \to \mathbf{C}$, usually denoted as product functor Π, where $\Pi : (A,B) \mapsto A \times B$, $(f,g) \mapsto f \times g$, and the unit of the adjunction is $\langle 1_X, 1_X\rangle : X \to X \times X$. Note that $\times$ does not signify multiplication: in **Set**, the Cartesian product $A \times B$ of two sets A and B, together with two projection maps $p_1 : A \times B \to A, (a,b) \mapsto a$ and $p_2 : A \times B \to B, (a,b) \mapsto b$, is a product. This adjoint situation arises from the comma category correspondence $(1_\mathbf{C} \downarrow \Pi)/(\mathbf{\Delta_2} \downarrow 1_{\mathbf{C^2}})$, as indicated in the following commutative diagram:

$$\begin{array}{ccc} \mathbf{C} \xrightarrow{1_\mathbf{C}} \mathbf{C} & \mathbf{C} \underset{\Delta_2}{\overset{\Pi}{\rightleftarrows}} \mathbf{C^2} & (1_\mathbf{C} \downarrow \Pi) \\ \\ \begin{array}{ccc} X & \xrightarrow{\langle 1_X,1_X\rangle} & X\times X \\ & {}_{\langle f,g\rangle}\searrow & \downarrow f\times g \\ & & A\times B \end{array} & \begin{array}{c} (X,X) \\ \downarrow (f,g) \\ (A,B) \end{array} & \begin{array}{c} ((X,X),\langle 1_X,1_X\rangle) \\ \downarrow (f,g) \\ ((A,B),\langle f,g\rangle) \end{array} \end{array} \tag{46}$$

From the perspective of the counit, the adjunction is given in the following diagram:

$$\begin{array}{ccc} \mathbf{C} \underset{\Pi}{\overset{\Delta_2}{\rightleftarrows}} \mathbf{C^2} & \mathbf{C^2} \xleftarrow{1_{\mathbf{C^2}}} \mathbf{C^2} & (\mathbf{\Delta_2} \downarrow 1_{\mathbf{C^2}}) \\ \\ \begin{array}{c} X \\ \downarrow \langle f,g\rangle \\ A\times B \end{array} & \begin{array}{ccc} (X,X) & & \\ \downarrow (\langle f,g\rangle,\langle f,g\rangle) & \searrow^{(f,g)} & \\ (A\times B, A\times B) & \xrightarrow[(p_1,p_2)]{} & (A,B) \end{array} & \begin{array}{c} (X,(f,g)) \\ \downarrow \langle f,g\rangle \\ (A\times B,(p_1,p_2)) \end{array} \end{array} \tag{47}$$

where $(p_1, p_2) : (A \times B, A \times B) \rightarrow (A, B)$ is the counit.

A *coproduct colimit* of pair shaped diagram $D : \mathbf{2} \rightarrow \mathbf{C}$ is a couniversal cocone $(\underrightarrow{L}, \chi)$ from D, which is a morphism family containing just two leg morphisms $\chi_j : D(j) \rightarrow W$, where $j \in \{I, J\}$, such that for any cocone (W, ψ) from D, containing just the two leg morphisms $\psi_j : D(j) \rightarrow W$, there exists a unique morphism $u : \underrightarrow{L} \rightarrow W$, such that the following diagram commutes:

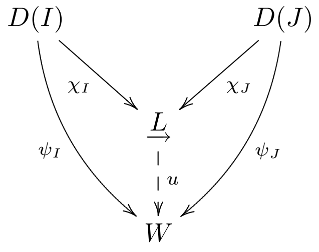

(48)

Relabeling $D(I)$ and $D(J)$ as A and B (respectively), colimit object $\underrightarrow{L}$ as coproduct object Q (or, $A + B$), and W as Z in Diagram 48 recovers the typical definition given for a coproduct: A coproduct of two objects A and B in category $\mathbf{C}$ is an object Q (also denoted $A + B$) together with two morphisms $q_1 : A \rightarrow Q$ and $q_2 : B \rightarrow Q$, denoted (Q, q_1, q_2), such that for every object $Z \in |\mathbf{C}|$ and pair of morphisms $f : A \rightarrow Z$ and $g : B \rightarrow Z$ there exists a unique morphism $u : Q \rightarrow Z$, also denoted $[f, g]$, such that the following diagram commutes:

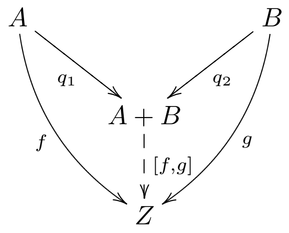

(49)

A coproduct colimit is a couniversal morphism derived from $((A, B) \downarrow \Delta_{\mathbf{2}})$, where $T_{(A,B)}$ selects (A, B). The construction of a coproduct colimit (Q, q_1, q_2) as a couniversal morphism from comma

category $((A,B) \downarrow \mathbf{\Delta_2})$, and as an initial object in that category is indicated in the following diagram:

$$\mathbf{A} \xrightarrow{T_{(A,B)}} \mathbf{C^2} \qquad \mathbf{C^2} \xleftarrow{\mathbf{\Delta_2}} \mathbf{C} \qquad ((A,B) \downarrow \mathbf{\Delta_2}) \tag{50}$$

$$\begin{array}{ccc} (A,B) & \xrightarrow{(q_1,q_2)} & (A+B, A+B) \\ & \searrow^{(f,g)} & \downarrow{\scriptstyle ([f,g],[f,g])} \\ & & (W,W) \end{array} \qquad \begin{array}{c} A+B \\ {\scriptstyle [f,g]}\downarrow \\ W \end{array} \qquad \begin{array}{c} (A+B,(q_1,q_2)) \\ {\scriptstyle [f,g]}\downarrow \\ (W,(f,g)) \end{array}$$

The left adjoint to $\mathbf{\Delta_2}$ is the specific colimit functor $\underrightarrow{Lim}_{\mathbf{2}} : \mathbf{C^2} \to \mathbf{C}$, usually denoted as coproduct functor II, where $\amalg : (A,B) \mapsto A+B$, $(f,g) \mapsto f+g$, and the unit of the adjunction is $(q_1,q_2) : (A,B) \to (A+B, A+B)$. Note that $+$ does not signify addition: in **Set**, it is realized as "disjoint union". That is, if $A, B \in |\mathbf{Set}|, A+B = \{(a,1)|A\} \cup \{(b,2)|B\}$, together with two injection maps $A \to A+B, a \mapsto (a,1)$ and $B \to A+B, b \mapsto (b,2)$, is a coproduct. This adjoint situation arises from the comma category correspondence $(1_{\mathbf{C^2}} \downarrow \mathbf{\Delta_2})/(\amalg \downarrow 1_{\mathbf{C}})$, as indicated in the following commutative diagram:

$$\mathbf{C^2} \xrightarrow{1_{\mathbf{C^2}}} \mathbf{C^2} \qquad \mathbf{C^2} \underset{\amalg}{\overset{\mathbf{\Delta_2}}{\rightleftarrows}} \mathbf{C} \qquad (1_{\mathbf{C^2}} \downarrow \mathbf{\Delta_2}) \tag{51}$$

$$\begin{array}{ccc} (A,B) & \xrightarrow{(q_1,q_2)} & (A+B, A+B) \\ & \searrow^{(f,g)} & \downarrow{\scriptstyle ([f,g],[f,g])} \\ & & (Y,Y) \end{array} \qquad \begin{array}{c} A+B \\ {\scriptstyle [f,g]}\downarrow \\ Y \end{array} \qquad \begin{array}{c} (A+B,(q_1,q_2))) \\ {\scriptstyle [f,g]}\downarrow \\ (Y,(f,g)) \end{array}$$

From the perspective of the counit, the adjunction is given in the following diagram:

$$\mathbf{C^2} \underset{\mathbf{\Delta_2}}{\overset{\amalg}{\rightleftarrows}} \mathbf{C} \qquad \mathbf{C} \xleftarrow{1_{\mathbf{C}}} \mathbf{C} \qquad (\amalg \downarrow 1_{\mathbf{C}}) \tag{52}$$

$$\begin{array}{c} (A,B) \\ \downarrow{\scriptstyle (f,g)} \\ (Y,Y) \end{array} \qquad \begin{array}{ccc} A+B & & \\ {\scriptstyle f+g}\downarrow & \searrow^{[f,g]} & \\ Y+Y & \xrightarrow[{[1_Y,1_Y]}]{} & Y \end{array} \qquad \begin{array}{c} ((A,B),[f,g]) \\ {\scriptstyle (f,g)}\downarrow \\ ((Y,Y),[1_Y,1_Y]) \end{array}$$

where $[1_Y, 1_Y] : Y+Y \to Y$ is the counit.

Equalizer (coequalizer)

An *equalizer limit* of parallel shaped diagram $D : \downdownarrows \rightarrow \mathbf{C}$ is a universal cone $(\underleftarrow{L}, \kappa)$ to D, which is a morphism family containing just two leg morphisms $\kappa_j : \underleftarrow{L} \rightarrow D(j)$, where $j \in \{I, J\}$, such that for any cone (V, ϕ) to D, containing just the two leg morphisms $\phi_j : V \rightarrow D(j)$, there exists a unique morphism $u : V \rightarrow \underleftarrow{L}$, such that the following diagram commutes:

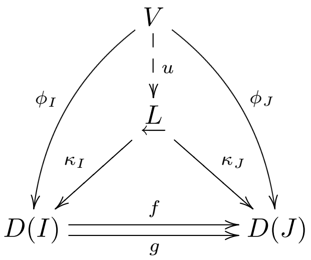

(53)

(There are two parallel morphisms between objects $D(I)$ and $D(J)$ corresponding to the parallel morphisms in the parallel shape category.)

Relabeling $D(I)$ and $D(J)$ as A and B (respectively), limit object $\underleftarrow{L}$ as equalizer object E, and V as Z in Diagram 53 recovers the typical definition given for an equalizer: an equalizer of two morphisms $f, g : A \rightarrow B$ in category $\mathbf{C}$ is an object $E \in |\mathbf{C}|$ together with a morphism $e : E \rightarrow A$, denoted (E, e), such that for every object $Z \in \mathbf{C}$ and morphism $z : Z \rightarrow A$ there exists a unique morphism $u : Z \rightarrow E$, such that the following diagram commutes:

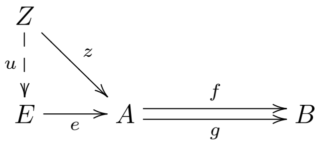

(54)

where the morphisms corresponding to ϕ_J and κ_J are omitted, because they are implied by $f \circ z$ (and $g \circ z$) and $f \circ e$ (and $g \circ e$), respectively. In **Set**, $E = \{a \in A | f(a) = g(a)\}$ and e is the inclusion map $E \rightarrow A$.

An equalizer limit is a universal morphism derived from the comma category $(\Delta_{\downdownarrows} \downarrow S_{(f,g)})$, where $\Delta_{\downdownarrows} : \mathbf{C} \rightarrow \mathbf{C}^{\downdownarrows}$ is the diagonal functor specific to the category of parallel diagrams, $\mathbf{C}^{\downdownarrows}$, whose objects are parallel arrows, (f, g) and morphisms are pairs of arrows, $(h, k) : (f_1, g_1) \rightarrow (f_2.g_2)$, such that $f_2 \circ h = k \circ f_1$

and $g_2 \circ h = k \circ g_1$, as indicated in the following diagram:

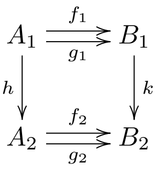

(55)

where $\Delta_{\downdownarrows} : A \to (1_A, 1_A)$ and $\Delta_{\downdownarrows} : f \to (f, f)$, and $S_{(f,g)}$ is a constant functor selecting (f, g).

The construction of equalizer limit (E, e) as a universal morphism from comma category $(\Delta_{\downdownarrows} \downarrow (f, g))$, and as a terminal object in that category is indicated in the following diagram:

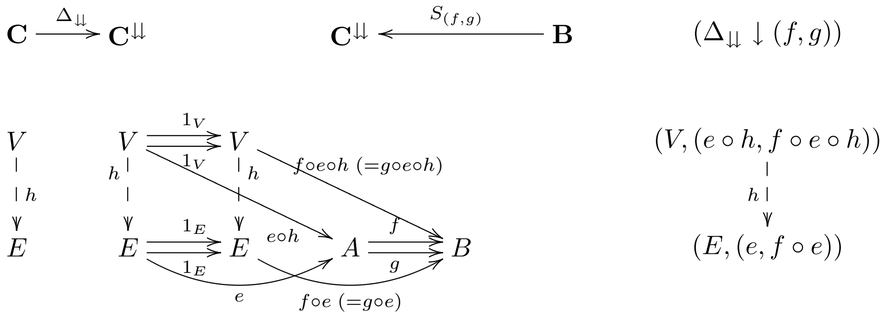

(56)

The right adjoint to $\Delta_{\downdownarrows}$ is the specific limit functor $\varprojlim_{\downdownarrows} : \mathbf{C}^{\downdownarrows} \to \mathbf{C}$, and the unit of the adjunction is $1_X : X \to X$. This adjoint situation arises from the comma category correspondence $(1_{\mathbf{C}} \downarrow \varprojlim_{\downdownarrows})/(\Delta_{\downdownarrows} \downarrow 1_{\mathbf{C}^{\downdownarrows}})$, as indicated in the following commutative diagram:

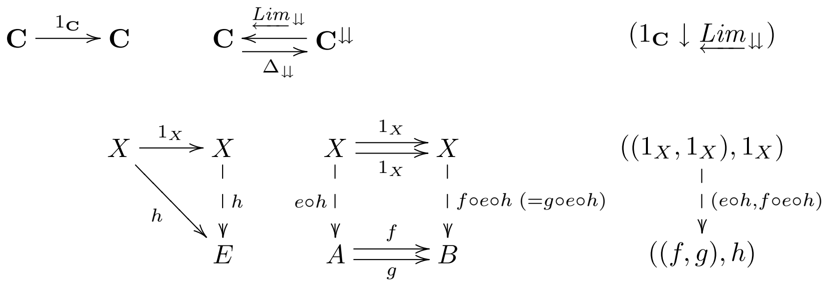

(57)

From the perspective of the counit, the adjunction is given in the following diagram:

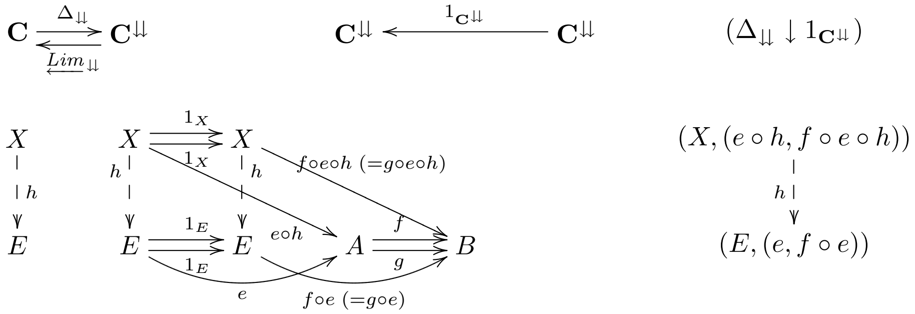

(58)

where $(e, f \circ e) = (e, g \circ e) : (1_E, 1_E) \to (f, g)$ is the counit.

A *coequalizer colimit* of parallel shaped diagram $D : \downdownarrows \to \mathbf{C}$ is a couniversal cocone $(\underrightarrow{L}, \chi)$ from D, which is a morphism family containing just two leg morphisms $\chi_j : D(j) \to W$, where $j \in \{I, J\}$, such that for any cocone (W, ψ) from D, containing just the two leg morphisms $\psi_j : D(j) \to W$, there exists a unique morphism $u : \underrightarrow{L} \to W$, such that the following diagram commutes:

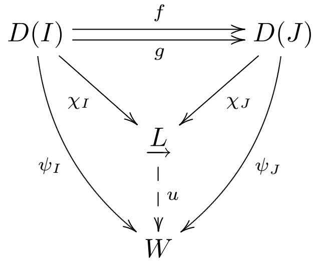

(59)

Relabeling $D(I)$ and $D(J)$ as A and B (respectively), colimit object $\underrightarrow{L}$ as coequalizer object Q, and W as Z in Diagram 59 recovers the typical definition given for a coequalizer: A coequalizer of two morphisms $f, g : A \to B$ in category $\mathbf{C}$ is an object Q together with a morphism $q : B \to Q$, denoted (Q, q), such that for every object $Z \in |\mathbf{C}|$ and morphism $z : B \to Z$ and $g : B \to Z$ there exists a unique morphism $u : Q \to Z$, such that the following diagram commutes:

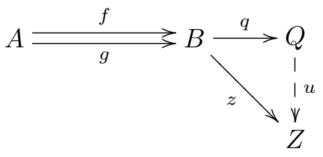

(60)

where the morphisms corresponding to ψ_I and χ_I are omitted, because they are implied by $z \circ f$ (and

$z \circ g$) and $q \circ f$ (and $q \circ g$), respectively. In **Set**, Q is the set of equivalence classes $\{[b]|b \in B\}$, where $[b]$ is the equivalence class under the relation $\overline{E}$, which is the reflexive, symmetric, transitive closure of $E \subseteq B \times B$, where $E = \{(f(a), g(a))|a \in A\}$.

A coequalizer colimit is a couniversal morphism derived from the comma category $(T_{(f,g)} \downarrow \Delta_{\downdownarrows})$, where $T_{(f,g)}$ is a constant functor selecting a parallel diagram (f, g). The construction of a coequalizer colimit (Q, q) as a couniversal morphism from comma category $((f, g) \downarrow \Delta_{\downdownarrows})$, and as an initial object in that category is indicated in the following diagram:

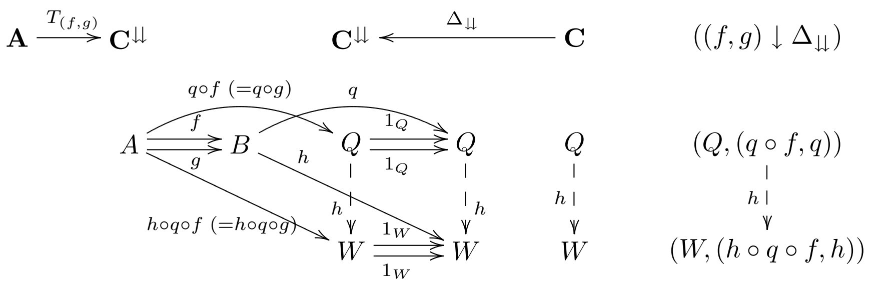

(61)

The left adjoint to $\Delta_{\downdownarrows}$ is the specific colimit functor $\underrightarrow{Lim}_{\downdownarrows} : \mathbf{C}^{\downdownarrows} \rightarrow \mathbf{C}$. This adjoint situation arises from the comma category correspondence $(1_{\mathbf{C}^{\downdownarrows}} \downarrow \Delta_{\downdownarrows})/(\underrightarrow{Lim}_{\downdownarrows} \downarrow 1_{\mathbf{C}})$, as indicated in the following commutative diagram:

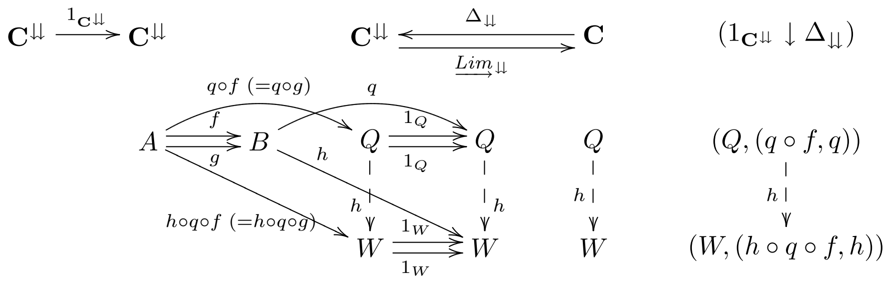

(62)

From the perspective of the counit, the adjunction is given in the following diagram:

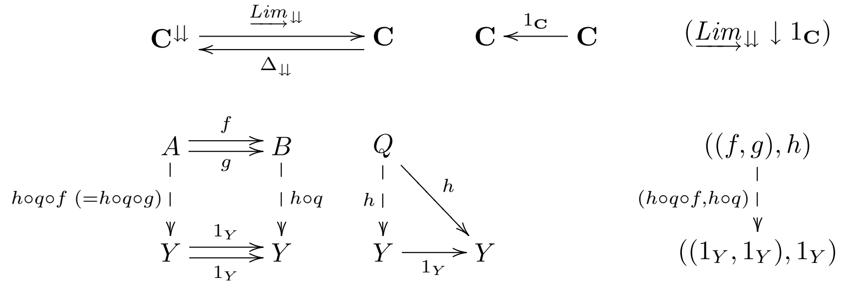

(63)

where $1_Y : Y \to Y$ is the counit.

Pullback (pushout)

The definition of a pullback limit is obtained by substituting $\mathbf{J} = \divideontimes$ into the definition of a limit. Hence, a pullback limit is a limit of a sink shaped diagram $D : \divideontimes \to \mathbf{C}$. Thus, from the definition of a general limit, we obtain the following definition of a specific, pullback limit.

A *pullback limit* of sink shaped diagram $D : \divideontimes \to \mathbf{C}$ is a universal cone $(\underleftarrow{L}, \kappa)$ to D, which is a morphism family containing just three leg morphisms $\kappa_j : \underleftarrow{L} \to D(j)$, where $j \in \{I, J, K\}$, such that for any cone (V, ϕ) to D, containing just the three leg morphisms $\phi_j : V \to D(j)$, there exists a unique morphism $u : V \to \underleftarrow{L}$, such that the following diagram commutes:

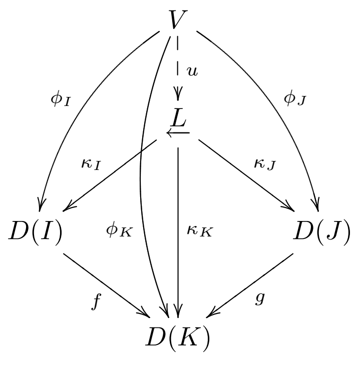

(64)

Relabeling $D(I)$, $D(J)$, and $D(K)$ as A, B, and C (respectively), limit object $\underleftarrow{L}$ as pullback object P (or, $A \times_C B$), and V as Z in Diagram 64 recovers the typical definition given for a pullback: A pullback of two morphisms $f : A \to C$ and $g : B \to C$ in category $\mathbf{C}$ is an object P (also denoted $A \times_C B$) together with two morphisms $p_1 : P \to A$ and $p_2 : P \to B$, denoted (P, p_1, p_2), such that for every object $Z \in |\mathbf{C}|$

and pair of morphisms $z_1 : Z \to A$ and $z_2 : Z \to B$ there exists a unique morphism $u : Z \to P$, also denoted $\langle f, g \rangle$, such that the following diagram commutes:

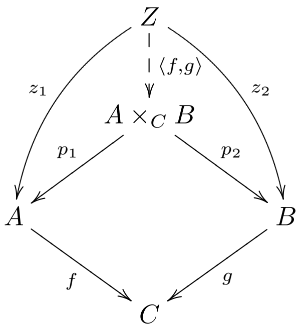

(65)

where the morphisms corresponding to ϕ_K and κ_K are omitted, because they are implied by $f \circ z_1$ (and $g \circ z_2$) and $f \circ p_1$ (and $g \circ p_2$), respectively. In **Set**, $A \times_C B = \{(a, b) \in A \times B | f(a) = g(b)\}$, together with two projection maps $p_1 : A \times_C B \to A, (a, b) \mapsto a$ and $p_2 : A \times_C B \to B, (a, b) \mapsto b$, is a pullback.

A pullback limit is a universal morphism derived from the comma category $(\Delta_{⁕} \downarrow (f, g))$, where $\Delta_{⁕} : \mathbf{C} \to \mathbf{C}^{⁕}$ is the diagonal functor specific to the category of sink diagrams, $\mathbf{C}^{⁕}$, whose objects are sinks, $A \xrightarrow{f} C \xleftarrow{g} B$, denoted (f, g), and morphisms are arrows $(h, k) : (f_1, g_1) \to (f_2, g_2)$, such that the following diagram commutes:

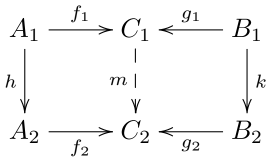

(66)

where $\Delta_{⁕} : A \mapsto (1_A, 1_A), f \mapsto (f, f)$. (Reference to middle arrow $m : C_1 \to C_2$ is omitted, because it is determined by the other arrows.) The construction of a pullback limit (P, p_1, p_2) as a universal morphism from comma category $(\Delta_{⁕} \downarrow (f, g))$, and as a terminal object in that category is indicated in the following diagram:

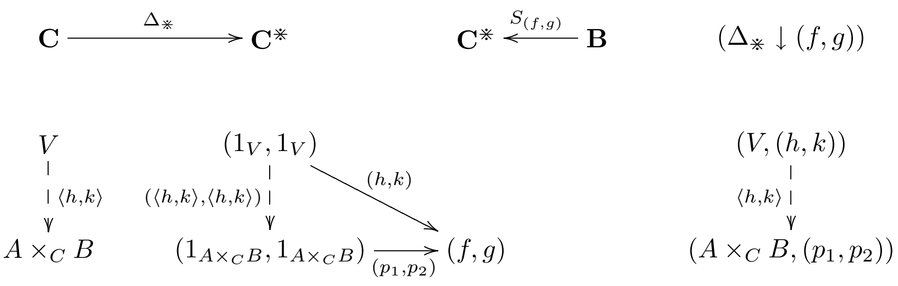

(67)

where $S_{(f,g)}$ is a constant functor selecting sink diagram (f,g).

The right adjoint to $\Delta_{\ast}$ is the specific limit functor $\underleftarrow{Lim}_{\ast} : \mathbf{C}^{\ast} \to \mathbf{C}$, denoted as pullback functor Π_C, where $\Pi_C : (A,B) \mapsto A \times_C B$, $(f,g) \mapsto f \times g$, and the unit of the adjunction is $\langle 1_X, 1_X \rangle : X \to X \times_X X$. This adjoint situation arises from the comma category correspondence $(1_{\mathbf{C}} \downarrow \Pi_C)/(\Delta_{\ast} \downarrow 1_{\mathbf{C}^{\ast}})$, as indicated in the following commutative diagram:

$$\mathbf{C} \xrightarrow{1_{\mathbf{C}}} \mathbf{C} \qquad \mathbf{C} \underset{\Delta_{\ast}}{\overset{\Pi_C}{\rightleftarrows}} \mathbf{C}^{\ast} \qquad (1_{\mathbf{C}} \downarrow \Pi_C) \tag{68}$$

$$\begin{array}{ccc} X \xrightarrow{\langle 1_X, 1_X \rangle} X \times_X X & (1_X, 1_X) & ((1_X, 1_X), \langle 1_X, 1_X \rangle) \\ {\scriptstyle \langle h,k \rangle} \searrow \quad \big\downarrow {\scriptstyle h \times k} & {\scriptstyle (h,k)} \big\downarrow & {\scriptstyle (h,k)} \big\downarrow \\ A \times_C B & (f,g) & ((f,g),(h,k)) \end{array}$$

where $X \times_X X = X \times X$.

From the perspective of the counit, the adjunction is given in the following diagram:

$$\mathbf{C} \underset{\Pi_C}{\overset{\Delta_{\ast}}{\rightleftarrows}} \mathbf{C}^{\ast} \qquad \mathbf{C}^{\ast} \xleftarrow{1_{\mathbf{C}^{\ast}}} \mathbf{C}^{\ast} \qquad (\Delta_{\ast} \downarrow 1_{\mathbf{C}^{\ast}}) \tag{69}$$

$$\begin{array}{ccc} X & (1_X, 1_X) \quad \searrow^{(h,k)} & (X, (h,k)) \\ \big\downarrow {\scriptstyle \langle h,k \rangle} & {\scriptstyle (\langle h,k \rangle, \langle h,k \rangle)} \big\downarrow & {\scriptstyle \langle h,k \rangle} \big\downarrow \\ A \times_C B & (1_{A \times_C B}, 1_{A \times_C B}) \xrightarrow[(p_1,p_2)]{} (f,g) & (A \times_C B, (p_1, p_2)) \end{array}$$

where $(p_1, p_2) : (1_{A \times_C B}, 1_{A \times_C B}) \to (f,g)$ is the counit, and $p_1 : A \times_C B \to A$ and $p_2 : A \times_C B \to B$.

A *pushout colimit* of cosink shaped diagram $D : \leftarrow\!\!\cdot\!\!\to \ \to \mathbf{C}$ is a couniversal cocone $(\underrightarrow{L}, \chi)$ from D, which is a morphism family containing just three leg morphisms $\chi_j : D(j) \to W$, where $j \in \{I, J, K\}$, such that for any cocone (W, ψ) from D, containing just the three leg morphisms $\psi_j : D(j) \to W$, there exists a

unique morphism $u : \underrightarrow{L} \rightarrow W$, such that the following diagram commutes:

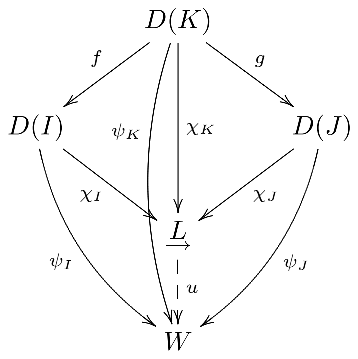

(70)

Relabeling $D(I)$, $D(J)$, and $D(K)$ as A, B, and C (respectively), colimit object $\underrightarrow{L}$ as pushout object Q (or, $A +_C B$), and W as Z in Diagram 70 recovers the typical definition given for a pushout: A pushout of two morphisms $f : C \rightarrow A$ and $g : C \rightarrow B$ in category $\mathbf{C}$ is an object Q (also denoted $A +_C B$) together with two morphisms $q_1 : A \rightarrow Q$ and $q_2 : B \rightarrow Q$, denoted (Q, q_1, q_2), such that for every object $Z \in |\mathbf{C}|$ and pair of morphisms $z_1 : A \rightarrow Z$ and $z_2 : B \rightarrow Z$ there exists a unique morphism $u : Q \rightarrow Z$, also denoted $[f, g]$, such that the following diagram commutes:

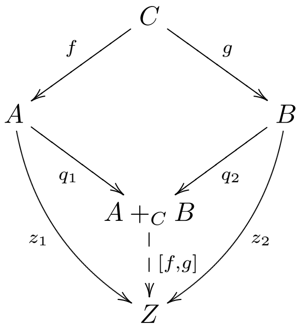

(71)

where the morphisms corresponding to ψ_K and χ_K are omitted, because they are implied by $z_1 \circ f$ (and $z_2 \circ g$) and $q_1 \circ f$ (and $q_2 \circ g$), respectively. In **Set**, a pushout is obtained by forming the coproduct of A and B and then forming the equivalence classes under the reflexive, symmetric, transitive closure of the relation $\{(q_1(f(c)), q_2(g(c)))|c \in C\}$.

A pushout colimit is a couniversal morphism derived from $((f, g) \downarrow \Delta_{\leftrightarrow})$, where $T_{(f,g)}$ selects (f, g). The construction of a pushout colimit (Q, q_1, q_2) as a couniversal morphism from comma category $((f, g) \downarrow$

$\Delta_{\leftrightarrow}$), and as an initial object in that category is indicated in the following diagram:

$$\mathbf{A} \xrightarrow{T_{(f,g)}} \mathbf{C}^{\leftrightarrow} \qquad \mathbf{C}^{\leftrightarrow} \xleftarrow{\ \Delta_{\leftrightarrow}\ } \mathbf{C} \qquad ((f,g) \downarrow \Delta_{\leftrightarrow}) \tag{72}$$

$$\begin{array}{ccccc} (f,g) \xrightarrow{(q_1,q_2)} & (1_{A+_C B}, 1_{A+_C B}) & A +_C B & (A +_C B, (q_1,q_2)) \\ {\scriptstyle (h,k)} \searrow & \downarrow {\scriptstyle ([h,k],[h,k])} & {\scriptstyle [h,k]} \downarrow & {\scriptstyle [h,k]} \downarrow \\ & (1_W, 1_W) & W & (W, (h,k)) \end{array}$$

The left adjoint to $\Delta_{\leftrightarrow}$ is the specific colimit functor $\underrightarrow{Lim}_{\leftrightarrow} : \mathbf{C}^{\leftrightarrow} \to \mathbf{C}$, usually denoted as pushout functor II, where $\amalg : (A,B) \mapsto A+B$, $(f,g) \mapsto f+g$, and the unit of the adjunction is $(q_1,q_2) : (f,g) \to (1_{A+_C B}, 1_{A+_C B})$, where $q_1 : A \to A+B$ and $q_2 : B \to A+B$. This adjoint situation arises from the comma category correspondence $(1_{\mathbf{C}^{\leftrightarrow}} \downarrow \Delta_{\leftrightarrow})/(\amalg \downarrow 1_{\mathbf{C}})$, as indicated in the following commutative diagram:

$$\mathbf{C}^{\leftrightarrow} \xrightarrow{1_{\mathbf{C}^{\leftrightarrow}}} \mathbf{C}^{\leftrightarrow} \qquad \mathbf{C}^{\leftrightarrow} \underset{\amalg_C}{\overset{\Delta_{\leftrightarrow}}{\rightleftarrows}} \mathbf{C} \qquad (1_{\mathbf{C}^{\leftrightarrow}} \downarrow \Delta_{\leftrightarrow}) \tag{73}$$

$$\begin{array}{ccccc} (f,g) \xrightarrow{(q_1,q_2)} & (1_{A+_C B}, 1_{A+_C B}) & A +_C B & (A +_C B, (q_1,q_2)) \\ {\scriptstyle (h,k)} \searrow & \downarrow {\scriptstyle ([h,k],[h,k])} & {\scriptstyle [h,k]} \downarrow & {\scriptstyle [h,k]} \downarrow \\ & (1_Y, 1_Y) & Y & (Y, (h,k)) \end{array}$$

From the perspective of the counit, the adjunction is given in the following diagram:

$$\mathbf{C}^{\leftrightarrow} \underset{\Delta_{\leftrightarrow}}{\overset{\amalg_C}{\rightleftarrows}} \mathbf{C} \qquad \mathbf{C} \xleftarrow{1_{\mathbf{C}}} \mathbf{C} \qquad (\amalg_C \downarrow 1_{\mathbf{C}}) \tag{74}$$

$$\begin{array}{cccc} (f,g) & A +_C B & & ((f,g),[h,k]) \\ \downarrow {\scriptstyle (h,k)} & {\scriptstyle h+k} \downarrow \quad \searrow {\scriptstyle [h,k]} & & {\scriptstyle (h,k)} \downarrow \\ (1_Y, 1_Y) & Y +_C Y \xrightarrow[{[1_Y,1_Y]}]{} Y & & ((1_Y,1_Y),[1_Y,1_Y]) \end{array}$$

where $[1_Y, 1_Y] : Y +_C Y \to Y$ is the counit, and $Y +_C Y = Y + Y$.

References

1. Mac Lane S (2000) Categories for the working mathematician. Graduate Texts in Mathematics. New York, NY: Springer, 2nd edition.

2. Phillips S, Wilson WH (2010) Categorial compositionality: A category theory explanation for the systematicity of human cognition. PLoS Computational Biology 6: e1000858.

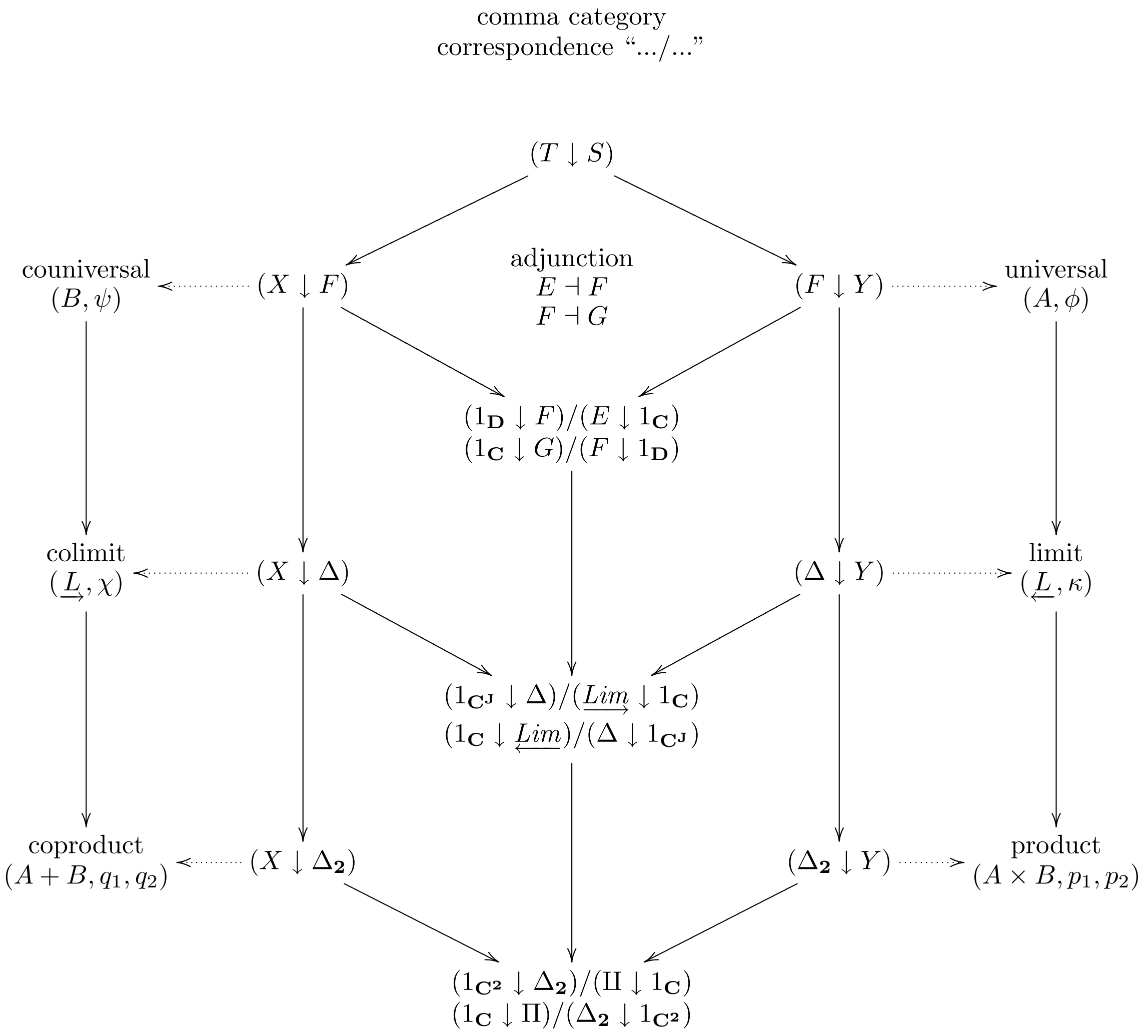

Figure 1. Comma categories and (co)universal constructions. Solid arrows connecting cells point to special cases, dotted arrows point to component constructs. The only special cases of (co)limits shown are (co)products. The upper row of each of the four center column cells pertaining to adjunction is associated with couniversal morphisms, and the lower row with universal morphisms, e.g., $(1_{\mathbf{C}^{\mathbf{2}}} \downarrow \Delta_{\mathbf{2}})$, etc. is associated with the coproduct.

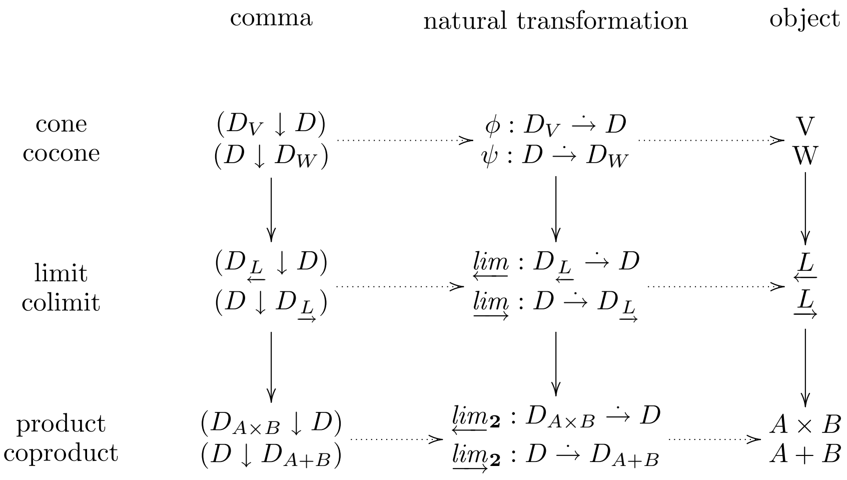

Figure 2. Comma categories and component constructs. Solid arrows connecting cells point to special cases, dotted arrows point to component constructs. The only special cases of (co)limits shown are (co)products.
