# Supplementary material for: Categorial Compositionality II: Universal Constructions and a General Theory of (Quasi-)Systematicity in Human Cognition
Source: PLoS Comput Biol. 2011 Aug 4;7(8):e1002102. doi: 10.1371/journal.pcbi.1002102 (PMC3154512; doi:10.1371/journal.pcbi.1002102)
Supplement: Text S3 — An explanation for systematicity in “relational schema induction” using adjunction. (PDF) [file pcbi.1002102.s026.pdf]

Text S3

Relational schema induction: Universal construction

In the relational schema induction paradigm, participants are given a series of task instances conforming to a common group-like structure. Each task instance consists of (shape, trigram) pairs from which they must predict the resulting trigram. Systematicity with respect to this cognitive domain refers to the observation that the capacity to do one task instance implied the capacity to do other instances [1].

This paradigm was modeled as a (free, forgetful) adjoint, where the free functor $F : \mathbf{Set} \times \mathbf{Set} \rightarrow \mathbf{ASet}$ generates the free $ASet$, modeling a task instance, on the pair of sets (Q, X) containing the set of triagrams Q and set of shapes X, used for that task [2]. The forgetful functor $U : \mathbf{ASet} \rightarrow \mathbf{Set} \times \mathbf{Set}$ returns the underlying sets, forgetting their compositions. The (F, U) adjoint pair, defined in terms of the unit of the adjunction, is indicated in the following diagram:

$$\begin{array}{ccc c c} (Q, X) & \xrightarrow{\eta_{(Q,X)}} & (Q \times X^*, X) & \qquad & (Q \times X^*, X, \mu) \\ & \searrow^{(g,\rho)} & \downarrow U(\psi) & & \downarrow \psi \\ & & (R, Y) & & (R, Y, \gamma) \end{array} \tag{1}$$

where $\eta_{(Q,X)}(q, x) = ((q, \epsilon), x)$, and (Q, X) and (R, Y) are the (trigram, shape) pairs of sets for different instances of the task. This adjunction is also expressed in terms of the counit, as indicated by the diagram

$$\begin{array}{c c c c c} (Q, X) & \qquad & (Q \times X^*, X, \mu) & & \\ \downarrow (g,\rho) & & \downarrow F(g,\rho) & \searrow^{\psi} & \\ (R, Y) & & (R \times Y^*, Y, \nu) & \xrightarrow[\epsilon_{(R,Y)}]{} & (R, Y, \gamma) \end{array} \tag{2}$$

where the following diagram commutes (see [2], Text S1, Diagram 5):

$$\begin{array}{ccc} (R \times Y^*) \times Y & \xrightarrow{\nu_{(R,Y)}} & R \times Y^* \\ \downarrow \gamma^* \times 1_Y & & \downarrow \gamma^* \\ R \times Y & \xrightarrow[\gamma]{} & R \end{array} \tag{3}$$

So, from Diagram 2, $((R, Y), \epsilon_{(R,Y)})$ is a universal morphism, i.e., a terminal object in the comma category $(F \downarrow (R, Y, \gamma))$. From Diagram 1, $((Q \times X^*, X, \mu), \eta_{(Q,X)})$ is a couniversal morphism, i.e., an initial object

in the comma category $(U \downarrow (Q,X))$. The explanation for systematicity parallels the explanations for the other examples in the main text. All transfers from the first task instance, $(Q \times X^*, X, \mu)$ to any other task instance (R, Y, γ) is mediated by a common universal arrow. In terms of the transfer,(g, ρ), from the stimulus set for the first task instance, (Q, X), to the stimulus set for any other instance, (R, Y), the mediating arrow is $\eta_{(Q,X)}$. In terms of the transfer,ψ, from the set of stimulus mappings for the first task instance, $(Q \times X^*, X, \mu)$ to the set of stimulus mappings for any other instance, (R, Y, γ), the mediating arrow is $\epsilon_{(R,Y)}$. Therefore, given these arrows as part of the initial capacity, there is one and only one way to obtain all the other capacities.

References

1. Halford GS, Bain JD, Maybery MT, Andrews G (1998) Induction of relational schemas: Common processes in reasoning and complex learning. Cognitive Psychology 35: 201–245.

2. Phillips S, Wilson WH (2010) Categorial compositionality: A category theory explanation for the systematicity of human cognition. PLoS Computational Biology 6: e1000858.
