# Supplementary material for: Categorial Compositionality II: Universal Constructions and a General Theory of (Quasi-)Systematicity in Human Cognition
Source: PLoS Comput Biol. 2011 Aug 4;7(8):e1002102. doi: 10.1371/journal.pcbi.1002102 (PMC3154512; doi:10.1371/journal.pcbi.1002102)
Supplement: Text S4 — A category theory definition of systematicity. (PDF) [file pcbi.1002102.s027.pdf]

Text S4

A category theory ***definition*** of systematicity

In general, systematicity means that one has cognitive capacity c_1 *if and only if* one has cognitive capacity c_2 (e.g., one has the capacity to infer John as the lover from "John loves Mary" if and only if one has the capacity to infer Mary as the lover from "Mary loves John"). Thus, systematicity is essentially an *equivalence relation* on a set of cognitive capacities. Equivalence relations are identified with a particular kind of *coequalizer* in category theory [1], p60–63. Hence, category theory also provides a formal definition of systematicity in terms of a coequalizer. First, we provide a formal definition of an equivalence relation, and associated definitions of *equivalence class*, and *quotient set.* Then, we show how an equivalence relation is identified with a coequalizer, thus yielding a formal category theory definition of systematicity.

An *equivalence relation* on a set A is a relation $R \subseteq A \times A$, such that R is:

- *reflexive*: i.e., aRa, for all $a \in A$;
- *symmetric*: i.e., a_1Ra_2 if and only if a_2Ra_1; and
- *transitive*: i.e., if a_1Ra_2 and a_2Ra_3, then a_1Ra_3.

An *R–equivalence class* of an element $a_i \in A$ is the set $[a_i] = \{a_j | a_iRa_j\}$, which contains all the elements of A that are R–related to a_i.

The *quotient set* of a set A by a relation R is the set $A/R = \{[a] | a \in A\}$.

A *coequalizer* of two morphisms $f, g : A \to B$ in category $\mathbf{C}$ is an object Q together with a morphism $q : B \to Q$, denoted (Q, q), such that for every object $Z \in |\mathbf{C}|$ and morphism $z : B \to Z$, there exists a unique morphism $u : Q \to Z$, such that the following diagram commutes:

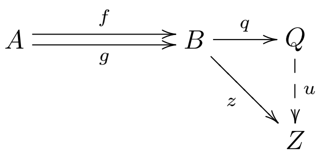

(1)

An equivalence relation R on a set A is identified with the following coequalizer diagram:

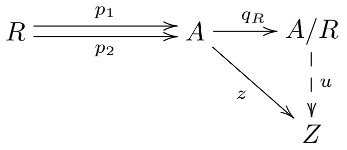

(2)

where $p_1 : (a_1, a_2) \mapsto a_1$ and $p_2 : (a_1, a_2) \mapsto a_2$ are projections, and $q_R : a \mapsto [a]$ identifies each element of A with its equivalence class. (Note that q_R is a couniversal arrow, see Text S2, and identifies equivalence classes in a "minimal" way, i.e., with no more or less classes than necessary.)

Given a set of cognitive capacities C, *systematicity* is an equivalence relation $S \subseteq C \times C$, identified with the coequalizer indicated in the following commutative diagram:

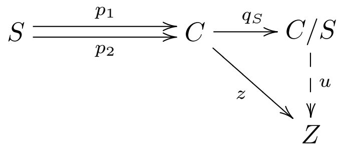

(3)

where each $[c] \in C/R$ is a set (group) of indivisibly linked (i.e., systematically related) cognitive capacities.

A coequalizer, expressed in Diagram 1, is equivalent to a kind of *pushout* (see Text S2, for a definition), expressed in the following diagram:

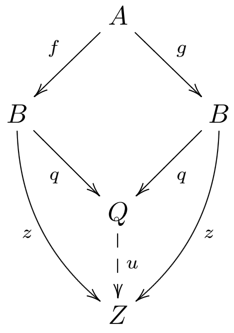

(4)

Our explanation of systematicity in terms of adjunction also satisfies this category theory definition of systematicity. Recall (from Text S2) that in an adjoint situation, $F \dashv G$, where functors $F : \mathbf{C} \to \mathbf{D}$ and $G : \mathbf{D} \to \mathbf{C}$, every object $X \in |\mathbf{C}|$ and $Y \in |\mathbf{D}|$ is associated with a (co)universal morphism, $(G \circ F(X), \eta_X)$ and $(F \circ G(Y), \epsilon_Y)$, respectively. Consider that the groups of cognitive capacities in question correspond to the hom-sets parameterized by X or Y (cf. Text S2, Diagram 16 and surrounding text). For instance, the capacities to infer agent and patient from propositions such as *John loves Mary* correspond to the

morphisms that belong to the hom-set $\hom_{\mathbf{C}^2}((Pr, Pr), (S, S))$, from Diagram 9, which contains all the morphisms with (S, S) as their codomain. Each morphism (ag, pt) in this hom-set factors through the universal morphism (p_1, p_2). It is easy to show that the universal morphism must be unique with respect to this property. Hence, all morphisms in the hom-set are related to each other by a common factor, i.e., the universal morphism. In general, the counit $\epsilon : F \circ G \dot{\rightarrow} 1_{\mathbf{D}}$ for adjoint situation $F \dashv G$ contains one (universal) morphism $\epsilon_Y : F \circ G(Y) \rightarrow Y$ for each $Y \in \mathbf{D}$, and is identified with an equivalence relation R_ϵ on set $\{(f, g) | f, g \text{ morphisms in } \mathbf{D}\}$, where $R_\epsilon = \{(f, g) | \exists Y, \exists f', \exists g', f = \epsilon_Y \circ f', g = \epsilon_Y \circ g'\}$. Evidently, this relation is reflexive, symmetric, and transitive, and so defines equivalence classes, $[\epsilon_Y] = \{f | \epsilon_Y R_\epsilon f\}$, where $f : B \rightarrow Y$. In the general case of modelling multiple relations, either implicitly with distinct pairs of constituent objects (A, B), or with an explicit relation symbol, as in $(R_i, (A, B))$ (see [2], Diagram 17), there is one universal morphism for each relation, and therefore, one equivalence class for each group of cognitive capacities associated with that relation. Hence, our explanation for systematicity in terms of adjunctions also satisfies our category theory definition of systematicity. (The same situation applies for capacities in terms of couniversal morphisms.)

References

1. Goldblatt R (2006) Topoi: The categorial analysis of logic. NY: New York: Dover Publications, revised edition.

2. Phillips S, Wilson WH (2010) Categorial compositionality: A category theory explanation for the systematicity of human cognition. PLoS Computational Biology 6: e1000858.
